# Identification of breast lesion through integrated study of gorilla troops optimization and rotation-based learning from MRI images

**DOI:** 10.1038/s41598-023-36300-3

**Published:** 2023-07-18

**Authors:** Tapas Si, Dipak Kumar Patra, Saurav Mallik, Anjan Bandyopadhyay, Achyuth Sarkar, Hong Qin

**Affiliations:** 1 Department of Computer Science & Engineering, University of Engineering & Management, Jaipur, GURUKUL, Sikar Road (NH-11), Udaipuria Mod, Jaipur, Rajasthan 303807 India; 2 Department of Computer Science, Hijli College, Kharagpur, West Bengal 721306 India; 3 Department of Environmental Health, Harvard T H Chan School of Public Health, Boston, MA USA; 4 School of Computer Engineering, Kalinga Institute of Industrial Technology (KIIT), Bhubaneswar, Odisha India; 5 Department of Computer Science & Engineering, National Institute of Technology Arunachal Pradesh, Arunachal Pradesh, 791113 India; 6 Department of Computer Science and Engineering, University of Tennessee at Chattanooga, Chattanooga, TN USA

**Keywords:** Image processing, Machine learning

## Abstract

Breast cancer has emerged as the most life-threatening disease among women around the world. Early detection and treatment of breast cancer are thought to reduce the need for surgery and boost the survival rate. The Magnetic Resonance Imaging (MRI) segmentation techniques for breast cancer diagnosis are investigated in this article. Kapur’s entropy-based multilevel thresholding is used in this study to determine optimal values for breast DCE-MRI lesion segmentation using Gorilla Troops Optimization (GTO). An improved GTO, is developed by incorporating Rotational opposition based-learning (RBL) into GTO called (GTORBL) and applied it to the same problem. The proposed approaches are tested on 20 patients’ T2 Weighted Sagittal (T2 WS) DCE-MRI 100 slices. The proposed approaches are compared with Tunicate Swarm Algorithm (TSA), Particle Swarm Optimization (PSO), Arithmetic Optimization Algorithm (AOA), Slime Mould Algorithm (SMA), Multi-verse Optimization (MVO), Hidden Markov Random Field (HMRF), Improved Markov Random Field (IMRF), and Conventional Markov Random Field (CMRF). The Dice Similarity Coefficient (DSC), sensitivity, and accuracy of the proposed GTO-based approach is achieved $87.04\%$, $90.96\%$, and $98.13\%$ respectively. Another proposed GTORBL-based segmentation method achieves accuracy values of $99.31\%$ , sensitivity of $95.45\%$ , and DSC of $91.54\%$. The one-way ANOVA test followed by Tukey HSD and Wilcoxon Signed Rank Test are used to examine the results. Furthermore, Multi-Criteria Decision Making is used to evaluate overall performance focused on sensitivity, accuracy, false-positive rate, precision, specificity, $F_1$-score, Geometric-Mean, and DSC. According to both quantitative and qualitative findings, the proposed strategies outperform other compared methodologies.

## Introduction

Breast cancer in women is one of the most frequent cancers worldwide^1^, and it is the main reason why women die. In comparison to developed countries, mortality rates in low and middle-income countries are comparatively high. In 2020 breast cancer was being diagnosed in worldwide 2.3 million women, with 685,000 deaths owing to the disease. “As of the end of 2020, there were 7.8 million women alive who were diagnosed with breast cancer in the past 5 years, making it the world’s most prevalent cancer”^2^.

In high-income nations, early diagnosis and treatment have been demonstrated to be beneficial, and it should be done in low-income countries with minimal equipment. Breast cancer survival rates range from 40 percent in South Africa and 66 percent in India to more than 90 percent in high-income nations five years after diagnosis. In nations that have been successful in lowering it, the annual mortality rate from breast cancer has fallen by 2-4 percent. If global mortality rates decreased by 2.5 percent annually between 2020 and 2040, 2.5 million breast cancer deaths might be avoided.

Furthermore, in rural regions, a scarcity of medical specialists and experts exacerbates the difficulty of early and accurate breast cancer diagnosis, leading to a higher mortality rate.

Medical imaging testing is the most effective method for detecting breast cancer^3^. Digital mammography^4,5^, ultrasound^6^, magnetic resonance imaging^7–13,14,15–17,18^, microscopic slices, and infrared thermogram^19^ are some of the medical imaging modalities used for diagnosis. These modalities create images that have lowered mortality rates by 30–70%^20^ as a technique of assisting radiologists and clinicians in recognising problems. Because image interpretation is operator-dependent and requires skill, information technology is required to speed up and improve the accuracy of diagnosis while also offering a second opinion to the expert^21^. Computer-Aided Diagnosis (CAD)^22^, which uses computerised characteristics extraction and classification algorithms, can be a very useful tool for physicians in spotting anomalies.

CAD systems based on breakthroughs in digital artificial intelligence, image processing, and pattern recognition have been developed with great effort. CAD systems are predicted to lower the cost of medical auxiliary modalities, eliminate operator dependency, and boost diagnosis rate^23–25^ As a result, it may assist to prevent false positive rates, which can lead to ineffective treatment as well as psychological, physical, and financial expenses. It may also prevent erroneous negative readings, which could lead to treatment omissions and remissions. According to reports, detection sensitivity without CAD is around 80 percent, and sensitivity with it is around 90%^26^. According to the findings, CAD paired with mammography had 100 percent sensitivity in detecting malignancies appearing as microcalcifications and 86 percent sensitivity for other mammographic cancer presentations. As a result, CAD has emerged as the most effective area of research in medical imaging for improving diagnosis precision^27,28^.

The use of a computer output to establish the location of questionable lesions is known as computer aided detection. Following that, radiologists are responsible for the diagnosis of abnormalities, as well as patient care. On the other hand, computer aided diagnosis takes a human or a machine’s detection and produces an output that defines the nature of the lesion, as well as the likelihood of malignancy and any abnormalities^29^.

We introduced a new segmentation approach based on GTO^30^, which was first proposed in 2021. It is much more successful than its competitors at finding better solutions to complex optimization issues. As a result, we’re eager to apply the GTO to breast DCE-MRI segmentation. Our main goal is to use the GTO to segment breast lesions in fat-suppressed DCE-MRI in order to produce better results than existing approaches. GTO is employed in Kapur entropy maximization to segment the breast T2 WS DCE-MRI for lesion identification in the current study. The performance of the proposed method is compared with PSO^31^, MVO^32^, SMA^33^, AOA^34^, TSA^35^, IMRF^36^, HMRF^37^, and CMRF^38^ methods. Accuracy, sensitivity, specificity, geometric-mean, precision, false-positive rate, $F_1$-score, and DSC are all used to evaluate performance. The data are statistically analysed using two different statistical analysis methods: one-way ANOVA^39^ test followed by post-hoc Tukey HSD^40^ test and Wilcoxon Signed Rank Test^41^ test. Furthermore, for overall performance evaluation, Multi-Criteria Decision Analysis (MCDA)^42^ based on the aforementioned eight performance metric, i.e. criteria is employed, and this style of performance analysis is adapted from the study^43,44^. The proposed methods outperform the eight competing methods, according to the examination of the experimental findings employing the aforementioned three analysis.

### Contributions of this article

The following is a summary of the contributions:

1. Two methods for breast lesion segmentation in fat-suppressed DCE-MRI utilising GTO has been devised. Before now, GTO had never been used for breast T2 WS DCE-MRI segmentation. In the first method, classical GTO is used. We developed an enhanced GTO with RBL and used it in the second method.
2. Experiments conducted with 100 slices of the breast DCE-MRI, and the findings were analysed using a set of pertinent metrics, including sensitivity, accuracy, precision, specificity, $F_1$-score, geometric-mean, DSC, False-Positive Rate (FPR), and convergence.
3. The metaheuristics such as MVO, PSO, SMA, AOA, and TSA, and three existing breast MRI segmentation methods such as CMRF, IMRF, and HMRF are compared with the proposed methods in this study.
4. Discussion of the outcomes using statistical and multi-criteria decision-making techniques.
5. Eight competitive methods are outperformed by the proposed approaches.

### Organization of this article

The following is how the rest of the paper is organised: Sect.  Related Works explains the related works. The materials and procedures are discussed in Sect. Materials and methodology. This section includes the GTO, proposed GTORBL algorithms, and proposed segmentation methods. The experimental setup is described at Sect. Setup for experimentation. The results and discussion are provided in Sect. Results & discussion. Finally, Sect. conclusion provides a conclusion as well as prospective future works.

## Related works

Segmentation of the Region of Interest (ROI), feature extraction, and classification are the three phases of any CAD system for diagnosis of breast cancer. Segmentation is a crucial step in the creation of a CAD system. The purpose of ROI segmentation is to distinguish the breasts from the rest of the body. Because of the importance of breast cancer segmentation, more studies have been conducted in recent years. As a result, this section summarises previous work on breast cancer segmentation using the DCE-MR image collection.

For breast segmentation in MRI, Patra et al.^45^ produced a Grammatical Fireworks Algorithm (GFWA) technique. The GFWA clustering method is applied to segment MRI. The divided slices are separated from the lesions. The suggested technique’s experimental findings (both quantitative and qualitative) are analysed to those of Grammatical Swarm and K-means clustering techniques, and the approach outperforms both. On 25 T2 WS DCE-MRI slices, this approach is being tested. Si and Mukhopadhyay^46^ produced breast MRI segmentation for lesion diagnosis applying clustering through the Fireworks Algorithm (FWA). For lesion detection, a breast MRI segmentation technique based on improved hard-clustering with FWA has been produced. The segmentation approach is compared to PSO and K-means methods-based segmentation methods. Kar and Si^47^ used clustering with MVO approach to generate breast MRI segmentation for lesion identification. For lesion detection, a modified clustering with MVO is devised. The K-means and PSO clustering are used to compare the results. Sun et al.^48^ suggested a super voxel-based semi-supervised breast tumour segmentation algorithm. They employed cluster methods to detect the tumour location from MRI images and then used a supervised classifier to classify it.

Breast MRI segmentation focused on Student Psychological Based Optimization (SPBO) by thresholding employing Shannon entropy for lesion identification proposed by Patra et al.^43^. The MR slices are segmented using the SPBO method. After being retrieved from the segmented slices, the lesions are found in the original MR slices. On 300 T2 WS DCE-MRI slices that have been validated, this method is tested. The suggested segmentation approach has a 99.44% accuracy, a 96.84 percent sensitivity, and a DSC of 93.41%. Ibrahim et al.^49^ suggested a segmentation approach focused on the chaotic salp swarm algorithm (CSSA). CSSA is a superpixel extraction algorithm based on chaotic maps and created utilising the fast-shift approach to extract superpixels with CSSA-optimized parameters. The DMRIR dataset used to check the output of this approach. Sayed et al.^50^, proposed a CAD method that relies on automatic ROI segmentation. To obtain reliable results, the segmentation approach employs the advantages of four Bio-inspired swarming algorithms: PSO, Grey Wolf Optimization (GWO), Moth Flame Optimization (MFO), and Firefly algorithm (FA). Breast MRI segmentation focused on quasi opposition-based Sine Cosine Algorithm (SCAQOBL) by thresholding employing Shannon entropy for lesion identification proposed by Si et al.^51^. The MR slices are segmented using the SCAQOBL method. After being retrieved from the segmented slices, the lesions are found in the original MR slices. On 100 T2 WS DCE-MRI slices that have been validated, this method is tested. The suggested segmentation approach has a 99.11% accuracy, a 97.78 percent sensitivity, and a DSC of 95.42%. Breast MRI segmentation focused on opposition-based Chimp Optimization Algorithm (ChOAOBL) by thresholding employing Kapur entropy for lesion identification proposed by Si et al.^52^. The MR slices are segmented using the ChOAOBL method. On 200 T2 WS DCE-MRI slices that have been validated, this method is tested. The suggested segmentation approach has a 99.02% accuracy, a 95.73 percent sensitivity, and a DSC of 93.25%.

Azmi et al.^53^ suggested breast cancer segmentation in MRI based on the Markov Random Field (MRF) approach. This approach employs a traditional MRF, in which the energy function is determined solely on levels of nearby pixels, which are allocated at random. By considering the spatial relationship between neighbouring pixels, the MRF model produces good results. The PIDER breast MRI dataset use this approach. The mean values for specificity, sensitivity, precision, and accuracy are 93.76 percent, 93.76 percent, 79.74 percent, and 93.10 percent, respectively.

Liu et al.^54^ suggested a fully automated approach for bulk identification and segmentation of mammography slices. The approach uses a mass detection strategy that is split into two categories: (a) constructing a search template and (b) obtaining an image utilising template matching. Adaptive thresholding focused on the maximum entropy used to convert the image features into ROIs. The region-growing method used to eliminate mammography lumps from the backdrop. The technique was examined using 70 mammography slices from the Digital Database for Screening Mammography (DDSM). The suggested approach exhibited a sensitivity of 97.2 percent and 1.83 false positives. MRI-based breast tumor diagnosis created by Ha and Vahedi^55^ utilizing the modified Deer Hunting Optimization algorithm, which is focused on a feature-related technique and an improved convolutional neural network. The goal of this project is to make classification easier by using the preprocessing stage. Using a Haralick texture and local binary pattern to extract features is also a nice idea. The dataset consists of 219 breast MR slices taken from 105 distinct breast cancer patients. As a consequence of the obtained data, the accuracy of this technique is 98.89 percent, suggesting its excellent potential and efficiency. Keyvanfard et al.^56^ extracted texture, kinetic, and shape characteristics from the lesion-containing segmented ROI and then used various classifiers to distinguish between malignant neural regression networks, including probabilistic neural network, multi-layer perceptron, generalized neural regression network, Support Vector Machine (SVM), and radial basis function network. The best results were merged to create a multi-classifier approach with a 90% accuracy rating. AlQoud et al.^57^ implemented the technique of detecting breast cancer based on Gabor features and local binary pattern features. To diagnose breast cancer, the Artificial Neural Network (ANN) classification technique supervised here is used by classifying characteristics into normal tissues and abnormal tissues in the breast. To extract features from the ROI portion of the images, these methods use the adaptive k-means clustering technique. Here, the gray level co-occurrence matrix elements erase the pectoral muscle boundary. Finally, the irregular and regular breast tissues are identified using K-Nearest Neighbors (KNN) and SVM techniques. Dong et al.^58^ developed a unique breast cancer detection framework. The feature results from the ROIs were then extracted using the improved vector field convolution features. Finally, the sorts of classes, malignant, and benign, classified using the random forest classification technique. The main disadvantage of random forest was its intricacy, as well as the fact that it took longer to generate the decision trees. Punitha et al.^59^ offered an improved region-growing technique for automatic detection of breast masses. Texture information from the segmented images, such as the grey-level co-occurrence and run length matrix, retrieved and sent into the feed forward neural network as input. The performance of the recommended technique tested using a Gaussian filter to remove noise from 300 mammography slices. The specificity and sensitivity of the suggested approach were 97.8 percent and 98.1 percent, respectively. The performance was also 90.0 percent, according to the Jaccard index. Segmentation in mammography images and across-sensor comparison with customized and deep characteristics developed by Cardoso et al.^60^. The models in mammography images for mass segmentation are: (a) boundary computation and tailored features; (b) second and third models that are focused on deep learning features and combine smooth SVM and Conditional Random Field (CRF); and (c) a combined model based on tailored features and boundary computation. The cross-sensor performance loss is greater than 10.0 percent and the mammography slices are taken from DDSM-BCRP and INbreast.

Li et al.^61^ developed the dense U-Net for automatically segmenting breast cancer in mammography images. For the segmentation of breast mass, this approach uses an automatic segmentation technique focused on deep learning. For mammography segmentation, this technique combines Attention Gates (AGs) with densely linked U-Net. Additionally, the DDSM database used to test this method, and the findings proved that dense U-Net coupled with AGs performed better than other methodologies. The technique yielded an $F_1$-score of 82.24 percent, a sensitivity of 77.89 percent, and accuracy of 78.38 percent. Zeiser et al.^62^ developed a breast cancer diagnosis model based on U-Net. Data augmentation, image preprocessing, training, and testing are the four phases of the produced model. Following the acquisition of mammography slices from DDSM, image preprocessing utilised to improve contrast, obtain regions of interest, and remove extraneous information. Mirroring, scaling, and zooming of mammography slices were all done with data augmentation. Finally, the malignant and benign classifications were classified using the U-Net approach. The created model performed better in terms of accuracy, dice index, specificity, and sensitivity as evidenced by the experimental results. The location and orientation of the slices (lesion and non lesion areas) in the low-resolution mammography slice are not encoded by the U-Net model during detection. For early identification of breast cancer, Rampun et al.^63^ proposed a breast area and pectoral muscle segmentation method. To detect the breast boundary, the active contour model utilised initially, followed by a postprocessing method to rectify the overstated border caused by artefacts. The pectoral muscle boundary detected and noisy edges were removed using a canny edge detection procedure and a preprocessing methodology. Then, through contour growth, five edge features used to hunt for the true border. In comparison to manual segmentation, the devised method achieved substantial results using dice similarity coefficients. Bora et al.^64^ created a textural gradient approach for segmenting the pectoral muscle. The Hough transformation applied to estimate the pectoral edge on the expected textural gradient of the mammography image. The method used Euclidean distance regression and polynomial modelling to build smooth pectoral muscle curves and was resistant to overlapping and texturing fibro glandular tissues. According to the results of the trial, the approach performed better in terms of accuracy in pectoral muscle segmentation. Due to smaller size and decreased contrast, the developed approach failed to detect pectoral muscle from mammography images. Shen et al.^65^ presented a new method for segmenting the pectoral muscle region from images automatically. Genetic algorithm, image preprocessing, polynomial curve fitting, and morphological selection are the four phases of the produced model. The genetic algorithm utilised to learn multilayer thresholds in this study, and the morphological selection technique employed to find the best contour of pectoral muscle areas based on morphological data. The efficiency of the created model tested using the micro Mammographic Image Analysis Society (MIAS), DDSM, and INBreast databases in this work. Because of the dense gland and mass border, the detection rate in the generated model reduced.

Nanayakkara et al.^66^ offered a strategy for automatic mammography breast boundary segmentation. The skin line and breast segmentation estimated using a modified quick matching method and morphological operators. The recommended approach evaluated on 136 mammography images applying an alternating sequential filter to eliminate noise. The recommended technique had a 99.2 percent ground truth sensitivity and a 99.0 percent segmentation accuracy. Desai et al.^67^ suggested a method for detecting microcalcification in mammography by refining the segmentation approach. The approach uses a modified multiscale morphological gradient watershed segmentation in mammograms for identification of clustered microcalcifications. The efficiency of the suggested technique was assessed using 2 databases: MIAS and Nuclear Magnetic Resonance (NMR). Adaptive median filter used to reduce noise. The true positive rates found to be 95.4 percent and 94.0 percent for 2 databases.

Isa and Siong^68^ developed automatic mass segmentation and identification in mammogram slices. The suggested approach splits mammography slices into two categories: (a) mass and their background pixels; and (b) Image enhancement increases the contrast of the image. The area of the breast mass discovered and segmented applying a region development focused on local statistical texture analysis. Total 322 mammography slices are tested to conduct this approach. The technique had a sensitivity of 94.59% and a FPR of 3.90 percent. Shrivastava et al.^69^ developed an automated digital mammography segmentation approach based on a scattered region expanding and sliding window algorithm. To detect worrisome masses in mammography slices, the system employs a fully automated technique. The pectoral muscles were removed from the mammography using the sliding window approach. The mammography images taken from the MIAS, and the suggested approach had a 91.3 percent accuracy.

Using Gray-Level Co-Occurrence Matrix (GLCM) texture and fuzzy c-means characteristics, Saleck et al.^70^ suggested cancer identification in mammogram slices. The FCM algorithm used in the suggested method as an automatic technique for bulk segmentation in mammography images. A median filter applied to eliminate noise from the 18 mammography images after they were downloaded from the MIAS database. The tumour was extracted from the ROI using the suggested method, which also included a fuzzy c-means algorithm; the FCM input was then validated using GLCM texture characteristics. The technique generated results with 96.4 percent specificity, 94.6 percent accuracy, and 86.2 percent sensitivity. Using an improved U-Net segmentation technique using mammography images, Hossain^71^ presented microcalcification segmentation. With mammogram slices obtained from the DDSM, the suggested approach was trained, and noise was eliminated employing the Laplacian filter. The procedure was broken down into five steps: preprocessing, breast area segmentation, breast region extraction, breast region selection of positive patches, and breast region training of the segmentation area. The technique generated a Dice score of 97.80% and F-measure of 98.50%. The value of Jaccard index is 97.40 percent, while the proposed method’s average accuracy is 98.20 percent.

The analysis of the literature reveals that tumour diagnosis is a tough and important undertaking. This paper proposes breast DCE-MRI segmentation based on entropy maximization using metaheuristics like GTO and GTORBL. The proposed approaches are explained in the next section.

## Materials and methodology

### DCE-MRI dataset

A total of 100 images which includes T2-W fat-suppressed DCE-MRI slices of 20 patients are collected from publicly accessible “The Cancer Genome Atlas Breast Invasive Carcinoma Collection” (TCGA-BRCA)^72,73^. Each slice is having the size $256\times 256$. The ground truths, regarded as the gold standard^74^, are generated by expert radiologists. T2-weighted images are a useful tool for MRI breast mass evaluation. By carefully analysing T2-weighted images, it is possible to lower the false-positive rates and distinguish uncommon, well-circumscribed breast cancers from frequent benign breast masses. Fluid-sensitive T2-W MRI show edoema, haemorrhage, mucus, or cystic fluid in contrast to T1-W MRI, which emphasise anatomic detail. Using T2-W MRI, characterization of breast lesions can be performed more accurately than T1-W MRI^75^. Therefore, T2-W MRI are selected for breast lesion identification in this present study. In this current work, the MRI dataset is formulated by including breast DCE-MR images of 20 different women patients having different size of breast lesions from small to large, and from single lesion regions to multiple lesion regions. DCE-MRIs of benign and malignant breast lesions are included in this dataset.

### Proposal

The following are the three steps of the proposal:

1. Pre-processing
2. Segmentation
3. Post-processing

**Figure 1:**
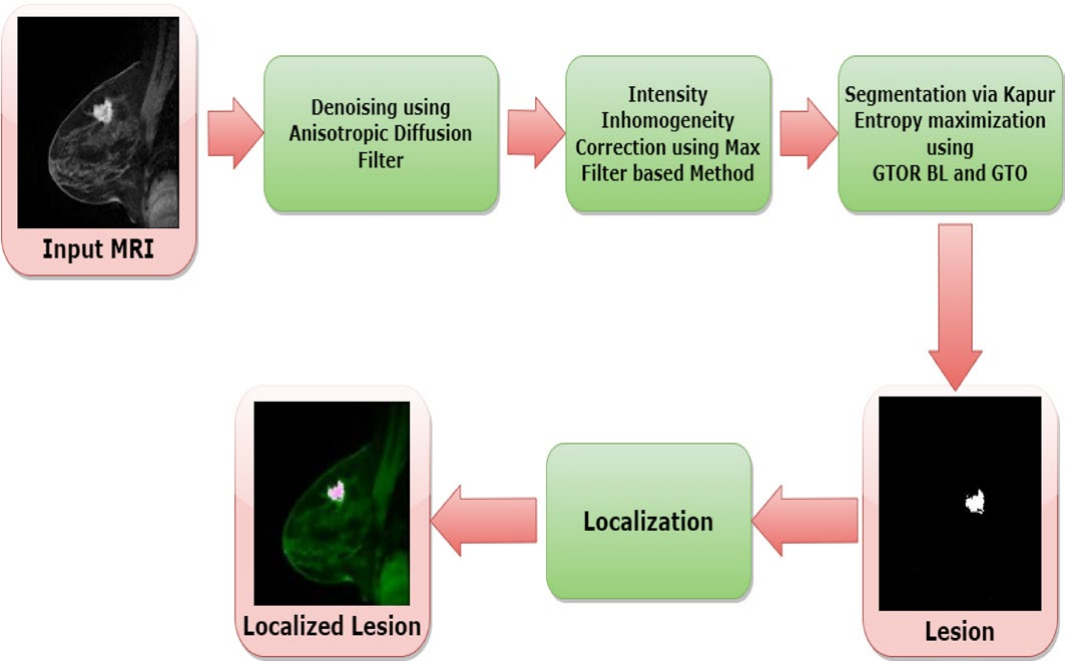
Outline of the proposed methods.

Figure 1 discussed flowchart of the proposed methods.

**Figure 2:**
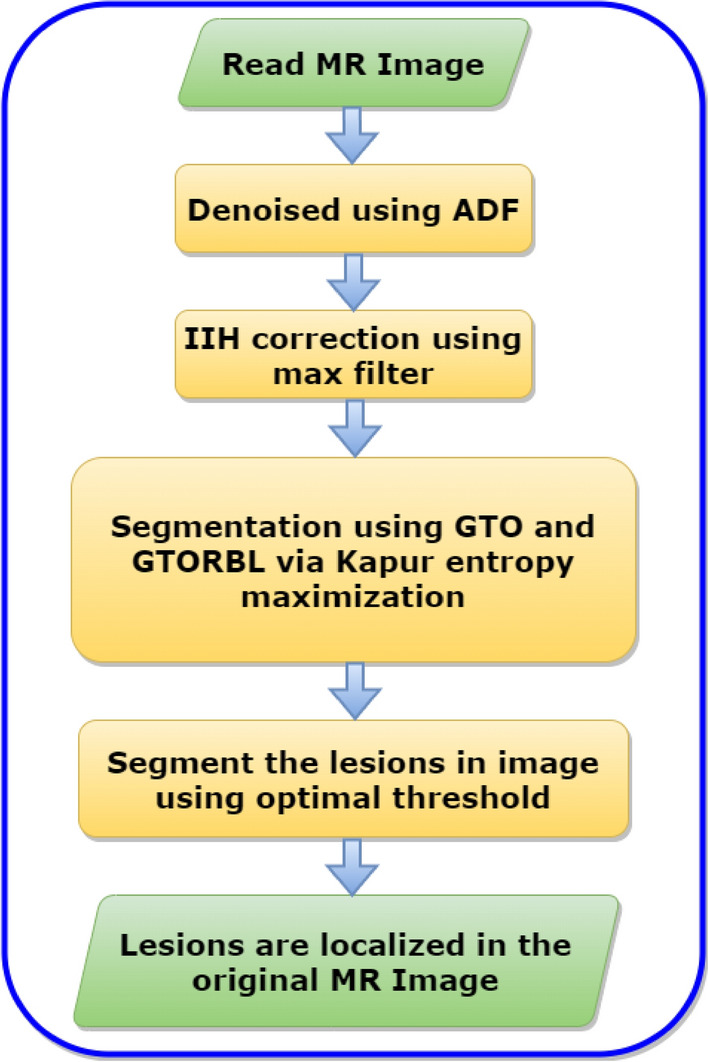
Flowchart of the Proposed Segmentation Algorithm.

The overall pseudocode and flowchart of the proposed method are given respectively in Algorithm 1 and Fig. 2. In step 1 MR images are read as input. Then in step 2 MR images are denoised using Anisotropic Diffusion Filter (ADF). We have performed denoising during the trial phase ADF, Median filter, and Contrast Limited Adaptive Histogram Equalization (CLAHE). ADF became best based on Peak Signal-Noise Ratio (PSNR) value. It is discussed on subsection 3.3. In step 3 Intensity Inhomogeneities (IIHs) correction using max filter based method. In step 4 MR images are segment using GTO and GTORBL via Kapur entropy maximization. After That in step 5 segment the lesions in the image using optimal threshold. In step 6 lesions are localized in original MR image.
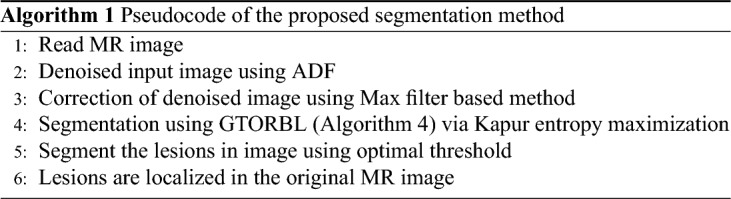

### Pre-processing

The visual quality of MRIs is crucial for accurate diagnosis and treatment of the patients, and it is affected by the presence of noise generated during the acquisition procedure. Both segmentation process^76^ and clinical diagnostic functions are affected by the noise. The Intensity Inhomogeneities (IIHs) in MRI slices also entangle the process of segmentation. IIHs in MRIs are the smooth intensity variations within a previously homogeneous region^77^. Median filter, Contrast Limited Adaptive Histogram Equalization (CLAHE)^78^, and Anisotropic Diffusion Filter (ADF)^79^ are tested to denoise the DCE-MRIs in the trial phase of this current study. It is noticed that mean peak signal noise ratio (PSNR)^80^ values are 20.89, 20.79, and 21.16, over 100 slices. Standard deviation values are 0.4314, 0.5404, and 0.0147. In this result, ADF becomes a better method for denoising than median filter and CLAHE. For that reason, ADF^76^ is opted to denoise the MRIs in the current study.

A Max filter^77^ is applied to correct the IIHs. The inhomogeneous image (${\mathscr {I}}_{ih}$) is represented as follows:

1 $$\begin{aligned} {\mathscr {I}}_{ih}={\mathscr {I}}_o \times {\mathscr {H}} + {\mathscr {N}} \end{aligned}$$

where ${\mathscr {H}}$ indicates an inhomogeneity bias field, ${\mathscr {N}}$ denotes noise, and a homogeneous image indicates ${\mathscr {I}}_o$.

### Segmentation

By separating DCE-MRI into non-overlapping parts, segmentation is a technique for isolating lesions from the normal tissues and background. Global thresholding, local thresholding, and traditional approaches based on image histograms are all used in this breast lesions segmentation process. This work uses the metaheuristics GTORBL and GTO to homogeneity parameter thresholds, resulting in pixel-based area expansion strategies.

#### Maximization of Kapur entropy

The bilevel thresholding^81^ method divides an image into background and foreground. This is not applicable to a complicated image having multiple objects. Therefore, the multilevel thresholding techniques^19,82^ replaces the bilevel thresholding and achieves the optimal threshold values for image segmentation. Kapur’s entropy is a practical statistic for multilevel thresholding segmentation. The image is split into various classes, and the entropy determines whether the category is uniform. The calculating technique for Kapur’s entropy is straightforward, and it is simple to implement. It has a great degree of consistency and can be processed quickly. In addition, it provides a high level of accuracy in segmentation. Image segmentation utilising multilayer thresholding and Kapur entropy minimization employs metaheuristics such as the Moth-Flame optimization (MFO) method and the Crow Search Algorithm (CSA).

A rapid recursive segmentation technique based on Kapur’s entropy was utilised by Upadhyay and Chhabra^83^. Based on testing results, the proposed technique can boost the speed of calculation in five-level thresholding by 70 times in comparison to the traditional Kapur’s method. Using the crow search method, Kiani et al.^84^ presented Kapur’s entropy optimum multilevel segmentation of image. To attain ideal threshold values, Kapur’s entropy is utilised as a fitness function, and the entropy is maximised using the CSA. The Kapur’s entropy is maximised using the crow search technique. For each candidate solution, a collection of random threshold values is first encoded. The excellence of the first solution is determined through the fitness function using Kapur’s entropy. Kapur’s entropy technique for multilevel thresholding is discussed as follows:

Consider the following image, which has a total of *G* grey levels in span 0 to $G-1$ and a sum of *N* pixels. *f*(*i*) is the frequency of the *i*th intensity level.

2 $$\begin{aligned} N=f(0) + f(1) +f(2) +\cdots f(G - 1). \end{aligned}$$

Eq. (3) gives the probability of the *i*th intensity level.

3 $$\begin{aligned} P_{i} =\dfrac{f_i}{N} \end{aligned}$$

Consider the following *M* thresholds: $th_1, th_2, \cdots th_M$, where $1\le M \le G-1$. Applying these thresholds, image is segmented into $M+1$ segments and notations are as follows: $Class(0) = \{ 0, 1, 2, \cdots , th_1-1\},\,\,\, Class(1) = \{th_1, th_1+1, \cdots , th_2-1\}$ and $Class(M+1) = \{th_{M}-1, th_{M-1}+1,\cdots , th_M$}. The method based on entropy is defined as given in Eq. (4). F ($th_1$, $th_2$, $\cdots$, $th_M$ ) = $E_0$ + $E_1$ + $\cdots$ + $E_M$, Where, $E_i$ is the entropy of *i*th class.

4 $$\eqalign{ {E_0} = & - \sum\limits_{i = 0}^{i = t{h_1} - 1} {\frac{{{P_i}}}{{{w_0}}}} \ln \frac{{{P_i}}}{{{w_0}}},\;{w_0} = \sum\limits_{i = 0}^{i = t{h_1} - 1} {{P_i}} \cr {E_1} = & - \sum\limits_{i = t{h_1}}^{i = t{h_2} - 1} {\frac{{{P_i}}}{{{w_1}}}} \ln \frac{{{P_i}}}{{{w_1}}},\;{w_1} = \sum\limits_{i = t{h_1}}^{i = t{h_2} - 1} {{P_i}} \cr {E_M} = & - \sum\limits_{i = t{h_M}}^{i = G - 1} {\frac{{{P_i}}}{{{w_M}}}} \ln \frac{{{P_i}}}{{{w_M}}},\;{w_M} = \sum\limits_{i = t{h_M}}^{i = G - 1} {{P_i}} \cr}$$

**Figure 3:**
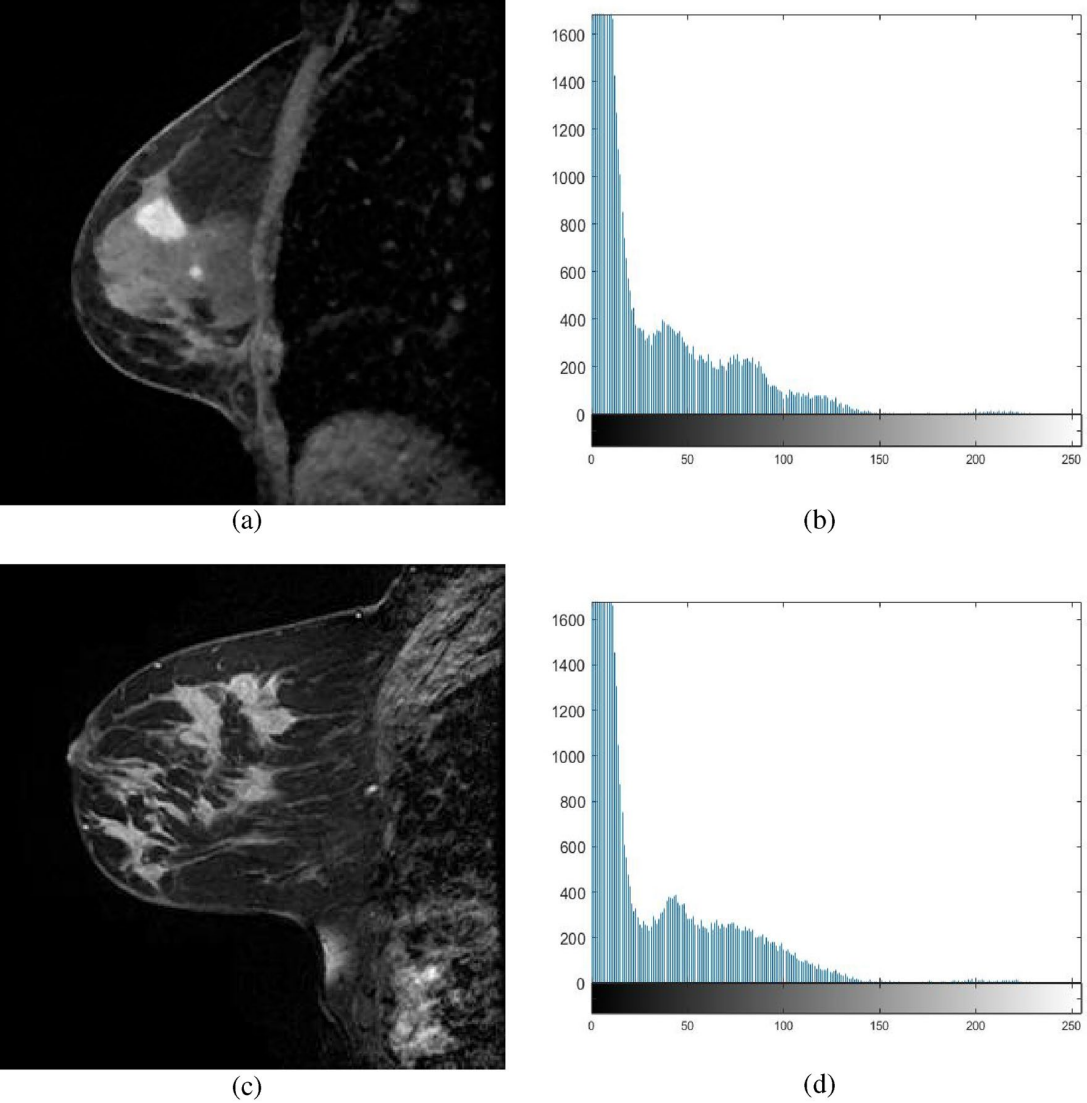
T2 weighted sagittal DCE-MRIs and histograms.

After the denoised and IIH corrected image are obtained, entropy maximization is carried out to find the suitable thresholds for segmentation. By expanding the number of homogeneous regions between them, entropy is maximized. The value of entropy is computed using the frequencies of pixel from histograms (Figs. 3b,d depicts the histograms of MR images shown respectively in Figures 3a,c). The entropy function, i.e. objective function is being maximized by GTORBL and GTO. As the gray levels of the image exist in [0, 255], the anticipated solutions, i.e. threshold values are integer values in the same range. The individual search agents represent the set of threshold values that are in the aforementioned range. The operators of GTORBL and GTO updates these individual solutions during the search process. The optimal threshold values are obtained after the termination of search algorithms and applied to segment the images. The GTO algorithm is discussed next.

#### Gorilla troops optimization

Five distinct operators are utilised in the GTO algorithm for optimization operations based on gorilla behaviours. Three different operators were used: migration to an unknown location to boost GTO investigation. The second operator improves the balance. The third operator considerably improves the GTO’s ability to find alternative optimization areas. All gorillas are defined as candidate solutions in the GTO algorithm, and the best candidate solution at every optimization operation stage is referred to as a silverback gorilla. Each of these three techniques is chosen using a standard procedure.

The technique of migration to an unknown site is chosen using a variable named *p*. When rand *p* is used, the first mechanism is chosen. If $rand \ge 0.5$, however, the gorilla-to-gorilla movement mechanism is chosen. If $rand < 0.5$, however, the technique of migration to a mentioned place is chosen. The first technique allows the algorithm to keep a close eye on the whole problem area, the second technique increases GTO effectiveness, and the third technique helps the GTO avoid local optimal locations. Eq. (5) is applied to simulate the three techniques applied in the exploration stage.

5 $$\begin{aligned} \begin{aligned} X^{g}(t + 1) ={\left\{ \begin{array}{ll} (X_{\max } - X_{\min }) \times r_1 + X_{\min }, \quad rand< p\\ (r_2 - C) \times X_r(t) + L \times H \quad \quad p \ge 0.5\\ X(i) - L \times (L \times (X(t) - X^{g}_r(t)) + r_3 \times (X(t)-X^{g}_r(t))), \quad rand < 0.5 \end{array}\right. } \end{aligned} \end{aligned}$$

In Eq. (5), $X^g(t + 1)$ is the location vector of gorilla in the next $(t+1)$ phase. *X*(*t*) is the present position vector of the gorilla. $r_3$, $r_2$, $r_1$, and $rand\in (0,1)$ is random values modified in every stage. *p* is a variable in the span $(0,\,1)$. The upper and lower boundaries of the parameters are denoted by $X_{\max }$ and $X_{\min }$, respectively. $X^g_r$ is one of the gorillas in the group who is chosen at random from the entire population, as well as $X_r$. *C*, *L*, and *H* are finally evaluated using Eqs. (6), (7) and (8) respectively.

6 $$\begin{aligned} C= & {} F \times (1-t) \end{aligned}$$

7 $$\begin{aligned} F= & {} cos (2 \times r_4) +1, \end{aligned}$$

8 $$\begin{aligned} L= & {} C \times l \end{aligned}$$

*F* is determined using Eq. (7). *cos* represents the cosine function, and $r_4\in (0,1)$ represents random values that are modified in every stage. Eq. (8) is applied to evaluate L, where l denotes random value in the span 1 and 1. To replicate silverback leadership, Eq. (8) is employed. Lack of experience in group leadership, the silverback gorilla may not be able to make the best decisions to find food the group in the real world; however, with enough experience, he achieves good stability in his leadership.

Also, in Eq. (5), H is computed using Eq. (9), while in Eq. (9), Z is computed using Eq. (10), where Z indicates random values in the span of $(-C,\, C)$.

9 $$\begin{aligned} H= & {} Z \times X (t), \end{aligned}$$

10 $$\begin{aligned} Z= & {} [\,-C, C\,], \end{aligned}$$

A team formation is carried out at the last of the exploration stage. The value of every GX solution is manipulated at the last of the exploration stage, and if the value is $X^g (t) < X (t)$, the $X^g(t)$ is utilized as the *X*(*t*). As a result, the best solution created during this stage is referred to as a silverback.

As indicated with the two processes utilised in the exploitation phase, the C value in Eq. (6) can be used for competition for senior females or follow the silverback. If $C \ge W$ then following the silverback technique is chosen while adult females’ competition is taken if $C < W$. The value *W* must be specified before to the optimization process.

The silverback is a young and healthy gorilla, and the other males in the team are likewise young and closely follow him. They also obey Silverback’s commands to go to different sites in quest of food supplies while remaining with him. Members might also have an impact on the overall movement of the group. When the $C \ge W$ is chosen, this technique is chosen. To imitate this behaviour, Eq. (11) is employed.

11 $$\begin{aligned} X^g(t + 1)= & {} L \times M \times (X(t) - X^s) + X(t), \end{aligned}$$

12 $$\begin{aligned} M= & {} \left( \left| \dfrac{1}{N} \sum \limits _{i=1}^{N} {X^g_i(t)}\right| ^{m} \right) ^{\dfrac{1}{m}} \end{aligned}$$

13 $$\begin{aligned} m= & {} 2^L, \end{aligned}$$

In Eq. (11), $X^{s}$ is the silverback position vector.

*N* is the total number of gorillas. If $C < W$, the second technique is chosen in the exploitation stage.

14 $$\begin{aligned} X^g (i)= & {} X^s - (X^s \times Q - X (t) \times Q) \times A, \end{aligned}$$

15 $$\begin{aligned} Q= & {} 2 \times r_5 - 1, \end{aligned}$$

16 $$\begin{aligned} A= & {} \beta \times E, \end{aligned}$$

17 $$\begin{aligned} E= & {} {\left\{ \begin{array}{ll} N_1, \quad rand \ge 0.5\\ N_2\quad rand < 0.5\\ \end{array}\right. } \end{aligned}$$

*Q* is thought to represent the force of impact. $r_5$ is a random variable ranging from 0 to 1 in Eq. (15). *E* is applied to simulate the influence of violence on the areas of results, and is a variable that should be provided a value by the optimization function. If $rand < 0.5$, E’s value will be equal to random values from the normal distribution and the problem’s dimensions, but if $rand \ge 0.5$, E will be equal to a random value from the normal distribution. An operation of group formation is performed at the end of the exploitation stage, in which the cost of all $X^g$ is evaluated, and if the cost of $X^g (t) < X (t)$, the $X^g(t)$ is employed as the *X*(*t*), and the best solution produced among the entire population is viewed as a silverback. The GTO algorithm is given in Algorithm 2.
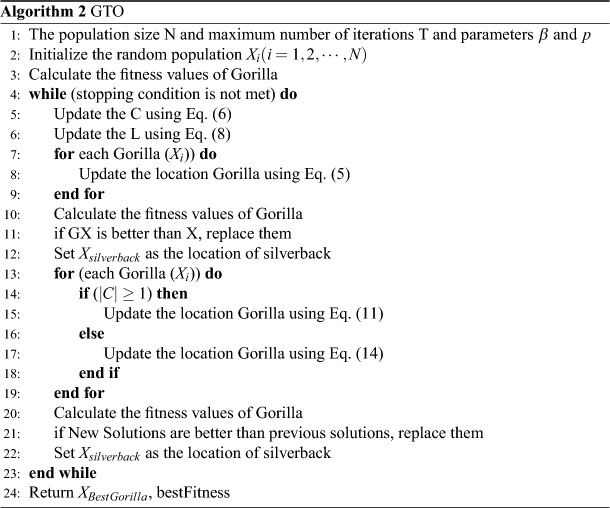


#### Rotational opposition based learning GTO

**Rotated-based explanation of OBL**

The primary idea underlying OBL^85–88^ is to consider an estimation and its inverse estimation at the same time in order to develop a candidate solution that is nearer to the global optimum. Rahnamayan et al.^89^ developed a Euclidean distance for optimal solution demonstration, which demonstrates intuitively why evaluating the reverse of a candidate solution is preferable to another random option. The key concepts of the OBL^87^ mechanism are as follows.

**Opposite number :** Let *z* $\epsilon$ [a, b] be a real number. The opposite number $\check{z}$ is calculated by

18 $$\begin{aligned} \check{z}= a + b - z \end{aligned}$$

**Opposite point :** Let $Z= (z_1,z_2,\cdots ,z_D)$ be a point in the D-dimensional search area, where $z_j \epsilon \, R_j = \, [a_j, b_j], j \epsilon [1,2,\cdots ,D]$ and $Z\, \epsilon \, S = \Pi _{j=1}^{D}\,R_j.$ The opposite point $\check{Z} = (\check{z_1},\check{z_2}, \cdots , \check{z_D})$

19 $$\begin{aligned} \check{z_j}= a_j + b_j - z_j \end{aligned}$$

It’s worth noting that the OBL method isn’t applied in every search generation. The defined probability variable, known as jumping rate ($J_r$)^90^ is a constant quantity in the range (0, 1) and is commonly established by actual experiences in various opposition-based optimization methods. The survey^91^ contains more information regarding OBL.

The centre of the established border in one-dimensional space reflects an opposite number to its original number in the OBL process. A 1-dimensional axis can, however, be placed in a 2-dimensional area to describe the opposing number in a different way.

**Figure 4:**
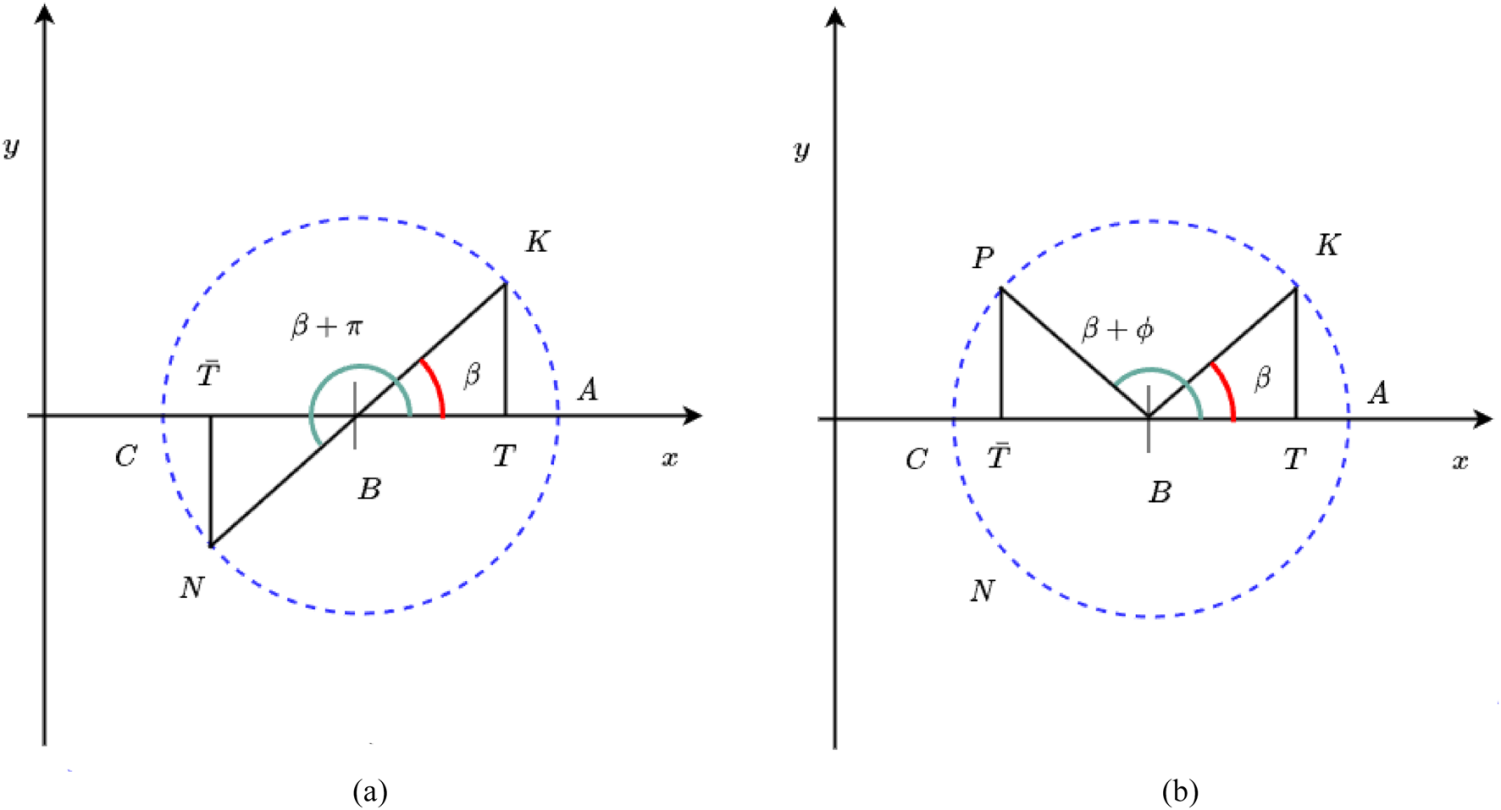
The geometric interpretation of opposite number (**a**), and rotation number (**b**) in 2D.

**Rotation-based learning (RBL) ** The OBL technique can be extended to an RBL^92^ technique by rotating any specified deflection angle, as shown in the analysis above. The geometric interpretation of opposite number and rotation number in 2D space are provided in Figure 4. A new point P is reached by rotating the K point $\phi ^\circ$ counterclockwise around the circle. As a result, $\angle PBA$ has an angle of $\beta$ + $\phi$. Point $Z^*$ is the projection of point N on the x axis, and its coordinate $T^*$ on the x axis is the rotation number of T. Suppose $u^*$ is the amount of the directed line segment $\vec {BT}$, which is produced by:

20 $$\begin{aligned} u^*=r \times (\cos (\beta + \phi ))=u \times \cos \phi - v \times \sin \phi \end{aligned}$$

$T^*$ can therefore be determined as follows:

21 $$\begin{aligned} T^*= (a + b)/2 + u^* \end{aligned}$$

It is obvious that the rotation number can find any point in the rotation area by rotating at various angles. Furthermore, the rotation number notion can be simply extended to higher dimensions. Let $T =(T_1,T_2,...,T_D)$ be a D-variable vector. $a =[ a_1,a_2,...,a_D]$ and $b =[ b_1,b_2,...,b_D]$ are the lower boundary and upper boundary of *T*, respectively, and $T_i \epsilon [a_i,\,b_i]$,   $i =1 ,2,...,D$. Therefore, the center point of rotation area $[a,\,b]^D$ is marked by $B =( B_1,B_2,...,B_D)$, and $B_i = (a_i+b_i) /2$. The radius vector is $R =( r_1,r_2,...,r_D)$, and $r_i = (b_i--a_i)/ 2$ . The vector $U =(u_1,u_2,...,u_D)$ indicates all the quantities of the directed line segment $\vec {B_iT_i}$, and $u_i$ is defined by

22 $$\begin{aligned} u_i=T_i - (a_i + b_i)/2 \end{aligned}$$

All the sizes of point $T_i$ to its corresponding correlation point $K_i$ on the circle are indicated by the vector $V =( v_1,v_2,...,v_D)$, and $v_i$ is defined by

23 $$\begin{aligned} v_i=\sqrt{(T_i - a_i)(b_i - T_i)} \end{aligned}$$

Allow the point *T* to rotate $\phi ^\circ$ rotating clockwise by the centre point B for each dimension, and the rotation point $T^* =( T^{*}_1,T^{*}_2,...,T^{*}_D)$ may be obtained, where the $T^*$ i is calculated by

24 $$\begin{aligned} T^{*}_{i}=(a_i + b_i)/2 + (u_i \times \cos \phi - v_i \times \sin \phi ) \end{aligned}$$

The suggested RBL mechanism, based on the preceding analysis, can rotate any degree in span $0^\circ$ and $360^\circ$ and explore any point in the search area. The RBL mechanism, for example, is equal to the OBL mechanism when the deflection angle is fixed at 180 degrees. The RBL process can be transformed into the quasi-oppositional learning (QOL) mechanism^89^ by carefully arranging the deflection angle so that the rotation points fall between the opposition point and the centre point M. As a result, the RBL mechanism can be thought of as a combination of the OBL and QOL mechanisms. Furthermore, RBL can be viewed as a random search if the deflection angle is adjusted arbitrarily between $(0^\circ , 360^\circ )$. As a result, the RBL mechanism is extremely adaptable when it comes to identifying potential promising solutions. The Gaussian distribution is simply utilized to compute the deflection angle in this study, and it is defined as where $\phi$ and $\phi _0$ denote the deflection angle and its basic number, respectively.

25 $$\begin{aligned} \beta = \beta _0 . N(1,\sigma ) \end{aligned}$$

The standard deviation of the Gaussian function P(.,.) is applied to regulate the fluctuation of the basic number and is denoted by $\sigma$. An starting value $\phi _0$ should be established first before calculating the parameter. $\sigma$ and $\phi _0$ will be set to 0.25 and $180^\circ$, respectively, in the following trials. Then, $\phi$ will oscillate wildly around the $180^\circ$, primarily inside $[90^\circ ,\,\,270^\circ ]$. This is also the key to RBL’s capacity to overcome the disadvantages of the OBL technique. The RBL technique, like the OBL, can be included into a population-based algorithm. To put RBL into effect, first determine the rotation area by evaluating the minimum and maximum values of every dimension for all singles in the population area. The centre point of the rotation area, as well as the lower and upper bound vectors, should all be acquired. The rotation-based population is then constructed, consisting of rotation-based individuals calculated by (22)-(24). The proposed algorithm is given in Algorithm 4.
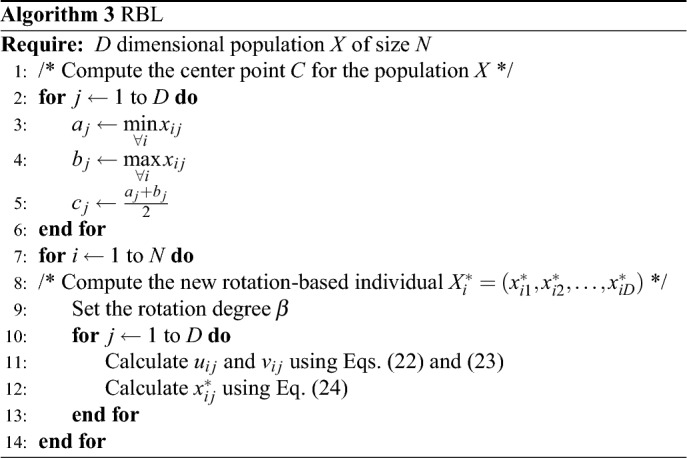

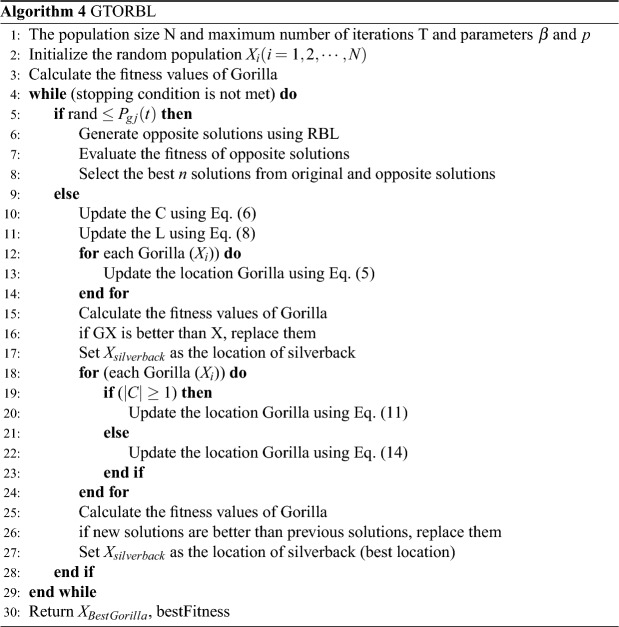


**Time & space complexity analysis** In the proposed GTORBL algorithm (Algorithm 4), steps 1-3 takes time respectively $O(\text {1})$, *O*(*N*.*D*), and *O*(*N*) where *D* is the dimension of the solutions. Step 6 takes *O*(*N*.*D*) as computation of RBL takes *O*(*N*.*D*) and Step 7 takes *O*(*N*). Step 8 takes $O(N.\log _2 N)$ if the Quick Sort algorithm is used for sorting. Steps 10-28 all together take *O*(*T*.*N*.*D*) as the time complexity of GTO is *O*(*T*.*N*.*D*). Putting all together, the time complexity of GTORBL algorithm is *O*(*T*.*N*.*D*). Therefore, the time complexities of both GTO and GTORBL algorithms are the same. The space complexity of GTO algorithm is *O*(*N*.*D*) as it store the *D* dimensional *N* population in a $N\times D$ array and as there is no requirement of additional memory having size greater than $N\times D$ during the run time. On the other hand, RBL computation takes *O*(*N*.*D*) space and therefore, space complexity of GTORBL is also *O*(*N*.*D*). Hence, the space complexities of both GTO and GTORBL algorithms are also the same.

### Post-processing

The lesions are eventually extracted from the segmented MR images utilizing GTORBL and GTO-based algorithms in this step. Cluster centres are sorted in increasing order, and cluster numbers are allocated to pixels.

The pixels in the lesional regions are of hyper-intense and hence, are assigned the highest labels which are selected to construct the lesioned image.

Region filling^93^ is used for all algorithms to enhance the segmentation outcomes. Using the pixel location of the detected lesions, the lesional regions are eventually overlaid with the actual MRI slice.

## Setup for experimentation

### Parameter settings

The competitive algorithms’ parameters are obtained from the studies in which they had been developed. The parameter settings are tabulated in Table 1. For all metaheuristic algorithms, the number of search agents is 30, and the maximum number of iterations for all the methods is set to 100. The configuration of the computational environment is provided in Table 2.

**Table 1:** The parameter settings of algorithms.

Algorithm	Parameter	Value
GTORBL	$\beta$	3
	*W*	0.8
	*p*	0.03
	$P_{gj}$	0.3
GTO	$\beta$	3
	*W*	0.8
	*p*	0.03
PSO	*W*	0.72984
	C_1_	1.49618
	C_2_	1.49618
SMA	*z*	0.03
MVO	$WEP_{\min }$	0.2
	$WEP_{\max }$	1
	$TDR_{\min }$	0.6
	$TDR_{\max }$	1
AOA	$\alpha$	5
	$\mu$	0.5
TSA	$P_{\min }$	1
	$P_{\max }$	4
IMRF	-	-
HMRF	EM iterations	5
	MAP iterations	5
CMRF	Potential	0.5

**Table 2:** PC Configuration.

Name	Configuration
CPU	Intel^®^ Core^™^ i5-8230U @ 2.30GHz.
RAM	8 GB
Operating system	Windows 10 home (64-bit)
Software	MATLAB 2021a

## Evaluation

For the assessment of algorithms’ performance, the following metrics are used: accuracy, sensitivity, specificity, $F_1$-score, FPR, GM, precision, and DSC ^43,44^. The definition of these metrics can be obtained from the study ^94^.

## Results & discussion

**Figure 5:**
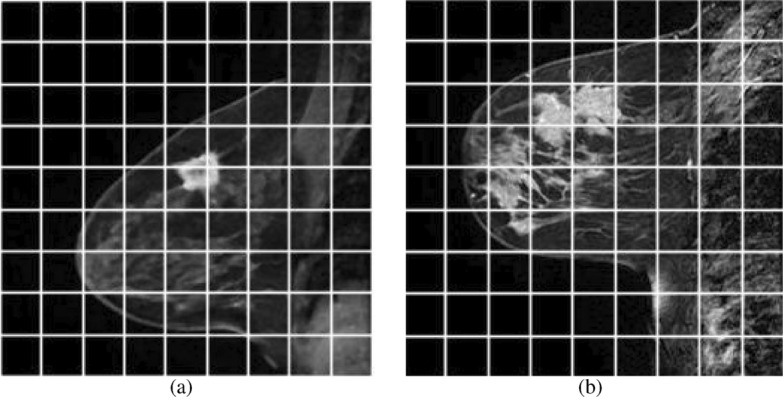
Original T2 weighted sagittal DCE-MRI.

**Figure 6:**
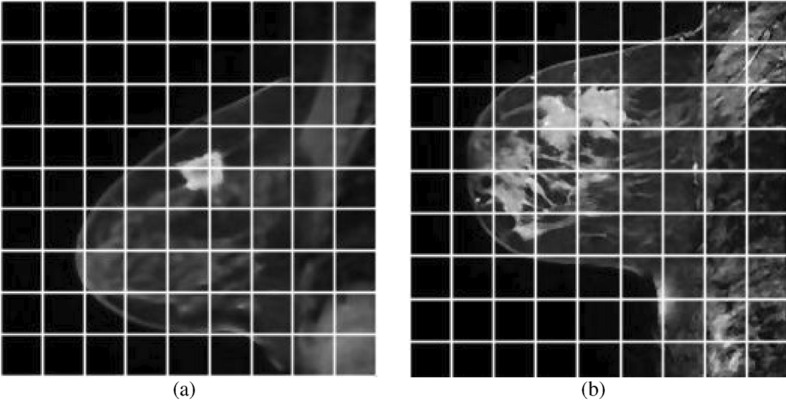
De-noised images of original T2 weighted sagittal DCE-MRI in Fig. 5.

**Figure 7:**
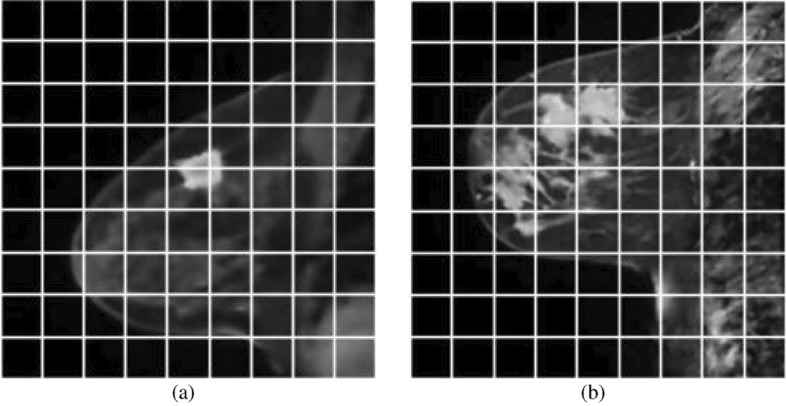
IIH corrected Images of denoised images in Fig. 6.

This study develops the lesion segmentation approaches for breast MRI. The MR slices’ noise and IIHs make difficulties for segmentation. For that reason, an ADF is used to detect noise from MR images, and IIHs correction is performed in the preprocessing step. The denoised images of MRIs in Fig. 5a,b are provided respectively in Fig. 6a,b. The IIH corrected images after denoising are provided in Fig. 7a,b. Because the GTORBL and GTO approaches start with a randomly initialised population, the same tests are repeated 10 times for a single image. The quantitative consequences of the mean value and standard deviation value of performance evaluation indicators are analysed using $10 \times 100$ findings. The TSA, PSO, AOA, MVO, SMA, HMRF, IMRF, and CMRF procedures are reviewed and compared to the novel methodologies.

### Quantitative results

Table 3 presents the quantitative outcomes of the approaches devised, other metaheuristics, and other three existing methods. The boxplot of comprehensive performance of all the approaches over 100 MR images are shown in Figures 8, 9, 10, 11, 12, 13, 14, 15.

From Table 3, the mean accuracies obtained using the GTORBL and GTO are higher than that achieved by other methods. GTORBL and GTO-based segmentation have respectively 99.31% and 98.13% accuracies, higher than all other compared methods. The mean sensitivity and specificity of the suggested approaches are also higher than those of all techniques that were compared.

Precision is another crucial metric for lesion detection. GTORBL and GTO have respectively mean precision of 86.33% and 83.91%, whereas AOA, PSO, TSA, SMA, MVO, HMRF, IMRF, and CMRF have mean precision of $82.11\%$, $78.96\%$, $80.92\%$, $82.74\%$, $79.79\%$, $79.49\%$, $78.83\%$, and $75.01\%$ which are lower than GTORBL and GTO.

The proposed GTORBL and GTO-based methods, have respectively mean GM of 97.41% and 93.82%, higher than that of all other compared methods.

The FPR, is the proportion of wrongly labelled negative samples to all negative samples. The mean FPR of the GTORBL and GTO is lower than that of the other examined methods. The lower the FPR value, the fewer negative samples in the segmented image there are.

DSC is another crucial metric for lesion detection. The greater the overlap between segmented lesions and Ground Truth (GT), the higher the DSC value. The mean DSC of AOA, PSO, TSA, MVO, SMA, HMRF, IMRF, and CMRF are respectively $83.69\%$, $83.54\%$, $83.05\%$, $83.86\%$, $85.87\%$, $82.04\%$, $82.97\%$, and $81.51\%$ which are very lower than that of GTORBL and GTO.

It is worth noting that GTORBL achieves higher values for all the metrics than GTO, which indicates that GTORBL quantitatively performs better than GTO.

**Figure 8:**
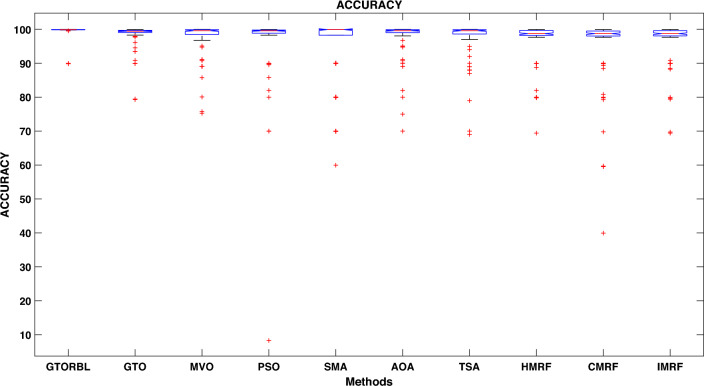
Approaches versus accuracy.

A boxplot graph depicts the distribution of data in the details. When compared to a density plot, however, boxplots can appear rudimentary. The suggested GTORBL is having a higher median classification accuracy than the other nine current techniques, as shown in Fig. 8.

The lowest difference and highest value are quite modest because of the compactness of the accuracy results.

**Figure 9:**
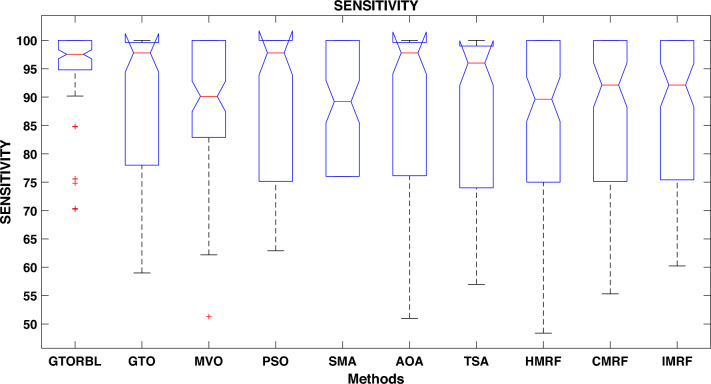
Approaches versus sensitivity.

GTORBL is also having a higher median sensitivity than the others shown in Fig. 9.

**Figure 10:**
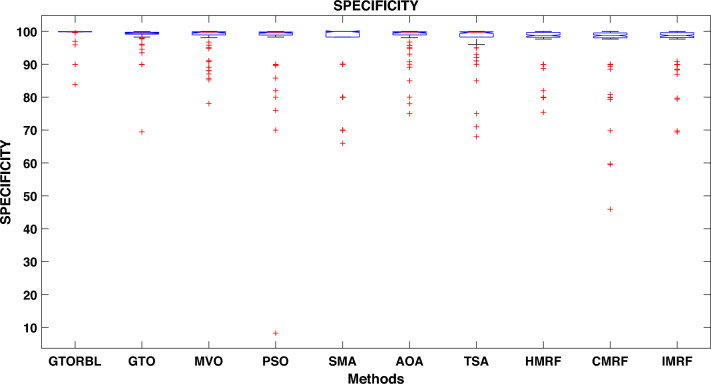
Approaches versus specificity.

**Figure 11:**
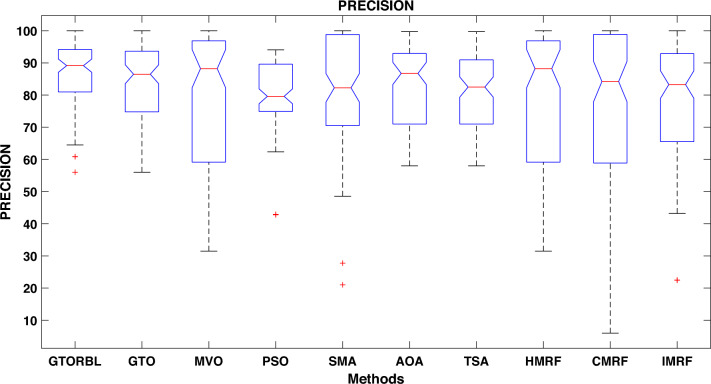
Approaches versus precision.

**Figure 12:**
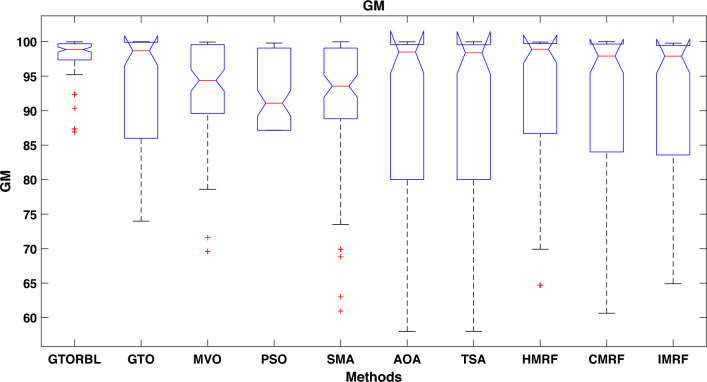
Approaches versus GM.

**Figure 13:**
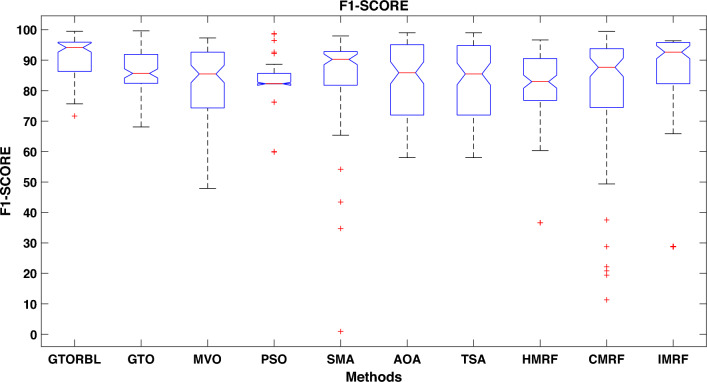
Approaches versus. $F_1$-score.

**Figure 14:**
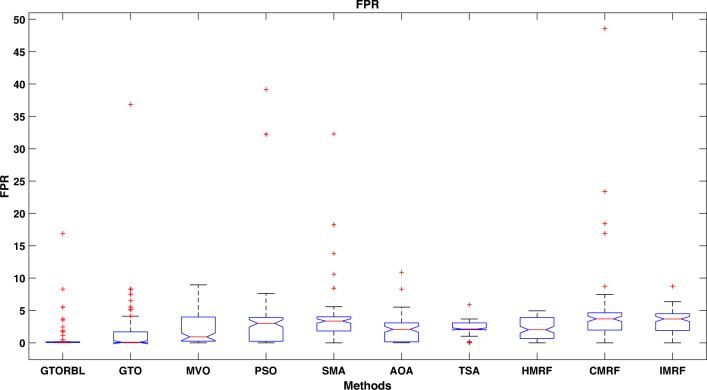
Approaches versus FPR.

**Figure 15:**
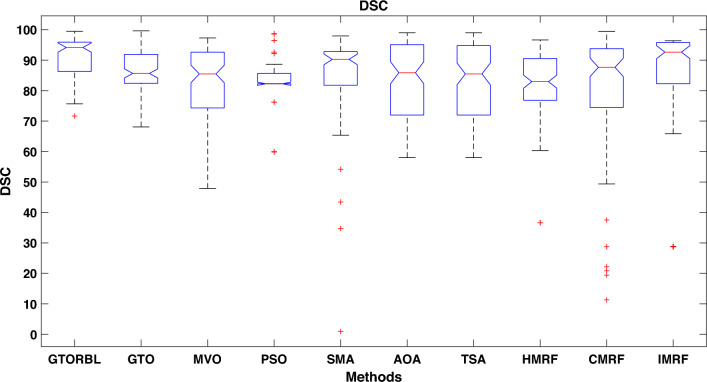
Approaches versus DSC.

As shown in Fig. 10, the recommended GTORBL and GTO have a higher median specificity of categorisation than the other techniques. Figure 11 shows that GTORBL has a greater median precision value than the other techniques. GTORBL has a higher median GM, $F_1$-score, FPR, and DSC than others as noticed from Figs. 12, 13, 14, and 15 respectively.

**Table 3:** Performance evaluation values (in %) for proposed methods GTORBL and GTO existing methods MVO, PSO, SMA, AOA, TSA, CMRF, IMRF and HMRF.

Performance Matrix	GTORBL	GTO	MVO	PSO	SMA	AOA	TSA	HMRF	CMRF	IMRF
Accuracy	**99.31 ** (0.0238)	98.13 (0.0372)	97.91 (0.0471)	96.86 (0.1049)	96.25 (0.0832)	97.89 (0.0506)	97.63 (0.0549)	97.59 (0.0498)	95.72 (0.0934)	96.83 (0.0583)
Sensitivity	**95.45** (0.0688)	90.96 (0.1171)	90.32 (0.1031)	90.94 (0.1183)	87.88 (0.1165)	89.91 (0.1325)	88.98 (0.1331)	85.59 (0.1487)	86.76 (0.1432)	87.26 (0.1392)
Specificity	**99.37** (0.0235)	98.28 (0.0382)	97.98 (0.0411)	96.92 (0.1035)	96.31 (0.0807)	97.99 (0.0456)	97.68 (0.0544)	97.65 (0.0466)	95.78 (0.0899)	96.91 (0.0567)
Precision	**86.33** (0.1059)	83.91 (0.1065)	79.79 (0.1805)	78.96 (0.1224)	82.74 (0.1635)	82.11 (0.1223)	80.92 (0.1126)	79.49 (0.1796)	75.01 (0.2919)	78.83 (0.1684)
GM	**97.41** (0.0341)	93.82 (0.0711)	92.58 (0.0762)	92.71 (0.0573)	91.92 (0.0912)	91.95 (0.1157)	91.49 (0.1183)	92.63 (0.1091)	91.72 (0.1014)	92.21 (0.0988)
F_1_-score	**91.54** (0.0635)	87.04 (0.0628)	83.86 (0.0966)	83.54 (0.0811)	85.87 (0.1357)	83.69 (0.1236)	83.05 (0.1214)	82.04 (0.1107)	81.51 (0.1875)	82.97 (0.2112)
FPR	**0.63** (0.0208)	1.62 (0.0417)	2.32 (0.0237)	3.08 (0.0589)	3.69 (0.0421)	2.01 (0.0181)	2.32 (0.0098)	2.35 (0.0169)	4.22 (0.0559)	3.10 (0.0169)
DSC	**91.54** (0.0635)	87.04 (0.0628)	83.86 (0.0966)	83.54 (0.0811)	85.87 (0.1357)	83.69 (0.1236)	83.05 (0.1214)	82.04 (0.1107)	81.51 (0.1875)	82.97 (0.2112)

The bold-faced results indicate a better.

#### Robustness analysis

The robustness of the proposed approaches is also significant. It is computed over numerous independent runs using the standard deviation (std. dev.) of performance metric, and a smaller std. dev. indicates higher robustness. Because the std. dev. values in Table 3 are minimal while comparing with eight current approaches, breast abnormalities can be detected robustly using the suggested segmentation approaches.

#### Statistical analysis: ANOVA followed by Tukey HSD

To validate the segmentation performance, a statistical analysis based on DSC was conducted using one-way ANOVA followed by a post-hoc test Tukey HSD.

Table 4 provides the results this test and It is noted that the *p-value* of the ANOVA test is $3.45E-07$ less than the significance level ($\alpha$) 0.05. Following the ANOVA test, the Tukey HSD statistical test is used to compare the GTORBL and GTO-based approaches to the other methods, and the findings are presented in Table 5 which indicates that GTORBL statistically outperforms the GTO and others with $\alpha =0.05$.

**Table 4:** ANOVA Test based on DSC.

Source of variance	SS	df	MS	F	*p* value	F-crit.
Between groups	7937.329	9	881.9255	5.3635	3.45E−07	1.8893
Within groups	162786.5	990	164.4308			
Total	170723.8	999

**Table 5:** Tukey HSD Test based on DSC.

Sl. No.	Pair	Comparison with *T*	Result (Significant at $\alpha$=0.05)
1	GTORBL versus GTO	$4.5 > 4.1876$	Yes
2	GTORBL versus PSO	$8 > 4.1876$	Yes
3	GTORBL versus SMA	$5.67 > 4.1876$	Yes
4	GTORBL versus MVO	$7.68 > 4.1876$	Yes
5	GTORBL versus AOA	$7.85> 4.1876$	Yes
6	GTORBL versus TSA	$8.49 > 4.1876$	Yes
7	GTORBL versus CMRF	$10.03 > 4.1876$	Yes
8	GTORBL versus HMRF	$9.5 > 4.1876$	Yes
9	GTORBL versus IMRF	$8.57 > 4.1876$	Yes

#### Statistical analysis: Wilcoxon signed rank test

A non-parametric test is used to assess the algorithms’ performance. To compare the devised GTORBL and GTO methods to the other methods pair-wise, the Wilcoxon Signed Rank Test (WSRT) is used. Tables 6, 7, 8, 9, 10, 11, 12, 13 describe the test results based on accuracy, sensitivity, specificity, precision, GM, $F_1$-score, FPR, and DSC, respectively.

The significance level ($\alpha$) of this is 0.05. The $\alpha$ value is adjusted using Bonferroni correction technique^95^. The adjusted $\alpha$ for GTORBL and GTO are respectively ${\tilde{\alpha }}_1 = \alpha$ / (no. of comparisons) = 0.05 / 9 = 0.005 and ${\tilde{\alpha }}_2 =0.05$ / 8 = 0.00625. For accuracy, Table 6 shows that GTORBL performs statistically better than all other methods except MVO. Between GTORBL and MVO, there is no difference in statistical significance in accuracy. It has also been discovered that GTO outperforms all other methods except MVO. Between GTO and MVO, there is no statistically significant difference. GTORBL’s sensitivity is statistically better than all compared methods according to Table 7. It has also been discovered that GTO outperforms HMRF and CMRF statistically. GTO and PSO, GTO and SMA, GTO and MVO, GTO and AOA, GTO and TSA, GTO and IMRF have no statistically significant differences. According to Table 8, GTORBL’s specificity is also statistically superior to others. It has also been discovered that GTO statistically outperforms TSA, PSO, AOA, SMA, HMRF, CMRF, and IMRF. Between GTO and MVO, there is no difference in statistical significance.

From Table 9, it is observed that

the precision of GTORBL is statistically superior to that of others. It has also been discovered that GTO outperforms TSA, SMA, AOA, HMRF, CMRF, and IMRF statistically. GTO and PSO, as well as GTO and MVO, have no statistically significant differences. According to Table 10, the GM of GTORBL is statistically better than others. It has also been discovered that GTO outperforms TSA, PSO, AOA, MVO, CMRF, and HMRF statistically. GTO and SMA, as well as GTO and IMRF, have no statistically significant differences for GM. The $F_1$-score and DSC of GTORBL are statistically better than others according to Tables 11 and 13 respectively. It has also been discovered that GTO outperforms TSA, PSO, AOA, SMA, and CMRF statistically. Between GTO and MVO, GTO and IMRF, and GTO and HMRF, there is no statistically significant difference. The FPR of GTORBL is significantly better than others, according to Table 12. It has also been discovered that GTO also outperforms others except GTORBL statistically.

**Table 6:** WSRT on accuracy.

Sl. No.	Pair	*p* value	Pair	*p* value
1	GTORBL versus GTO	0.000002	GTO versus MVO	0.0011553
2	GTORBL versus MVO	0.0000000	GTO versus PSO	0.00216579
3	GTORBL versus PSO	0.000000	GTO versus SMA	0.726968
4	GTORBL versus SMA	0.068373	GTO versus AOA	0.002369
5	GTORBL versus AOA	0.068373	GTO versus TSA	0.002369
6	GTORBL versus TSA	0.068373	GTO versus CMRF	0.002369
7	GTORBL versus CMRF	0.0000000	GTO versus IMRF	0.000047
8	GTORBL versus IMRF	0.0000000	GTO versus HMRF	0.001305
9	GTORBL versus HMRF	0.0000000

**Table 7:** WSRT on sensitivity.

Sl. No.	Pair	*p* value	Pair	*p* value
1	GTORBL versus GTO	0.000002	GTO versus MVO	0.0011553
2	GTORBL versus MVO	0.0000000	GTO versus PSO	0.00216579
3	GTORBL versus PSO	0.000000	GTO versus SMA	0.726968
4	GTORBL versus SMA	0.068373	GTO versus AOA	0.002369
5	GTORBL versus AOA	0.068373	GTO versus TSA	0.002369
6	GTORBL versus TSA	0.068373	GTO versus CMRF	0.002369
7	GTORBL versus CMRF	0.0000000	GTO versus IMRF	0.000047
8	GTORBL versus IMRF	0.0000000	GTO versus HMRF	0.001305
9	GTORBL versus HMRF	0.0000000

**Table 8:** WSRT on specificity.

Sl. No.	Pair	*p* value	Pair	*p* value
1	GTORBL versus GTO	0.000002	GTO versus MVO	0.0011553
2	GTORBL versus MVO	0.0000000	GTO versus PSO	0.00216579
3	GTORBL versus PSO	0.000000	GTO versus SMA	0.726968
4	GTORBL versus SMA	0.068373	GTO versus AOA	0.002369
5	GTORBL versus AOA	0.068373	GTO versus TSA	0.002369
6	GTORBL versus TSA	0.068373	GTO versus CMRF	0.002369
7	GTORBL versus CMRF	0.0000000	GTO versus IMRF	0.000047
8	GTORBL versus IMRF	0.0000000	GTO versus HMRF	0.001305
9	GTORBL versus HMRF	0.0000000

**Table 9:** WSRT on precision.

Sl. No.	Pair	*p* value	Pair	*p* value
1	GTORBL versus GTO	0.000002	GTO versus MVO	0.0011553
2	GTORBL versus MVO	0.0000000	GTO versus PSO	0.00216579
3	GTORBL versus PSO	0.000000	GTO versus SMA	0.726968
4	GTORBL versus SMA	0.068373	GTO versus AOA	0.002369
5	GTORBL versus AOA	0.068373	GTO versus TSA	0.002369
6	GTORBL versus TSA	0.068373	GTO versus CMRF	0.002369
7	GTORBL versus CMRF	0.0000000	GTO versus IMRF	0.000047
8	GTORBL versus IMRF	0.0000000	GTO versus HMRF	0.001305
9	GTORBL versus HMRF	0.0000000

**Table 10:** WSRT on GM.

Sl. No.	Pair	*p* value	Pair	*p* value
1	GTORBL versus GTO	0.000002	GTO versus MVO	0.0011553
2	GTORBL versus MVO	0.0000000	GTO versus PSO	0.00216579
3	GTORBL versus PSO	0.000000	GTO versus SMA	0.726968
4	GTORBL versus SMA	0.068373	GTO versus AOA	0.002369
5	GTORBL versus AOA	0.068373	GTO versus TSA	0.002369
6	GTORBL versus TSA	0.068373	GTO versus CMRF	0.002369
7	GTORBL versus CMRF	0.0000000	GTO versus IMRF	0.000047
8	GTORBL versus IMRF	0.0000000	GTO versus HMRF	0.001305
9	GTORBL versus HMRF	0.0000000

**Table 11:** WSRT on $F_1$-score.

Sl. No.	Pair	*p* value	Pair	*p* value
1	GTORBL versus GTO	0.000002	GTO versus MVO	0.0011553
2	GTORBL versus MVO	0.0000000	GTO versus PSO	0.00216579
3	GTORBL versus PSO	0.000000	GTO versus SMA	0.726968
4	GTORBL versus SMA	0.068373	GTO versus AOA	0.002369
5	GTORBL versus AOA	0.068373	GTO versus TSA	0.002369
6	GTORBL versus TSA	0.068373	GTO versus CMRF	0.002369
7	GTORBL versus CMRF	0.0000000	GTO versus IMRF	0.000047
8	GTORBL versus IMRF	0.0000000	GTO versus HMRF	0.001305
9	GTORBL versus HMRF	0.0000000

**Table 12:** WSRT on FPR.

Sl. No.	Pair	*p* value	Pair	*p* value
1	GTORBL versus GTO	0.000002	GTO versus MVO	0.0011553
2	GTORBL versus MVO	0.0000000	GTO versus PSO	0.00216579
3	GTORBL versus PSO	0.000000	GTO versus SMA	0.726968
4	GTORBL versus SMA	0.068373	GTO versus AOA	0.002369
5	GTORBL versus AOA	0.068373	GTO versus TSA	0.002369
6	GTORBL versus TSA	0.068373	GTO versus CMRF	0.002369
7	GTORBL versus CMRF	0.0000000	GTO versus IMRF	0.000047
8	GTORBL versus IMRF	0.0000000	GTO versus HMRF	0.001305
9	GTORBL versus HMRF	0.0000000

**Table 13:** WSRT on DSC.

Sl. No.	Pair	*p* value	Pair	*p* value
1	GTORBL versus GTO	0.000002	GTO versus MVO	0.0011553
2	GTORBL versus MVO	0.0000000	GTO versus PSO	0.00216579
3	GTORBL versus PSO	0.000000	GTO versus SMA	0.726968
4	GTORBL versus SMA	0.068373	GTO versus AOA	0.002369
5	GTORBL versus AOA	0.068373	GTO versus TSA	0.002369
6	GTORBL versus TSA	0.068373	GTO versus CMRF	0.002369
7	GTORBL versus CMRF	0.0000000	GTO versus IMRF	0.000047
8	GTORBL versus IMRF	0.0000000	GTO versus HMRF	0.001305
9	GTORBL versus HMRF	0.0000000		

#### Multi-criteria decision analysis

Multi-Criteria Decision Analysis (MCDA)^96^ is carried out using a well-known Technique for Order of Preference by Similarity to Ideal Solution (TOPSIS) method and we have adopted this philosophy from the study^44,97^. All the eight metrics are used as criteria in TOPSIS. The higher values of accuracy, sensitivity, specificity, precision, $F_1$-score, GM, FPR, and DSC indicates better. FPR contradicts with other metrics, i.e. criteria since lower FPR numbers indicate better. The ranks acquired using the TOPSIS approach are described in Table 14. According to Table 14, the GTORBL technique gets the highest ranking. The GTORBL method is then used after the GTO. It’s also worth mentioning that CMRF achieves the last rank.

**Table 14:** TOPSIS Rank.

Methods	Rank
GTORBL	1
GTO	2
AOA	3
MVO	4
PSO	5
SMA	6
TSA	7
HMRF	8
IMRF	9
CMRF	10

### Visual results

**Figure 16:**
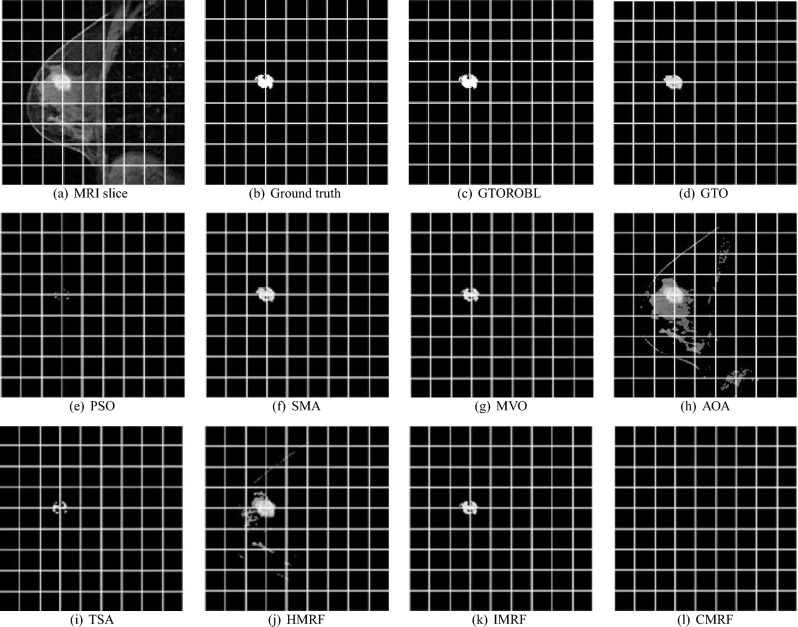
For patient-1 (**a**) MRI slice, (**b**) ground truth, and (**c**–**l**) GTORBL, GTO, PSO, SMA, MVO, AOA, TSA, HMRF, IMRF, and CMRF respectively.

**Figure 17:**
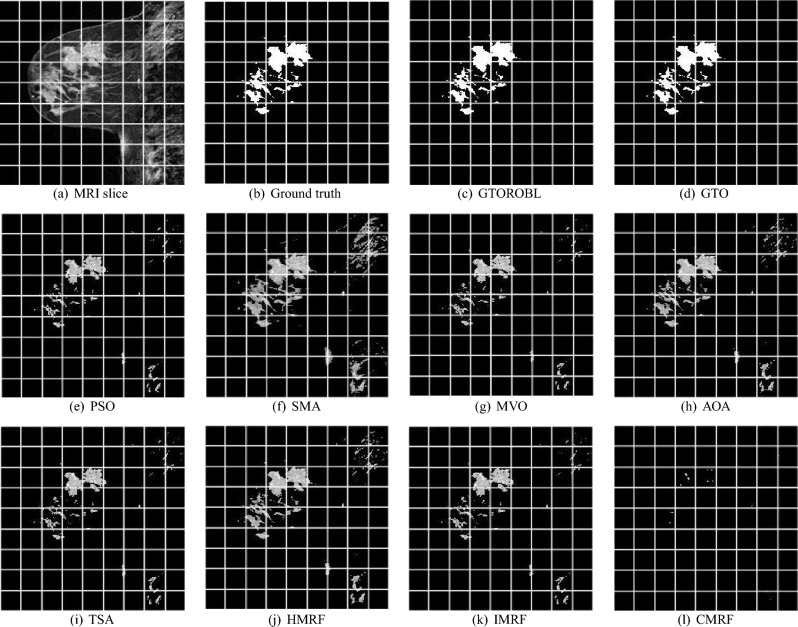
For patient-2 (**a**) MRI slice, (**b**) ground truth, and (**c**–**l**) GTORBL, GTO, PSO, SMA, MVO, AOA, TSA, HMRF, IMRF, and CMRF respectively.

**Figure 18:**
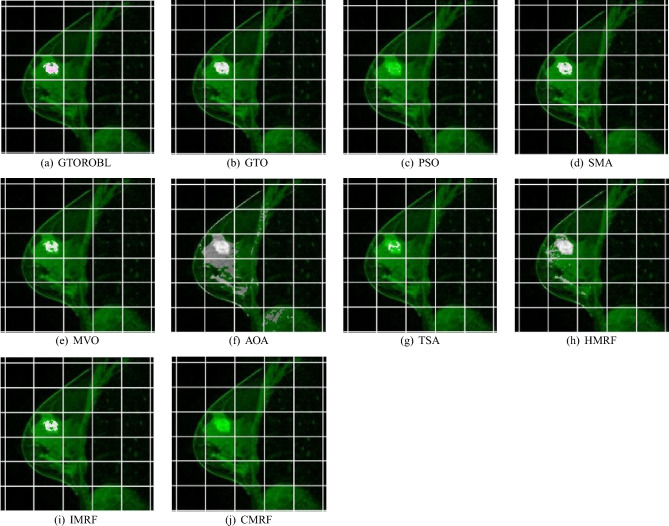
Localized lesions (bright colored spot) in MR images for patient-1 (**a**) GTORBL, (**b**) GTO, (**c**) PSO, (**d**) SMA, (**e**) MVO, (**f**) AOA, (**g**) TSA, (**h**) HMRF (**i**) IMRF, and (**j**) CMRF.

**Figure 19:**
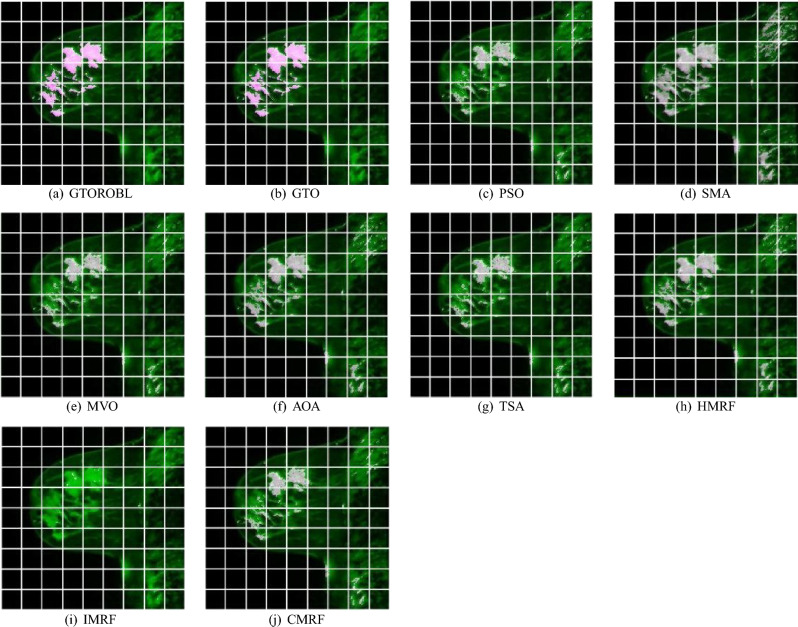
Localized lesions (bright colored spot) in MR images for patient-2 (**a**) GTORBL, (**b**) GTO, (**c**) PSO, (**d**) SMA, (**e**) MVO, (**f**) AOA, (**g**) TSA, (**h**) HMRF, (**i**) IMRF, and (**j**) CMRF.

A total of 100 MRIs of 20 patients are used to validate the proposed segmentation algorithms. Only 2 images of 2 different patients are shown due to space limits, out of 100 test findings. Figures 16 and  17 for respectively patient-1 and patient-2, demonstrate segmented lesions in breast using different methodologies. Figures 18 & 19 show images with a localised lesion, respectively.

When comparing the segmented image in Fig. 16a obtained from GTORBL with the GT in Fig. 16b, it has been observed that GTORBL almost perfectly segments lesions. In the segmented image obtained from GTO, lesions are almost entirely segmented, as shown in Fig. 16d. GTORBL and GTO are both effective at detecting lesions in images. A few lesions are not segmented by PSO, as shown in the segmented image in Fig. 16e generated by PSO. The PSO method is unable to detect lesions while comparing with the GTs.

The segmented image in Fig. 16f obtained from SMA demonstrates that a few lesions are not segmented. The SMA-based technique does not detect the lesions well when compared to the GTs.

The segmented images in Fig. 16g,h obtained by MVO and AOA respectively show that they are also unable to segment a small number of lesions. The MVO and AOA are ineffective in detection of lesions.

TSA fails to segment most of the lesions, as observed in the segmented image in Fig. 16i. In the segmented image in Fig. 16j obtained from HMRF, it can also be shown that HMRF recognises some healthy or normal tissues as lesions and it can’t do well in lesion detecting. IMRF fails to segment most of the lesions, as observed in the segmented image in Fig. 16k. In Fig. 16l, the CMRF segmented image, it is observed that CMRF fails to separate the large number of lesions.

For patient-2, when comparing the GT image in Fig. 17b with segmented image in Fig. 17c obtained from GTORBL, it is observed that GTORBL segmented lesions practically correctly. It can also be shown in Fig. 17d that GTO has virtually fully segregated the lesion locations. By comparing the proposed methods to the GT, it is discovered that the developed methods in this current study perform better in the detection of lesions in the MR image. Other metaheuristics-based methods recognise certain healthy tissues as lesions and they do not perform well in lesion detection.

Figure 17j,k show how HMRF and IMRF segment different healthy tissues. These techniques do not perform well in lesion detection when compared to the GTs. Figure 17l indicates that CMRF can not segment the large amount of lesions, but rather the lesions at the legion’s edge. When compared to the GTs, this technique fails to detect the lesions in the MRI.

### Effects of pre-processing and post-processing

We conducted trials without pre-processing, without post-processing, and without both pre and post-processing to investigate the effects of pre-processing and post-processing in the suggested technique. Table 15 gives the mean results and TOPSIS ranks. The TOPSIS approach, like Sect. "Conclusion", has several criteria for accuracy, sensitivity, specificity, precision, GM, $F_1$-score, FPR, and DSC.

The GTORBL technique, according to Table 15, has the highest ranking. It is important to note that a method with only post-processing is not more effective than a method having both pre and post-processing.

These findings show that the proposed method’s pre-processing and post-processing both help to improve performance of the segmentation.

**Table 15:** Mean results (in %) and rank of proposal without pre-processing, without post-processing, without pre and post-processing, and proposal.

Method	Metrics								
	Acc.	Sens.	Spec.	Prec.	GM	F_1_-score	FPR	DSC	Rank
Without Pre-processing	97.81	94.23	97.86	85.86	96.34	90.08	2.14	90.08	3
Without Post-processing	98.71	95.13	98.76	86.45	97.51	93.07	1.24	93.07	2
Without Pre and Post-processing	97.21	93.03	97.26	84.15	95.34	89.13	2.74	89.13	4
Proposed Method (GTORBL)	99.10	95.83	99.21	87.05	98.24	94.89	0.79	94.89	1

### Convergence analysis

**Figure 20:**
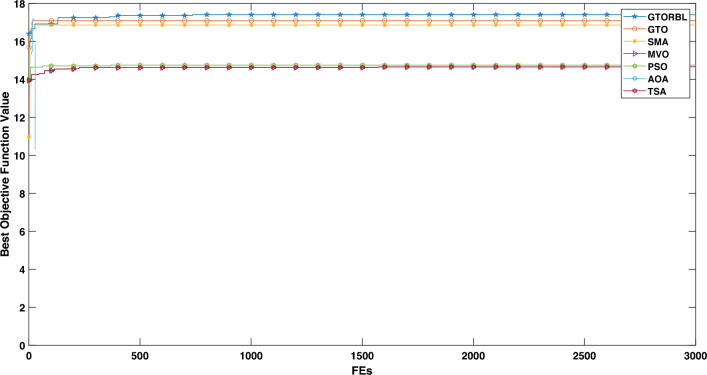
Convergence graph for MRI slice in Fig. 16a.

**Figure 21:**
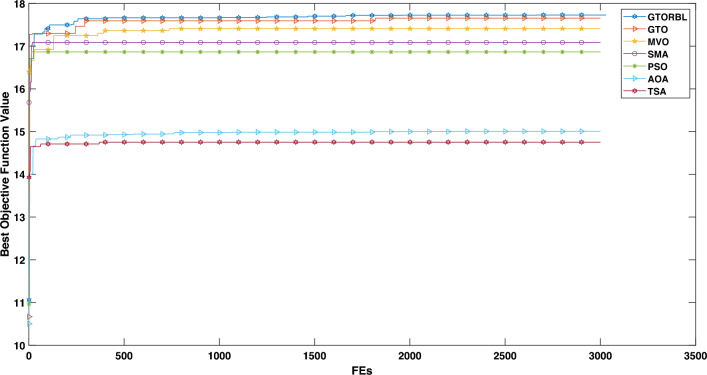
Convergence graph for MRI slice in Fig. 17a.

The convergence graphs are shown in Figs. 20 and  21 for the image in Figs. 16a and 17a, respectively. In graphs, the best entropy values, i.e. the best objective function values, are plotted against the Function Evaluations (FEs). The graph shows how far the search for the best solution has advanced. Graphs show that GTORBL converges better than other metaheuristics. The graph in Fig. 20 demonstrates that GTORBL exhibits convergence extremely close to the optimal output after around 100 FEs. GTORBL consistently outperforms GTO and PSO. According to the convergence graph in Figure 21, GTORBL converges very near to the best result after around 500 FEs, but GTO converges extremely close to the best result after about 1200 FEs. GTORBL converges quicker than GTO after 400 FEs. GTORBL regularly has higher values than PSO. PSO also converges prematurely in the local optima. The graphs show the GTORBL’s superior searching ability in the maximization of entropy.

### Computational complexity analysis

The average computational costs are 0.43 s and 1.24 s for respectively denoising and IIH correction.

**Table 16:** Mean computational cost.

Methods	GTORBL	GTO	AOA	PSO	TSA	MVO	SMA	HMRF	IMRF	CMRF
CPU time (s)	**3.3465**	3.7514	3.8827	3.9854	4.1025	4.0124	3.8546	4.5503	4.7499	4.8603

The bold-faced results indicate a better.

GTORBL requires a mean computational time cost of 3.3465 seconds, according to Table 16. The mean computational time for GTO is 3.7514 seconds. The mean computational time for PSO is 3.9854 seconds. SMA takes 3.8546 seconds to complete. The mean computational time for MVO is 4.0124 seconds. The mean computational time for AOA is 3.8827 seconds. TSA uses a mean of 4.1025 seconds of CPU time. The mean computational time for HMRF is 4.5503 seconds. The mean computational time for IMRF is 4.7499 seconds, whereas the mean computational time for CMRF is 4.8603 seconds. GTORBL is faster than all others, whereas GTO is slower than others.

In accordance with the aforementioned evaluation of both quantitative and qualitative (i.e., visual) results, both GTORBL and GTO perform better in the lesion segmentation of breast MRI than all the compared algorithms. GTORBL surpasses GTO in the segmentation of breast lesions. GTORBL outperforms all other algorithms in terms of segmentation consistency, but GTO also outperforms TSA, MVO, AOA, SMA, PSO, CMRF, IMRF, and HMRF. According to the results of the studies, both GTORBL and GTO are efficient and successful in breast lesion segmentation in DCE-MRI.

### Discussion

In this paper, we present a breast tumor segmentation methodology. The proposed methodology makes use of Kapur’s entropy maximization characteristics and sagittal T2-weighted fat-suppressed DCE-MRI data. The approach is applied to the DCE-MRI dataset, and as shown in Table 3, it outperforms state-of-the-art techniques in terms of performance. We used ADF for denoising and a max filter-based approach to generate IIHs during the preprocessing stage. In GTORBL, the generation jumping phase produces better exploration in the search space than GTO. Because the search space is not diverse enough, basic GTO suffers from early convergence in local optima. So, we must enhance the GTO to produce solutions of higher caliber. As a result, in this paper, we have developed a modified GTO called GTORBL by incorporating RBL strategy. Entropy maximisation is used to choose the appropriate threshold values for segmentation after obtaining the denoised image. Entropy is intended to be maximised by increasing the number of homogenous sites between them and the entropy value is computed using the histograms’ pixel frequencies. The GTORBL algorithm maximises the objective function, which is the entropy function. The MR images are segmented using the obtained optimal threshold values and in the postprocessing step, the lesions are extracted from the segmented MR images. For all of the methods, we have employed region-filling to enhance the segmentation outcomes. The one-way ANOVA test and the post-hoc Tukey Honestly Significant Difference (HSD) test are the statistical analysis techniques utilised to analyse the data. A boxplot is a type of graph that displays the distribution of values in the specifics. Contrasted with a density plot, boxplots, on the other hand, may seem rudimentary. Figures 8, 9, 10, 11, 12, 13, 14, 15 demonstrate that the proposed GTORBL achieves a higher median classification result than the other nine current techniques. In addition, we employ multi-criteria decision-making to assess overall performance in accordance with the aforementioned criteria. The proposed methodology outperforms the nine examined methodologies in the experiments. The convergence graph reveals the superior entropy maximisation search performance of the GTORBL method. The suggested lesion segmentation method outperforms the compared methods, according to quantitative results using statistical analysis and multi-criteria decision analysis as well as qualitative results.

Although GTORBL surpasses other competing algorithms in this study, the performance of GTORBL can be enhanced with additional changes, such as better swarm initialization using chaos theory^98,99^. In this study, the generation jumping probability $P_{gj}$ is set by *trial-and-error* method and this limitation can be overcome by using deterministic or adaptive rules. The random search technique^100^ can also be used to solve this problem.

## Conclusion with prospective future works

The goals of this present study are the examination and development of a suitable approach for the lesion segmentation in fat-suppressed DCE of breast to assist radiologists and doctors not only in breast cancer diagnosis but also in treatment planning, and surgery. Two segmentation approaches are proposed in this article. GTO is employed in lesion segmentation in breast MRI in the first GTO-based approach. GTORBL is an upgraded variant of GTO that is used in place of GTO in the same framework as the first approach. These two approaches’ performance is compared to that of metaheuristics such as TSA, SMA, AOA, PSO, MVO, and the existing breast MRI segmentation methods such as HMRF, CMRF, and IMRF. The results show that the proposed methods surpass these aforementioned competitive methods in terms of specificity, accuracy, precision, sensitivity, $F_1$-score, GM, FPR, and DSC. Both GTORBL and GTO outperform other compared approaches in the segmentation of breast lesion in MRI, according to the experimental data and analyses utilising statistical approaches and MCDM. In DCE-MRI, GTORBL outperforms GTO in the segmentation of breast lesions. The methods given are effective and successful in detecting lesions in DCE-MRI of the breast.

This current study is limited to T2-W sagittal fat-suppressed DCE-MRI of breast. Therefore, the future investigation leads to apply the proposed method to other Breast MRI sequences such as T1-W, Ultrafast, and diffusion-weighted imaging (DWI). One limitation of proposed GTORBL is setting the value of generation jumping probability which is done empirically in this study and this can be overcome by means of some deterministic or adaptive rules in the future. We also intend to modify GTO in the future by including different OBL techniques. For multi-level thresholding, we employed Kapur entropy in this study. In the proposed methodologies, we want to study different entropies such as Rényi entropy, and Tsallis entropy. The developed approaches will also be used to segment the MRI of brain, kidney, liver, prostate, etc^101–106^.

## Supplementary Information

Supplementary Information 1.

## Data Availability

The datasets used and/or analysed during the current study are available in The Cancer Imaging Archive repository, https://wiki.cancerimagingarchive.net/pages/viewpage.action?pageId=3539225.
