# Supplementary material for: Identification of breast lesion through integrated study of gorilla troops optimization and rotation-based learning from MRI images
Source: Sci Rep. 2023 Jul 18;13:11577. doi: 10.1038/s41598-023-36300-3 (PMC10354050; doi:10.1038/s41598-023-36300-3)
Supplement: Supplementary file 1 — Supplementary Information 1. [file 41598_2023_36300_MOESM1_ESM.zip › Gorilla Troops Optimization MRI Images/Identification_of_Breast_Lesion_through_Integrated_Study_of_Gorilla_Troops_Optimization_and_Rotation_based_Learning_from_MRI_Images (3).pdf]

Identification of Breast Lesion through Integrated Study of Gorilla Troops Optimization and Rotation-based Learning from MRI Images

Tapas Si[1], Dipak Kumar Patra[2], Saurav Mallik[3,*], Anjan Bandyopadhyay[4], Achyuth Sarkar[5], and Hong Qin[6,*]

[1]Department of Computer Science & Engineering, University of Engineering & Management, Jaipur, GURUKUL, Sikar Road (NH-11), Udaipuria Mod, Jaipur, Rajasthan-303807, India
[2]Department of Computer Science, Hijli College, Kharagpur-721306, West Bengal, India
[3]Department of Environmental Health, Harvard T H Chan School of Public Health, Boston, MA, USA
[4]School of Computer Engineering, Kalinga Institute of Industrial Technology (KIIT), Bhubaneswar, Odisha, India
[5]Department of Computer Science & Engineering, National Institute of Technology Arunachal Pradesh, Arunachal Pradesh, India - 791113
[6]Department of Computer Science and Engineering, University of Tennessee at Chattanooga, Chattanooga, TN, USA.
[*]hong-qin@utc.edu, sauravmtech2@gmail.com, smallik@hsph.harvard.edu

ABSTRACT

Breast cancer has emerged as the most life-threatening disease among women around the world. Early detection and treatment of breast cancer are thought to reduce the need for surgery and boost the survival rate. The Magnetic Resonance Imaging (MRI) segmentation techniques for breast cancer diagnosis are investigated in this article. Kapur's entropy-based multilevel thresholding is used in this study to determine optimal values for breast DCE-MRI lesion segmentation using Gorilla Troops Optimization (GTO). An improved GTO, is developed by incorporating Rotational opposition based-learning (RBL) into GTO called (GTORBL) and applied it to the same problem. The proposed approaches are tested on 20 patients' T2 Weighted Sagittal (T2 WS) DCE-MRI 100 slices. The proposed approaches are compared with Tunicate Swarm Algorithm (TSA), Particle Swarm Optimization (PSO), Arithmetic Optimization Algorithm (AOA), Slime Mould Algorithm (SMA), Multi-verse Optimization (MVO), Hidden Markov Random Field (HMRF), Improved Markov Random Field (IMRF), and Conventional Markov Random Field (CMRF). The Dice Similarity Coefficient (DSC), sensitivity, and accuracy of the proposed GTO-based approach is achieved 87.04%, 90.96%, and 98.13% respectively. Another proposed GTORBL-based segmentation method achieves accuracy values of 99.31% , sensitivity of 95.45% , and DSC of 91.54%. The one-way ANOVA test followed by Tukey HSD and Wilcoxon Signed Rank Test are used to examine the results. Furthermore, Multi-Criteria Decision Making is used to evaluate overall performance focused on sensitivity, accuracy, false-positive rate, precision, specificity, F_1-score, Geometric-Mean, and DSC. According to both quantitative and qualitative findings, the proposed strategies outperform other compared methodologies.

Keyword: Breast DCE-MRI, Segmentation, Entropy, Multi-level thresholding, Gorilla Troops Optimization, Rotational Opposition-based learning.

Introduction

Breast cancer in women is one of the most frequent cancers worldwide, and it is the main reason why women die. In comparison to developed countries, mortality rates in low and middle-income countries are comparatively high. In 2020 breast cancer was being diagnosed in worldwide 2.3 million women, with 685,000 deaths owing to the disease. "As of the end of 2020, there were 7.8 million women alive who were diagnosed with breast cancer in the past 5 years, making it the world's most prevalent cancer"[95].

In high-income nations, early diagnosis and treatment have been demonstrated to be beneficial, and it should be done in low-income countries with minimal equipment. Breast cancer survival rates range from 40 percent in South Africa and 66 percent in India to more than 90 percent in high-income nations five years after diagnosis. In nations that have been successful in lowering it, the annual mortality rate from breast cancer has fallen by 2-4 percent. If global mortality rates decreased by 2.5 percent annually between 2020 and 2040, 2.5 million breast cancer deaths might be avoided.

Furthermore, in rural regions, a scarcity of medical specialists and experts exacerbates the difficulty of early and accurate breast cancer diagnosis, leading to a higher mortality rate.

Medical imaging testing is the most effective method for detecting breast cancer. Digital mammography, magnetic resonance imaging, microscopic slices, and infrared thermogram are some of the medical imaging modalities used for diagnosis. These modalities create images that have lowered mortality rates by 30–70%[84] as a technique of assisting radiologists and clinicians in recognising problems. Because image interpretation is operator-dependent and requires skill, information technology is required to speed up and improve the accuracy of diagnosis while also offering a second opinion to the expert[49]. Computer-Aided Diagnosis (CAD), which uses computerised characteristics extraction and classification algorithms, can be a very useful tool for physicians in spotting anomalies.

CAD systems based on breakthroughs in digital artificial intelligence, image processing, and pattern recognition have been developed with great effort. CAD systems are predicted to lower the cost of medical auxiliary modalities, eliminate operator dependency, and boost diagnosis rate[50,60,91] As a result, it may assist to prevent false positive rates, which can lead to ineffective treatment as well as psychological, physical, and financial expenses. It may also prevent erroneous negative readings, which could lead to treatment omissions and remissions. According to reports, detection sensitivity without CAD is around 80 percent, and sensitivity with it is around 90%[32]. According to the findings, CAD paired with mammography had 100 percent sensitivity in detecting malignancies appearing as microcalcifications and 86 percent sensitivity for other mammographic cancer presentations. As a result, CAD has emerged as the most effective area of research in medical imaging for improving diagnosis precision[24,93].

The use of a computer output to establish the location of questionable lesions is known as computer aided detection. Following that, radiologists are responsible for the diagnosis of abnormalities, as well as patient care. On the other hand, computer aided diagnosis takes a human or a machine's detection and produces an output that defines the nature of the lesion, as well as the likelihood of malignancy and any abnormalities[26].

We introduced a new segmentation approach based on GTO[1], which was first proposed in 2021. It is much more successful than its competitors at finding better solutions to complex optimization issues. As a result, we're eager to apply the GTO to breast DCE-MRI segmentation. Our main goal is to use the GTO to segment breast lesions in fat-suppressed DCE-MRI in order to produce better results than existing approaches. GTO is employed in Kapur entropy maximization to segment the breast T2 WS DCE-MRI for lesion identification in the current study. The performance of the proposed method is compared with PSO[87], MVO[56], SMA[46], AOA[2], TSA[36], IMRF[98], HMRF[16], and CMRF[19] methods. Accuracy, sensitivity, specificity, geometric-mean, precision, false-positive rate, F_1-score, and DSC are all used to evaluate performance. The data are statistically analysed using two different statistical analysis methods: one-way ANOVA[5] test followed by post-hoc Tukey HSD[88] test and Wilcoxon Signed Rank Test[21] test. Furthermore, for overall performance evaluation, Multi-Criteria Decision Analysis (MCDA)[89] based on the aforementioned eight performance metric, i.e. criteria is employed, and this style of performance analysis is adapted from the study[63,79]. The proposed methods outperform the eight competing methods, according to the examination of the experimental findings employing the aforementioned three analysis.

0.1 Contributions of this article

The following is a summary of the contributions:

1. Two methods for breast lesion segmentation in fat-suppressed DCE-MRI utilising GTO has been devised. Before now, GTO had never been used for breast T2 WS DCE-MRI segmentation. In the first method, classical GTO is used. We developed an enhanced GTO with RBL and used it in the second method.

2. Experiments conducted with 100 slices of the breast DCE-MRI, and the findings were analysed using a set of pertinent metrics, including sensitivity, accuracy, precision, specificity, F_1-score, geometric-mean, DSC, False-Positive Rate (FPR), and convergence.

3. The metaheuristics such as MVO, PSO, SMA, AOA, and TSA, and three existing breast MRI segmentation methods such as CMRF, IMRF, and HMRF are compared with the proposed methods in this study.

4. Discussion of the outcomes using statistical and multi-criteria decision-making techniques.

5. Eight competitive methods are outperformed by the proposed approaches.

0.2 Organization of this article

The following is how the rest of the paper is organised: Section 1 explains the related works. The materials and procedures are discussed in Section 2. This section includes the GTO, proposed GTORBL algorithms, and proposed segmentation methods. The experimental setup is described at Section 3. Finally, Section 6 provides a conclusion as well as prospective future works.

1 Related Works

Segmentation of the Region of Interest (ROI), feature extraction, and classification are the three phases of any CAD system for diagnosis of breast cancer. Segmentation is a crucial step in the creation of a CAD system. The purpose of ROI segmentation is to distinguish the breasts from the rest of the body. Because of the importance of breast cancer segmentation, more studies have been conducted in recent years. As a result, this section summarises previous work on breast cancer segmentation using the DCE-MR image collection.

For breast segmentation in MRI, Patra et al.[62] produced a Grammatical Fireworks Algorithm (GFWA) technique. The GFWA clustering method is applied to segment MRI. The divided slices are separated from the lesions. The suggested technique's experimental findings (both quantitative and qualitative) are analysed to those of Grammatical Swarm and K-means clustering techniques, and the approach outperforms both. On 25 T2 WS DCE-MRI slices, this approach is being tested. Si and Mukhopadhyay[81] produced breast MRI segmentation for lesion diagnosis applying clustering through the Fireworks Algorithm (FWA). For lesion detection, a breast MRI segmentation technique based on improved hard-clustering with FWA has been produced. The segmentation approach is compared to PSO and K-means methods-based segmentation methods. Kar and Si[38] used clustering with MVO approach to generate breast MRI segmentation for lesion identification. For lesion detection, a modified clustering with MVO is devised. The K-means and PSO clustering are used to compare the results. Sun et al.[85] suggested a super voxel-based semi-supervised breast tumour segmentation algorithm. They employed cluster methods to detect the tumour location from MRI images and then used a supervised classifier to classify it.

Breast MRI segmentation focused on Student Psychological Based Optimization (SPBO) by thresholding employing Shannon entropy for lesion identification proposed by Patra et al.[63]. The MR slices are segmented using the SPBO method. After being retrieved from the segmented slices, the lesions are found in the original MR slices. On 300 T2 WS DCE-MRI slices that have been validated, this method is tested. The suggested segmentation approach has a 99.44% accuracy, a 96.84 percent sensitivity, and a DSC of 93.41%. Ibrahim et al.[33] suggested a segmentation approach focused on the chaotic salp swarm algorithm (CSSA). CSSA is a superpixel extraction algorithm based on chaotic maps and created utilising the fast-shift approach to extract superpixels with CSSA-optimized parameters. The DMRIR dataset used to check the output of this approach. Sayed et al.[82], proposed a CAD method that relies on automatic ROI segmentation. To obtain reliable results, the segmentation approach employs the advantages of four Bio-inspired swarming algorithms: PSO, Grey Wolf Optimization (GWO), Moth Flame Optimization (MFO), and Firefly algorithm (FA). Breast MRI segmentation focused on quasi opposition-based Sine Cosine Algorithm (SCAQOBL) by thresholding employing Shannon entropy for lesion identification proposed by Si et al.[77]. The MR slices are segmented using the SCAQOBL method. After being retrieved from the segmented slices, the lesions are found in the original MR slices. On 100 T2 WS DCE-MRI slices that have been validated, this method is tested. The suggested segmentation approach has a 99.11% accuracy, a 97.78 percent sensitivity, and a DSC of 95.42%. Breast MRI segmentation focused on opposition-based Chimp Optimization Algorithm (ChOAOBL) by thresholding employing Kapur entropy for lesion identification proposed by Si et al.[78]. The MR slices are segmented using the ChOAOBL method. On 200 T2 WS DCE-MRI slices that have been validated, this method is tested. The suggested segmentation approach has a 99.02% accuracy, a 95.73 percent sensitivity, and a DSC of 93.25%.

Azmi et al.[6] suggested breast cancer segmentation in MRI based on the Markov Random Field (MRF) approach. This approach employs a traditional MRF, in which the energy function is determined solely on levels of nearby pixels, which are allocated at random. By considering the spatial relationship between neighbouring pixels, the MRF model produces good results. The PIDER breast MRI dataset use this approach. The mean values for specificity, sensitivity, precision, and accuracy are 93.76 percent, 93.76 percent, 79.74 percent, and 93.10 percent, respectively.

Liu et al.[51] suggested a fully automated approach for bulk identification and segmentation of mammography slices. The approach uses a mass detection strategy that is split into two categories: (a) constructing a search template and (b) obtaining an image utilising template matching. Adaptive thresholding focused on the maximum entropy used to convert the image features into ROIs. The region-growing method used to eliminate mammography lumps from the backdrop. The technique was examined using 70 mammography slices from the Digital Database for Screening Mammography (DDSM). The suggested approach exhibited a sensitivity of 97.2 percent and 1.83 false positives. MRI-based breast tumor diagnosis created by Ha and Vahedi[29] utilizing the modified Deer Hunting Optimization algorithm, which is focused on a feature-related technique and an improved convolutional neural network. The goal of this project is to make classification easier by using the preprocessing stage. Using a Haralick texture and local binary pattern to extract features is also a nice idea. The dataset consists of 219 breast MR slices taken from 105 distinct breast cancer patients. As a consequence of the obtained data, the accuracy of this technique is 98.89 percent, suggesting its excellent potential and efficiency. Keyvanfard et al.[40] extracted texture, kinetic,

and shape characteristics from the lesion-containing segmented ROI and then used various classifiers to distinguish between malignant neural regression networks, including probabilistic neural network, multi-layer perceptron, generalized neural regression network, Support Vector Machine (SVM), and radial basis function network. The best results were merged to create a multi-classifier approach with a 90% accuracy rating. AlQoud et al.[4] implemented the technique of detecting breast cancer based on Gabor features and local binary pattern features. To diagnose breast cancer, the Artificial Neural Network (ANN) classification technique supervised here is used by classifying characteristics into normal tissues and abnormal tissues in the breast. To extract features from the ROI portion of the images, these methods use the adaptive k-means clustering technique. Here, the gray level co-occurrence matrix elements erase the pectoral muscle boundary. Finally, the irregular and regular breast tissues are identified using K-Nearest Neighbors (KNN) and SVM techniques. Dong et al.[25] developed a unique breast cancer detection framework. The feature results from the ROIs were then extracted using the improved vector field convolution features. Finally, the sorts of classes, malignant, and benign, classified using the random forest classification technique. The main disadvantage of random forest was its intricacy, as well as the fact that it took longer to generate the decision trees. Punitha et al.[71] offered an improved region-growing technique for automatic detection of breast masses. Texture information from the segmented images, such as the grey-level co-occurrence and run length matrix, retrieved and sent into the feed forward neural network as input. The performance of the recommended technique tested using a Gaussian filter to remove noise from 300 mammography slices. The specificity and sensitivity of the suggested approach were 97.8 percent and 98.1 percent, respectively. The performance was also 90.0 percent, according to the Jaccard index. Segmentation in mammography images and across-sensor comparison with customized and deep characteristics developed by Cardoso et al.[15]. The models in mammography images for mass segmentation are: (a) boundary computation and tailored features; (b) second and third models that are focused on deep learning features and combine smooth SVM and Conditional Random Field (CRF); and (c) a combined model based on tailored features and boundary computation. The cross-sensor performance loss is greater than 10.0 percent and the mammography slices are taken from DDSM-BCRP and INbreast.

Li et al.[44] developed the dense U-Net for automatically segmenting breast cancer in mammography images. For the segmentation of breast mass, this approach uses an automatic segmentation technique focused on deep learning. For mammography segmentation, this technique combines Attention Gates (AGs) with densely linked U-Net. Additionally, the DDSM database used to test this method, and the findings proved that dense U-Net coupled with AGs performed better than other methodologies. The technique yielded an F_1-score of 82.24 percent, a sensitivity of 77.89 percent, and accuracy of 78.38 percent. Zeiser et al.[99] developed a breast cancer diagnosis model based on U-Net. Data augmentation, image preprocessing, training, and testing are the four phases of the produced model. Following the acquisition of mammography slices from DDSM, image preprocessing utilised to improve contrast, obtain regions of interest, and remove extraneous information. Mirroring, scaling, and zooming of mammography slices were all done with data augmentation. Finally, the malignant and benign classifications were classified using the U-Net approach. The created model performed better in terms of accuracy, dice index, specificity, and sensitivity as evidenced by the experimental results. The location and orientation of the slices (lesion and non lesion areas) in the low-resolution mammography slice are not encoded by the U-Net model during detection. For early identification of breast cancer, Rampun et al.[70] proposed a breast area and pectoral muscle segmentation method. To detect the breast boundary, the active contour model utilised initially, followed by a postprocessing method to rectify the overstated border caused by artefacts. The pectoral muscle boundary detected and noisy edges were removed using a canny edge detection procedure and a preprocessing methodology. Then, through contour growth, five edge features used to hunt for the true border. In comparison to manual segmentation, the devised method achieved substantial results using dice similarity coefficients. Bora et al.[9] created a textural gradient approach for segmenting the pectoral muscle. The Hough transformation applied to estimate the pectoral edge on the expected textural gradient of the mammography image. The method used Euclidean distance regression and polynomial modelling to build smooth pectoral muscle curves and was resistant to overlapping and texturing fibro glandular tissues. According to the results of the trial, the approach performed better in terms of accuracy in pectoral muscle segmentation. Due to smaller size and decreased contrast, the developed approach failed to detect pectoral muscle from mammography images. Shen et al.[76] presented a new method for segmenting the pectoral muscle region from images automatically. Genetic algorithm, image preprocessing, polynomial curve fitting, and morphological selection are the four phases of the produced model. The genetic algorithm utilised to learn multilayer thresholds in this study, and the morphological selection technique employed to find the best contour of pectoral muscle areas based on morphological data. The efficiency of the created model tested using the micro Mammographic Image Analysis Society (MIAS), DDSM, and INBreast databases in this work. Because of the dense gland and mass border, the detection rate in the generated model reduced.

Nanayakkara et al.[61] offered a strategy for automatic mammography breast boundary segmentation. The skin line and breast segmentation estimated using a modified quick matching method and morphological operators. The recommended approach evaluated on 136 mammography images applying an alternating sequential filter to eliminate noise. The recommended

technique had a 99.2 percent ground truth sensitivity and a 99.0 percent segmentation accuracy. Desai et al.[22] suggested a method for detecting microcalcification in mammography by refining the segmentation approach. The approach uses a modified multiscale morphological gradient watershed segmentation in mammograms for identification of clustered microcalcifications. The efficiency of the suggested technique was assessed using 2 databases: MIAS and Nuclear Magnetic Resonance (NMR). Adaptive median filter used to reduce noise. The true positive rates found to be 95.4 percent and 94.0 percent for 2 databases.

Isa and Siong[34] developed automatic mass segmentation and identification in mammogram slices. The suggested approach splits mammography slices into two categories: (a) mass and their background pixels; and (b) Image enhancement increases the contrast of the image. The area of the breast mass discovered and segmented applying a region development focused on local statistical texture analysis. Total 322 mammography slices are tested to conduct this approach. The technique had a sensitivity of 94.59% and a FPR of 3.90 percent. Shrivastava et al.[72] developed an automated digital mammography segmentation approach based on a scattered region expanding and sliding window algorithm. To detect worrisome masses in mammography slices, the system employs a fully automated technique. The pectoral muscles were removed from the mammography using the sliding window approach. The mammography images taken from the MIAS, and the suggested approach had a 91.3 percent accuracy.

Using Gray-Level Co-Occurrence Matrix (GLCM) texture and fuzzy c-means characteristics, Saleck et al.[73] suggested cancer identification in mammogram slices. The FCM algorithm used in the suggested method as an automatic technique for bulk segmentation in mammography images. A median filter applied to eliminate noise from the 18 mammography images after they were downloaded from the MIAS database. The tumour was extracted from the ROI using the suggested method, which also included a fuzzy c-means algorithm; the FCM input was then validated using GLCM texture characteristics. The technique generated results with 96.4 percent specificity, 94.6 percent accuracy, and 86.2 percent sensitivity. Using an improved U-Net segmentation technique using mammography images, Hossain[28] presented microcalcification segmentation. With mammogram slices obtained from the DDSM, the suggested approach was trained, and noise was eliminated employing the Laplacian filter. The procedure was broken down into five steps: preprocessing, breast area segmentation, breast region extraction, breast region selection of positive patches, and breast region training of the segmentation area. The technique generated a Dice score of 97.80% and F-measure of 98.50%. The value of Jaccard index is 97.40 percent, while the proposed method's average accuracy is 98.20 percent.

The analysis of the literature reveals that tumour diagnosis is a tough and important undertaking. This paper proposes breast DCE-MRI segmentation based on entropy maximization using metaheuristics like GTO and GTORBL. The proposed approaches are explained in the next section.

2 Materials & Methodology

2.1 DCE-MRI dataset

A total of 100 images which includes T2-W fat-suppressed DCE-MRI slices of 20 patients are collected from publicly accessible "The Cancer Genome Atlas Breast Invasive Carcinoma Collection" (TCGA-BRCA)[18,47]. Each slice is having the size 256×256. The ground truths, regarded as the gold standard[52], are generated by expert radiologists. T2-weighted images are a useful tool for MRI breast mass evaluation. By carefully analysing T2-weighted images, it is possible to lower the false-positive rates and distinguish uncommon, well-circumscribed breast cancers from frequent benign breast masses. Fluid-sensitive T2-W MRI show edoema, haemorrhage, mucus, or cystic fluid in contrast to T1-W MRI, which emphasise anatomic detail. Using T2-W MRI, characterization of breast lesions can be performed more accurately than T1-W MRI[100]. Therefore, T2-W MRI are selected for breast lesion identification in this present study. In this current work, the MRI dataset is formulated by including breast DCE-MR images of 20 different women patients having different size of breast lesions from small to large, and from single lesion regions to multiple lesion regions. DCE-MRIs of benign and malignant breast lesions are included in this dataset.

2.2 Proposal

The following are the three steps of the proposal:

1. Pre-processing
2. Segmentation
3. Post-processing

Fig. 1 discussed flowchart of the proposed methods.

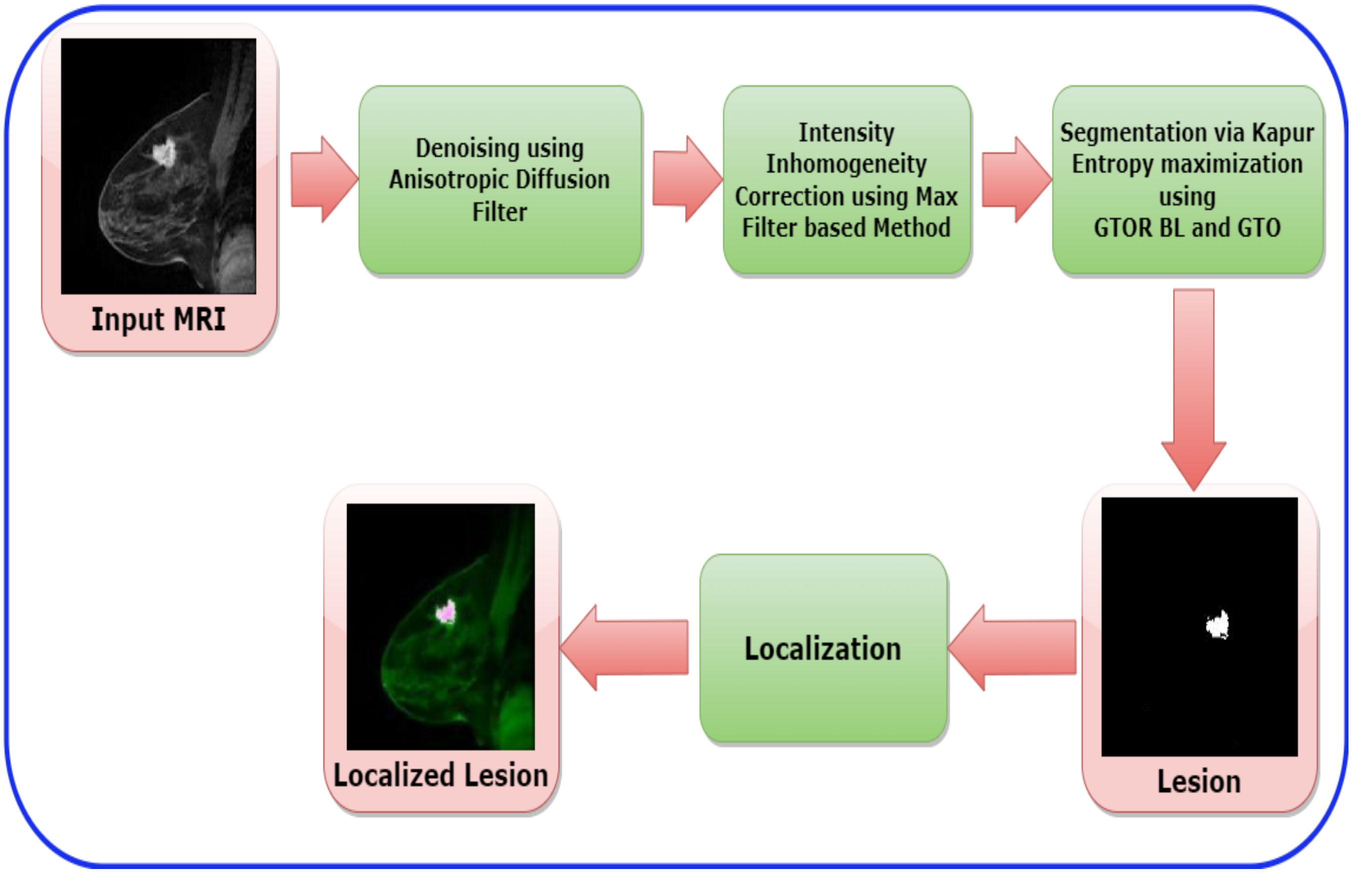

Figure 1. Outline of the proposed methods.

The overall pseudocode and flowchart of the proposed method are given respectively in Algorithm 1 and Fig. 2. In step 1 MR images are read as input. Then in step 2 MR images are denoised using Anisotropic Diffusion Filter (ADF). We have performed denoising during the trial phase ADF, Median filter, and Contrast Limited Adaptive Histogram Equalization (CLAHE). ADF became best based on Peak Signal-Noise Ratio (PSNR) value. It is discussed on subsection 3.3. In step 3 Intensity Inhomogeneities (IIHs) correction using max filter based method. In step 4 MR images are segment using GTO and GTORBL via Kapur entropy maximization. After That in step 5 segment the lesions in the image using optimal threshold. In step 6 lesions are localized in original MR image.

Algorithm 1 Pseudocode of the proposed segmentation method

1: Read MR image
2: Denoised input image using ADF
3: Correction of denoised image using Max filter based method
4: Segmentation using GTORBL (Algorithm 4) via Kapur entropy maximization
5: Segment the lesions in image using optimal threshold
6: Lesions are localized in the original MR image

2.3 Pre-processing

The visual quality of MRIs is crucial for accurate diagnosis and treatment of the patients, and it is affected by the presence of noise generated during the acquisition procedure. Both segmentation process[54] and clinical diagnostic functions are affected by the noise. The Intensity Inhomogeneities (IIHs) in MRI slices also entangle the process of segmentation. IIHs in MRIs are the smooth intensity variations within a previously homogeneous region[7]. Median filter, Contrast Limited Adaptive Histogram Equalization (CLAHE)[65], and Anisotropic Diffusion Filter (ADF) are tested to denoise the DCE-MRIs in the trial phase of this current study. It is noticed that mean peak signal noise ratio (PSNR)[31] values are 20.89, 20.79, and 21.16, over 100 slices. Standard deviation values are 0.4314, 0.5404, and 0.0147. In this result, ADF becomes a better method for denoising than

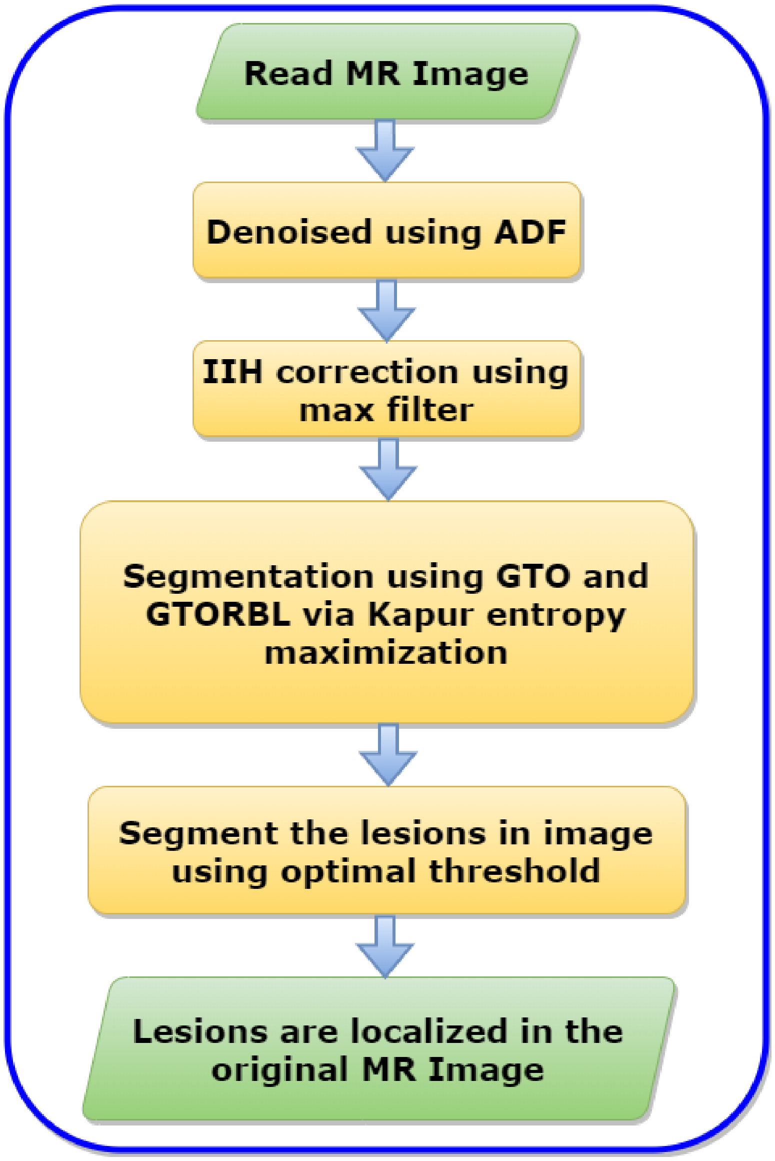

Figure 2. Flowchart of the Proposed Segmentation Algorithm.

median filter and CLAHE. For that reason, ADF[54] is opted to denoise the MRIs in the current study.
A Max filter[7] is applied to correct the IIHs. The inhomogeneous image ($\mathscr{I}_{ih}$) is represented as follows:

$$\mathscr{I}_{ih} = \mathscr{I}_o \times \mathscr{H} + \mathscr{N} \tag{1}$$

where $\mathscr{H}$ indicates an inhomogeneity bias field, $\mathscr{N}$ denotes noise, and a homogeneous image indicates $\mathscr{I}_o$.

2.4 Segmentation

By separating DCE-MRI into non-overlapping parts, segmentation is a technique for isolating lesions from the normal tissues and background. Global thresholding, local thresholding, and traditional approaches based on image histograms are all used in this breast lesions segmentation process. This work uses the metaheuristics GTORBL and GTO to homogeneity parameter thresholds, resulting in pixel-based area expansion strategies.

2.4.1 Maximization of Kapur Entropy

The bilevel thresholding[39] method divides an image into background and foreground. This is not applicable to a complicated image having multiple objects. Therefore, the multilevel thresholding techniques replaces the bilevel thresholding and achieves

the optimal threshold values for image segmentation. Kapur's entropy is a practical statistic for multilevel thresholding segmentation. The image is split into various classes, and the entropy determines whether the category is uniform. The calculating technique for Kapur's entropy is straightforward, and it is simple to implement. It has a great degree of consistency and can be processed quickly. In addition, it provides a high level of accuracy in segmentation. Image segmentation utilising multilayer thresholding and Kapur entropy minimization employs metaheuristics such as the Moth-Flame optimization (MFO) method and the Crow Search Algorithm (CSA).

A rapid recursive segmentation technique based on Kapur's entropy was utilised by Upadhyay and Chhabra[92]. Based on testing results, the proposed technique can boost the speed of calculation in five-level thresholding by 70 times in comparison to the traditional Kapur's method. Using the crow search method, Kiani et al.[42] presented Kapur's entropy optimum multilevel segmentation of image. To attain ideal threshold values, Kapur's entropy is utilised as a fitness function, and the entropy is maximised using the CSA. The Kapur's entropy is maximised using the crow search technique. For each candidate solution, a collection of random threshold values is first encoded. The excellence of the first solution is determined through the fitness function using Kapur's entropy. Kapur's entropy technique for multilevel thresholding is discussed as follows:

Consider the following image, which has a total of G grey levels in span 0 to $G-1$ and a sum of N pixels. $f(i)$ is the frequency of the ith intensity level.

$$N = f(0) + f(1) + f(2) + \cdots f(G-1). \tag{2}$$

Eq. (3) gives the probability of the ith intensity level.

$$P_i = \frac{f_i}{N} \tag{3}$$

Consider the following M thresholds: $th_1, th_2, \cdots th_M$, where $1 \leq M \leq G-1$. Applying these thresholds, image is segmented into $M+1$ segments and notations are as follows: $Class(0) = \{0,1,2,\cdots,th_1-1\}$, $Class(1) = \{th_1, th_1+1,\cdots,th_2-1\}$ and $Class(M+1) = \{th_M-1, th_{M-1}+1,\cdots,th_M\}$. The method based on entropy is defined as given in Eq. (4). F (th_1, th_2, $\cdots$, th_M) = E_0 + E_1 + $\cdots$ + E_M, Where, E_i is the entropy of ith class.

$$\begin{aligned}
E_0 &= -\sum_{i=0}^{i=th_1-1} \frac{P_i}{w_0} \ln \frac{P_i}{w_0}, \quad w_0 = \sum_{i=0}^{i=th_1-1} P_i \\
E_1 &= -\sum_{i=th_1}^{i=th_2-1} \frac{P_i}{w_1} \ln \frac{P_i}{w_1}, \quad w_1 = \sum_{i=th_1}^{i=th_2-1} P_i \\
E_M &= -\sum_{i=th_M}^{i=G-1} \frac{P_i}{w_M} \ln \frac{P_i}{w_M}, \quad w_M = \sum_{i=th_M}^{i=G-1} P_i
\end{aligned} \tag{4}$$

After the denoised and IIH corrected image are obtained, entropy maximization is carried out to find the suitable thresholds for segmentation. By expanding the number of homogeneous regions between them, entropy is maximized. The value of entropy is computed using the frequencies of pixel from histograms (Figures 3(b) & 3(d) depicts the histograms of MR images shown respectively in Figures 3(a) & 3(c)). The entropy function, i.e. objective function is being maximized by GTORBL and GTO. As the gray levels of the image exist in $[0,255]$, the anticipated solutions, i.e. threshold values are integer values in the same range. The individual search agents represent the set of threshold values that are in the aforementioned range. The operators of GTORBL and GTO updates these individual solutions during the search process. The optimal threshold values are obtained after the termination of search algorithms and applied to segment the images. The GTO algorithm is discussed next.

2.4.2 Gorilla Troops Optimization

Five distinct operators are utilised in the GTO algorithm for optimization operations based on gorilla behaviours. Three different operators were used: migration to an unknown location to boost GTO investigation. The second operator improves the balance. The third operator considerably improves the GTO's ability to find alternative optimization areas. All gorillas are defined as candidate solutions in the GTO algorithm, and the best candidate solution at every optimization operation stage is referred to as a silverback gorilla. Each of these three techniques is chosen using a standard procedure.

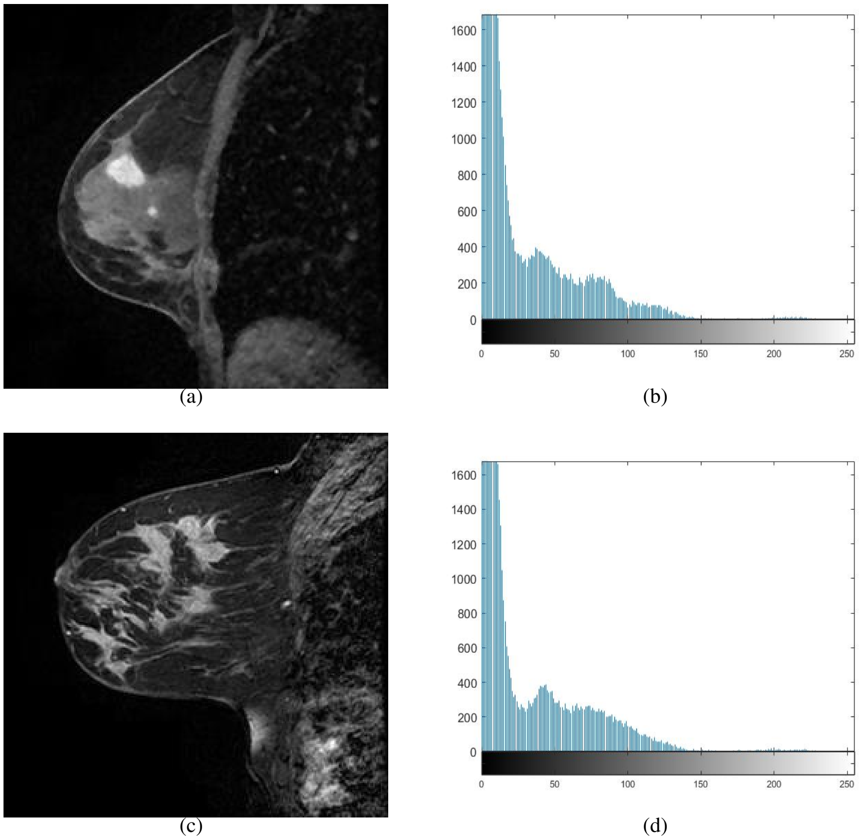

Figure 3. T2 weighted sagittal DCE-MRIs and histograms.

The technique of migration to an unknown site is chosen using a variable named p. When rand p is used, the first mechanism is chosen. If $rand \geq 0.5$, however, the gorilla-to-gorilla movement mechanism is chosen. If $rand < 0.5$, however, the technique of migration to a mentioned place is chosen. The first technique allows the algorithm to keep a close eye on the whole problem area, the second technique increases GTO effectiveness, and the third technique helps the GTO avoid local optimal locations. Eq. (5) is applied to simulate the three techniques applied in the exploration stage.

$$X^g(t+1) = \begin{cases} (X_{\max} - X_{\min}) \times r_1 + X_{\min}, & rand < p \\ (r_2 - C) \times X_r(t) + L \times H & p \geq 0.5 \\ X(i) - L \times (L \times (X(t) - X_r^g(t)) + r_3 \times (X(t) - X_r^g(t))), & rand < 0.5 \end{cases} \tag{5}$$

In Eq. (5), $X^g(t+1)$ is the location vector of gorilla in the next $(t+1)$ phase. $X(t)$ is the present position vector of the gorilla. r_3, r_2, r_1, and $rand \in (0,1)$ is random values modified in every stage. p is a variable in the span $(0, 1)$. The upper and lower boundaries of the parameters are denoted by $X_{\max}$ and $X_{\min}$, respectively. X_r^g is one of the gorillas in the group who is chosen at random from the entire population, as well as X_r. C, L, and H are finally evaluated using Eqs. (6), (7) and (8) respectively.

$$C = F \times (1 - t) \tag{6}$$

$$F = cos(2 \times r_4) + 1, \tag{7}$$

$$L = C \times l \tag{8}$$

F is determined using Eq. (7). cos represents the cosine function, and $r_4 \in (0,1)$ represents random values that are modified in every stage. Eq. (8) is applied to evaluate L, where l denotes random value in the span 1 and 1. To replicate silverback leadership, Eq. (8) is employed. Lack of experience in group leadership, the silverback gorilla may not be able to make the best decisions to find food the group in the real world; however, with enough experience, he achieves good stability in his leadership.

Also, in Eq. (5), H is computed using Eq. (9), while in Eq. (9), Z is computed using Eq. (10), where Z indicates random values in the span of $(-C, C)$.

$$H = Z \times X(t), \tag{9}$$

$$Z = [-C, C], \tag{10}$$

A team formation is carried out at the last of the exploration stage. The value of every GX solution is manipulated at the last of the exploration stage, and if the value is $X^g(t) < X(t)$, the $X^g(t)$ is utilized as the $X(t)$. As a result, the best solution created during this stage is referred to as a silverback.

As indicated with the two processes utilised in the exploitation phase, the C value in Eq. (6) can be used for competition for senior females or follow the silverback. If $C \geq W$ then following the silverback technique is chosen while adult females' competition is taken if $C < W$. The value W must be specified before to the optimization process.

The silverback is a young and healthy gorilla, and the other males in the team are likewise young and closely follow him. They also obey Silverback's commands to go to different sites in quest of food supplies while remaining with him. Members might also have an impact on the overall movement of the group. When the $C \geq W$ is chosen, this technique is chosen. To imitate this behaviour, Eq. (11) is employed.

$$X^g(t+1) = L \times M \times (X(t) - X^s) + X(t), \tag{11}$$

$$M = \left(\left| \frac{1}{N} \sum_{i=1}^{N} X_i^g(t) \right|^m \right)^{\frac{1}{m}} \tag{12}$$

$$m = 2^L, \tag{13}$$

In Eq. (11), X^s is the silverback position vector.
N is the total number of gorillas. If $C < W$, the second technique is chosen in the exploitation stage.

$$X^g(i) = X^s - (X^s \times Q - X(t) \times Q) \times A, \tag{14}$$

$$Q = 2 \times r_5 - 1, \tag{15}$$

$$A = \beta \times E, \tag{16}$$

$$E = \begin{cases} N_1, & rand \geq 0.5 \\ N_2 & rand < 0.5 \end{cases} \tag{17}$$

Q is thought to represent the force of impact. r_5 is a random variable ranging from 0 to 1 in Eq. (15). E is applied to simulate the influence of violence on the areas of results, and is a variable that should be provided a value by the optimization function. If $rand < 0.5$, E's value will be equal to random values from the normal distribution and the problem's dimensions, but if $rand \geq 0.5$, E will be equal to a random value from the normal distribution. An operation of group formation is performed at the end of the exploitation stage, in which the cost of all X^g is evaluated, and if the cost of $X^g(t) < X(t)$, the $X^g(t)$ is employed as the $X(t)$, and the best solution produced among the entire population is viewed as a silverback. The GTO algorithm is given in Algorithm 2.

Algorithm 2 GTO

The population size N and maximum number of iterations T and parameters β and p
Initialize the random population $X_i (i = 1, 2, \cdots, N)$
Calculate the fitness values of Gorilla
while (stopping condition is not met) **do**
    Update the C using Eq. (6)
    Update the L using Eq. (8)
    for each Gorilla (X_i)) **do**
        Update the location Gorilla using Eq. (5)
    end for
    Calculate the fitness values of Gorilla
    if GX is better than X, replace them
    Set $X_{silverback}$ as the location of silverback
    for (each Gorilla (X_i)) **do**
        if ($|C| \geq 1$) **then**
            Update the location Gorilla using Eq. (11)
        else
            Update the location Gorilla using Eq. (14)
        end if
    end for
    Calculate the fitness values of Gorilla
    if New Solutions are better than previous solutions, replace them
    Set $X_{silverback}$ as the location of silverback
end while
Return $X_{BestGorilla}$, bestFitness

2.4.3 Rotational Opposition based learning GTO

Rotated-Based Explanation of OBL

The primary idea underlying OBL is to consider an estimation and its inverse estimation at the same time in order to develop a candidate solution that is nearer to the global optimum. Rahnamayan et al.[67] developed a Euclidean distance for optimal solution demonstration, which demonstrates intuitively why evaluating the reverse of a candidate solution is preferable to another random option. The key concepts of the OBL mechanism are as follows.

Opposite Number : Let z ε [a, b] be a real number. The opposite number $\check{z}$ is calculated by

$$\check{z} = a + b - z \tag{18}$$

Opposite Point : Let $Z = (z_1, z_2, \cdots, z_D)$ be a point in the D-dimensional search area, where $z_j \varepsilon R_j = [a_j, b_j], j\varepsilon[1, 2, \cdots, D]$ and $Z \varepsilon S = \Pi_{j=1}^{D} R_j$. The opposite point $\check{Z} = (\check{z}_1, \check{z}_2, \cdots, \check{z}_D)$

$$\check{z}_j = a_j + b_j - z_j \tag{19}$$

It's worth noting that the OBL method isn't applied in every search generation. The defined probability variable, known as jumping rate (J_r)[66] is a constant quantity in the range $(0, 1)$ and is commonly established by actual experiences in various opposition-based optimization methods. The survey[97] contains more information regarding OBL.

The centre of the established border in one-dimensional space reflects an opposite number to its original number in the OBL process. A 1-dimensional axis can, however, be placed in a 2-dimensional area to describe the opposing number in a different way.

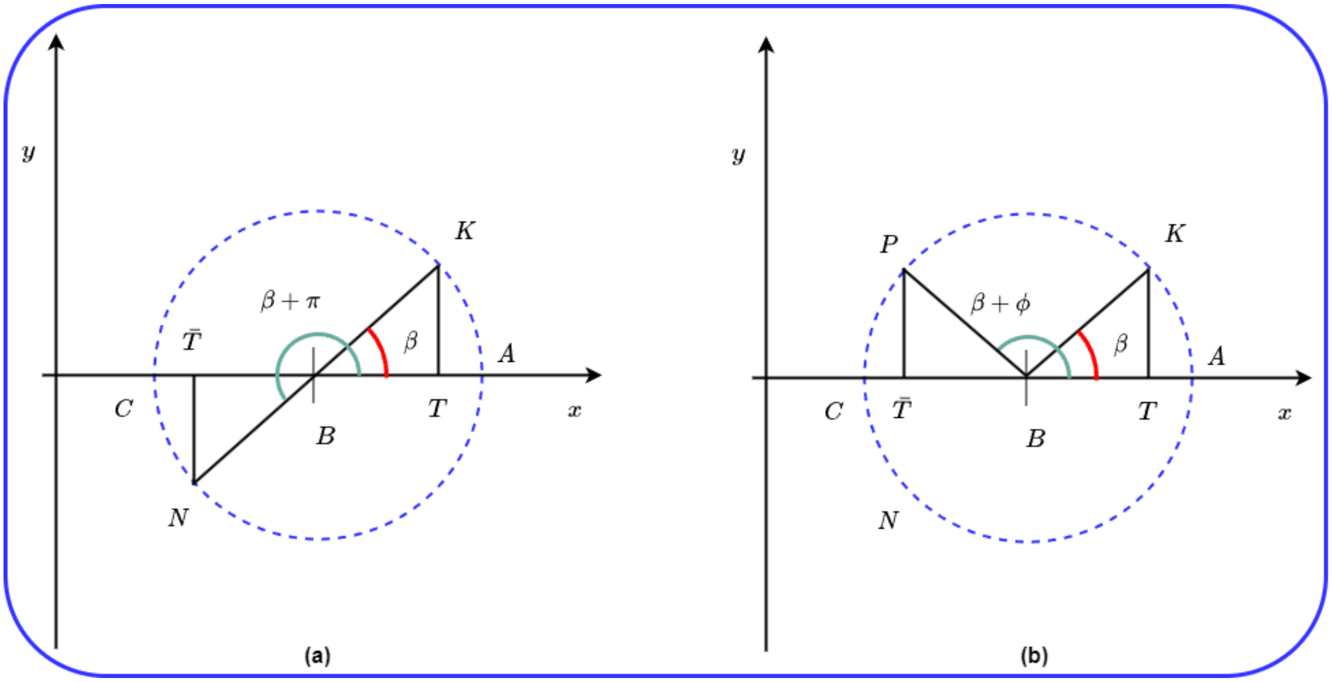

Figure 4. The geometric interpretation of opposite number (a), and rotation number (b) in 2D.

Rotation-Based Learning (RBL) The OBL technique can be extended to an RBL[69] technique by rotating any specified deflection angle, as shown in the analysis above. The geometric interpretation of opposite number and rotation number in 2D space are provided in Figure 4. A new point P is reached by rotating the K point ϕ° counterclockwise around the circle. As a result, $\angle PBA$ has an angle of $\beta + \phi$. Point Z^* is the projection of point N on the x axis, and its coordinate T^* on the x axis is the rotation number of T. Suppose u^* is the amount of the directed line segment $\vec{BT}$, which is produced by:

$$u^* = r \times (\cos(\beta + \phi)) = u \times \cos\phi - v \times \sin\phi \tag{20}$$

T^* can therefore be determined as follows:

$$T^* = (a+b)/2 + u^* \tag{21}$$

It is obvious that the rotation number can find any point in the rotation area by rotating at various angles. Furthermore, the rotation number notion can be simply extended to higher dimensions. Let $T = (T_1, T_2, ..., T_D)$ be a D-variable vector. $a = [a_1, a_2, ..., a_D]$ and $b = [b_1, b_2, ..., b_D]$ are the lower boundary and upper boundary of T, respectively, and $T_i \varepsilon [a_i, b_i]$, $i = 1, 2, ..., D$. Therefore, the center point of rotation area $[a, b]^D$ is marked by $B = (B_1, B_2, ..., B_D)$, and $B_i = (a_i + b_i)/2$. The radius vector is $R = (r_1, r_2, ..., r_D)$, and $r_i = (b_i a_i)/2$. The vector $U = (u_1, u_2, ..., u_D)$ indicates all the quantities of the directed line segment $\vec{B_i T_i}$, and u_i is defined by

$$u_i = T_i - (a_i + b_i)/2 \tag{22}$$

All the sizes of point T_i to its corresponding correlation point K_i on the circle are indicated by the vector $V = (v_1, v_2, ..., v_D)$, and v_i is defined by

$$v_i = \sqrt{(T_i - a_i)(b_i - T_i)} \tag{23}$$

Allow the point T to rotate ϕ° rotating clockwise by the centre point B for each dimension, and the rotation point $T^* = (T_1^*, T_2^*, ..., T_D^*)$ may be obtained, where the T^* i is calculated by

$$T_i^* = (a_i + b_i)/2 + (u_i \times \cos\phi - v_i \times \sin\phi) \tag{24}$$

The suggested RBL mechanism, based on the preceding analysis, can rotate any degree in span 0° and 360° and explore any point in the search area. The RBL mechanism, for example, is equal to the OBL mechanism when the deflection angle is fixed at 180 degrees. The RBL process can be transformed into the quasi-oppositional learning (QOL) mechanism[67] by carefully arranging the deflection angle so that the rotation points fall between the opposition point and the centre point M. As a result, the RBL mechanism can be thought of as a combination of the OBL and QOL mechanisms. Furthermore, RBL can be viewed as a random search if the deflection angle is adjusted arbitrarily between $(0°, 360°)$. As a result, the RBL mechanism is extremely adaptable when it comes to identifying potential promising solutions. The Gaussian distribution is simply utilized to compute the deflection angle in this study, and it is defined as where ϕ and ϕ_0 denote the deflection angle and its basic number, respectively.

$$\beta = \beta_0 . N(1, \sigma) \tag{25}$$

The standard deviation of the Gaussian function P(.,.) is applied to regulate the fluctuation of the basic number and is denoted by σ. An starting value ϕ_0 should be established first before calculating the parameter. σ and ϕ_0 will be set to 0.25 and 180°, respectively, in the following trials. Then, ϕ will oscillate wildly around the 180°, primarily inside [90°, 270°]. This is also the key to RBL's capacity to overcome the disadvantages of the OBL technique. The RBL technique, like the OBL, can be included into a population-based algorithm. To put RBL into effect, first determine the rotation area by evaluating the minimum and maximum values of every dimension for all singles in the population area. The centre point of the rotation area, as well as the lower and upper bound vectors, should all be acquired. The rotation-based population is then constructed, consisting of rotation-based individuals calculated by (22)–(24). The proposed algorithm is given in Algorithm 4.

Algorithm 3 RBL

Require: D dimensional population X of size N

/* Compute the center point C for the population X */
for $j \leftarrow 1$ to D **do**
    $a_j \leftarrow \min_{\forall i} x_{ij}$
    $b_j \leftarrow \max_{\forall i} x_{ij}$
    $c_j \leftarrow \frac{a_j + b_j}{2}$
end for
for $i \leftarrow 1$ to N **do**
/* Compute the new rotation-based individual $X_i^* = (x_{i1}^*, x_{i2}^*, \ldots, x_{iD}^*)$ */
    Set the rotation degree β
    for $j \leftarrow 1$ to D **do**
        Calculate u_{ij} and v_{ij} using Eqs. (22) and (23)
        Calculate x_{ij}^* using Eq. (24)
    end for
end for

Time & Space Complexity Analysis In the proposed GTORBL algorithm (Algorithm 4), steps 1-3 takes time respectively $O(1)$, $O(N.D)$, and $O(N)$ where D is the dimension of the solutions. Step 6 takes $O(N.D)$ as computation of RBL takes $O(N.D)$ and Step 7 takes $O(N)$. Step 8 takes $O(N.\log_2 N)$ if the Quick Sort algorithm is used for sorting. Steps 10-28 all together take $O(T.N.D)$ as the time complexity of GTO is $O(T.N.D)$. Putting all together, the time complexity of GTORBL algorithm is $O(T.N.D)$. Therefore, the time complexities of both GTO and GTORBL algorithms are the same. The space complexity of GTO algorithm is $O(N.D)$ as it store the D dimensional N population in a $N \times D$ array and as there is no requirement of additional memory having size greater than $N \times D$ during the run time. On the other hand, RBL computation takes $O(N.D)$ space and therefore, space complexity of GTORBL is also $O(N.D)$. Hence, the space complexities of both GTO and GTORBL algorithms are also the same.

Algorithm 4 GTORBL

The population size N and maximum number of iterations T and parameters β and p
Initialize the random population $X_i (i = 1, 2, \cdots, N)$
Calculate the fitness values of Gorilla
while (stopping condition is not met) **do**
  if rand $\leq P_{gj}(t)$ **then**
    Generate opposite solutions using RBL
    Evaluate the fitness of opposite solutions
    Select the best n solutions from original and opposite solutions
  else
    Update the C using Eq. (6)
    Update the L using Eq. (8)
    for each Gorilla (X_i)) **do**
      Update the location Gorilla using Eq. (5)
    end for
    Calculate the fitness values of Gorilla
    if GX is better than X, replace them
    Set $X_{silverback}$ as the location of silverback
    for (each Gorilla (X_i)) **do**
      if ($|C| \geq 1$) **then**
        Update the location Gorilla using Eq. (11)
      else
        Update the location Gorilla using Eq. (14)
      end if
    end for
    Calculate the fitness values of Gorilla
    if new solutions are better than previous solutions, replace them
    Set $X_{silverback}$ as the location of silverback (best location)
  end if
end while
Return $X_{BestGorilla}$, bestFitness

2.5 Post-processing

The lesions are eventually extracted from the segmented MR images utilizing GTORBL and GTO-based algorithms in this step. Cluster centres are sorted in increasing order, and cluster numbers are allocated to pixels.

The pixels in the lesional regions are of hyper-intense and hence, are assigned the highest labels which are selected to construct the lesioned image.

Region filling[83] is used for all algorithms to enhance the segmentation outcomes. Using the pixel location of the detected lesions, the lesional regions are eventually overlaid with the actual MRI slice.

3 Setup for experimentation

3.1 Parameter Settings

The competitive algorithms' parameters are obtained from the studies in which they had been developed. The parameter settings are tabulated in Table 1. For all metaheuristic algorithms, the number of search agents is 30, and the maximum number of iterations for all the methods is set to 100. The configuration of the computational environment is provided in Table 2.

Table 1. The parameter settings of algorithms

Algorithm	Parameter	Value
GTORBL	β	3
	W	0.8
	p	0.03
	P_{gj}	0.3
GTO	β	3
	W	0.8
	p	0.03
PSO	W	0.72984
	C_1	1.49618
	C_2	1.49618
SMA	z	0.03
MVO	$WEP_{\min}$	0.2
	$WEP_{\max}$	1
	$TDR_{\min}$	0.6
	$TDR_{\max}$	1
AOA	α	5
	μ	0.5
TSA	$P_{\min}$	1
	$P_{\max}$	4
IMRF	-	-
HMRF	EM iterations	5
	MAP iterations	5
CMRF	Potential	0.5

Table 2. PC Configuration

Name	Configuration
CPU	Intel® Core™ i5-8230U @ 2.30GHz.
RAM	8 GB
Operating System	Windows 10 Home (64-bit)
Software	MATLAB 2021a

4 Evaluation

For the assessment of algorithms' performance, the following metrics are used: accuracy, sensitivity, specificity, F_1-score, FPR, GM, precision, and DSC patra,GAP. The definition of these metrics can be obtained from the study b19y.

5 Results & Discussion

This study develops the lesion segmentation approaches for breast MRI. The MR slices' noise and IIHs make difficulties for segmentation. For that reason, an ADF is used to detect noise from MR images, and IIHs correction is performed in the preprocessing step. The denoised images of MRIs in Figures 5(a) & 5(b) are provided respectively in Figures 6(a) & 6(b). The IIH corrected images after denoising are provided in Figures 7(a) & 7(b). Because the GTORBL and GTO approaches start with a randomly initialised population, the same tests are repeated 10 times for a single image. The quantitative consequences of the mean value and standard deviation value of performance evaluation indicators are analysed using 10×100 findings. The TSA, PSO, AOA, MVO, SMA, HMRF, IMRF, and CMRF procedures are reviewed and compared to the novel methodologies.

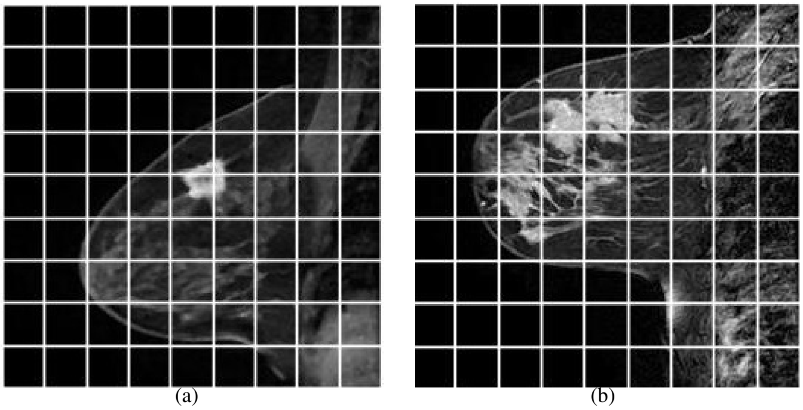

(a) (b)

Figure 5. Original T2 weighted sagittal DCE-MRI.

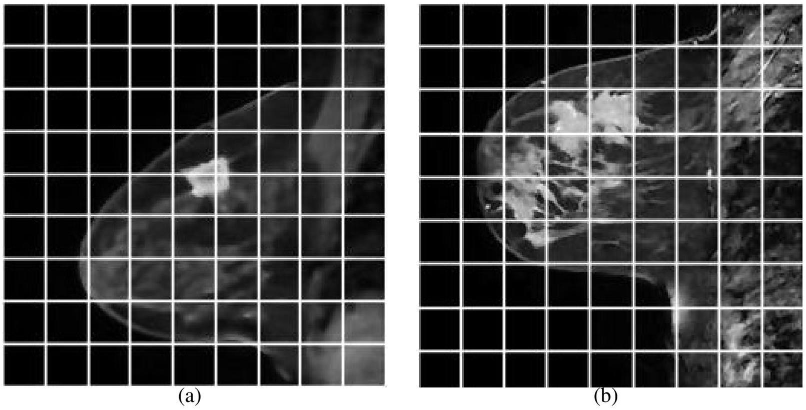

(a) (b)

Figure 6. De-noised images of original T2 weighted sagittal DCE-MRI in Figure 5.

5.1 Quantitative Results

Table 3 presents the quantitative outcomes of the approaches devised, other metaheuristics, and other three existing methods. The boxplot of comprehensive performance of all the approaches over 100 MR images are shown in Figures 8 - 15.

From Table 3, the mean accuracies obtained using the GTORBL and GTO are higher than that achieved by other methods. GTORBL and GTO-based segmentation have respectively 99.31% and 98.13% accuracies, higher than all other compared methods. The mean sensitivity and specificity of the suggested approaches are also higher than those of all techniques that were compared.

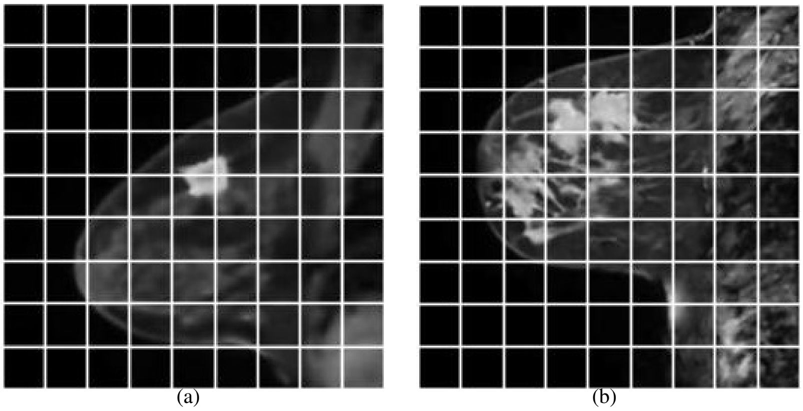

(a) (b)

Figure 7. IIH corrected Images of denoised images in Figure 6.

Precision is another crucial metric for lesion detection. GTORBL and GTO have respectively mean precision of 86.33% and 83.91%, whereas AOA, PSO, TSA, SMA, MVO, HMRF, IMRF, and CMRF have mean precision of 82.11%, 78.96%, 80.92%, 82.74%, 79.79%, 79.49%, 78.83%, and 75.01% which are lower than GTORBL and GTO.

The proposed GTORBL and GTO-based methods, have respectively mean GM of 97.41% and 93.82%, higher than that of all other compared methods.

The FPR, is the proportion of wrongly labelled negative samples to all negative samples. The mean FPR of the GTORBL and GTO is lower than that of the other examined methods. The lower the FPR value, the fewer negative samples in the segmented image there are.

DSC is another crucial metric for lesion detection. The greater the overlap between segmented lesions and Ground Truth (GT), the higher the DSC value. The mean DSC of AOA, PSO, TSA, MVO, SMA, HMRF, IMRF, and CMRF are respectively 83.69%, 83.54%, 83.05%, 83.86%, 85.87%, 82.04%, 82.97%, and 81.51% which are very lower than that of GTORBL and GTO.

It is worth noting that GTORBL achieves higher values for all the metrics than GTO, which indicates that GTORBL quantitatively performs better than GTO.

A boxplot graph depicts the distribution of data in the details. When compared to a density plot, however, boxplots can appear rudimentary. The suggested GTORBL is having a higher median classification accuracy than the other nine current techniques, as shown in Figure 8.

The lowest difference and highest value are quite modest because of the compactness of the accuracy results.

GTORBL is also having a higher median sensitivity than the others shown in Figure 9.

As shown in Figure 10, the recommended GTORBL and GTO have a higher median specificity of categorisation than the other techniques. Figure 11 shows that GTORBL has a greater median precision value than the other techniques. GTORBL has a higher median GM, F_1-score, FPR, and DSC than others as noticed from Figures 12, 13, 14, and 15 respectively.

5.1.1 Robustness Analysis

The robustness of the proposed approaches is also significant. It is computed over numerous independent runs using the standard deviation (std. dev.) of performance metric, and a smaller std. dev. indicates higher robustness. Because the std. dev. values in Table 3 are minimal while comparing with eight current approaches, breast abnormalities can be detected robustly using the suggested segmentation approaches.

5.1.2 Statistical Analysis: ANOVA followed by Tukey HSD

To validate the segmentation performance, a statistical analysis based on DSC was conducted using one-way ANOVA followed by a post-hoc test Tukey HSD.

Table 4 provides the results this test and It is noted that the *p-value* of the ANOVA test is $3.45E-07$ less than the significance level (α) 0.05. Following the ANOVA test, the Tukey HSD statistical test is used to compare the GTORBL and GTO-based approaches to the other methods, and the findings are presented in Table 5 which indicates that GTORBL statistically outperforms the GTO and others with $\alpha = 0.05$.

5.1.3 Statistical Analysis: Wilcoxon Signed Rank Test

A non-parametric test is used to assess the algorithms' performance. To compare the devised GTORBL and GTO methods to the other methods pair-wise, the Wilcoxon Signed Rank Test (WSRT) is used. Tables 6 - 13 describe the test results based on

Table 3. Performance evaluation values (in %) for proposed methods GTORBL and GTO existing methods MVO, PSO, SMA, AOA, TSA, CMRF, IMRF and HMRF

Performance Matrix	GTORBL	GTO	MVO	PSO	SMA	AOA	TSA	HMRF	CMRF	IMRF
Accuracy	**99.31** (0.0238)	98.13 (0.0372)	97.91 (0.0471)	96.86 (0.1049)	96.25 (0.0832)	97.89 (0.0506)	97.63 (0.0549)	97.59 (0.0498)	95.72 (0.0934)	96.83 (0.0583)
Sensitivity	**95.45** (0.0688)	90.96 (0.1171)	90.32 (0.1031)	90.94 (0.1183)	87.88 (0.1165)	89.91 (0.1325)	88.98 (0.1331)	85.59 (0.1487)	86.76 (0.1432)	87.26 (0.1392)
Specificity	**99.37** (0.0235)	98.28 (0.0382)	97.98 (0.0411)	96.92 (0.1035)	96.31 (0.0807)	97.99 (0.0456)	97.68 (0.0544)	97.65 (0.0466)	95.78 (0.0899)	96.91 (0.0567)
Precision	**86.33** (0.1059)	83.91 (0.1065)	79.79 (0.1805)	78.96 (0.1224)	82.74 (0.1635)	82.11 (0.1223)	80.92 (0.1126)	79.49 (0.1796)	75.01 (0.2919)	78.83 (0.1684)
GM	**97.41** (0.0341)	93.82 (0.0711)	92.58 (0.0762)	92.71 (0.0573)	91.92 (0.0912)	91.95 (0.1157)	91.49 (0.1183)	92.63 (0.1091)	91.72 (0.1014)	92.21 (0.0988)
F_1-score	**91.54** (0.0635)	87.04 (0.0628)	83.86 (0.0966)	83.54 (0.0811)	85.87 (0.1357)	83.69 (0.1236)	83.05 (0.1214)	82.04 (0.1107)	81.51 (0.1875)	82.97 (0.2112)
FPR	**0.63** (0.0208)	1.62 (0.0417)	2.32 (0.0237)	3.08 (0.0589)	3.69 (0.0421)	2.01 (0.0181)	2.32 (0.0098)	2.35 (0.0169)	4.22 (0.0559)	3.10 (0.0169)
DSC	**91.54** (0.0635)	87.04 (0.0628)	83.86 (0.0966)	83.54 (0.0811)	85.87 (0.1357)	83.69 (0.1236)	83.05 (0.1214)	82.04 (0.1107)	81.51 (0.1875)	82.97 (0.2112)

Table 4. ANOVA Test based on DSC

Source of Variance	SS	df	MS	F	*p-value*	F-crit.
Between Groups	7937.329	9	881.9255	5.3635	3.45E-07	1.8893
Within Groups	162786.5	990	164.4308			
Total	170723.8	999				

Table 5. Tukey HSD Test based on DSC.

Sl. No.	Pair	Comparison with T	Result (Significant at α=0.05)
1	GTORBL *vs.* GTO	4.5 > 4.1876	yes
2	GTORBL *vs.* PSO	8 > 4.1876	yes
3	GTORBL *vs.* SMA	5.67 > 4.1876	yes
4	GTORBL *vs.* MVO	7.68 > 4.1876	yes
5	GTORBL *vs.* AOA	7.85 > 4.1876	yes
6	GTORBL *vs.* TSA	8.49 > 4.1876	yes
7	GTORBL *vs.* CMRF	10.03 > 4.1876	yes
8	GTORBL *vs.* HMRF	9.5 > 4.1876	yes
9	GTORBL *vs.* IMRF	8.57 > 4.1876	yes

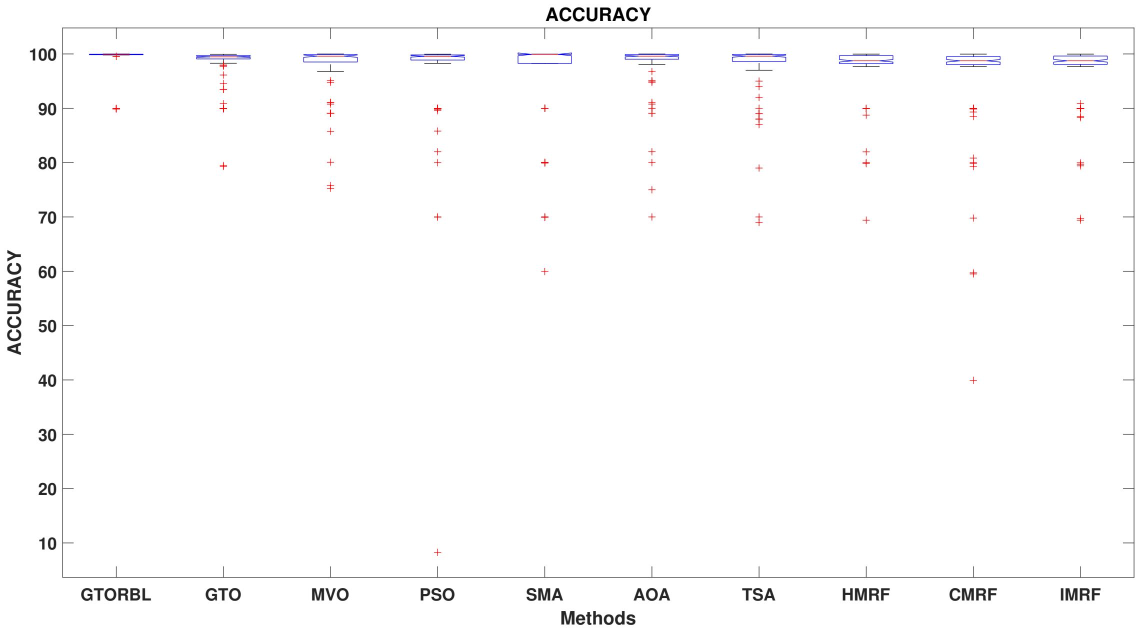

Figure 8. Approaches vs. Accuracy

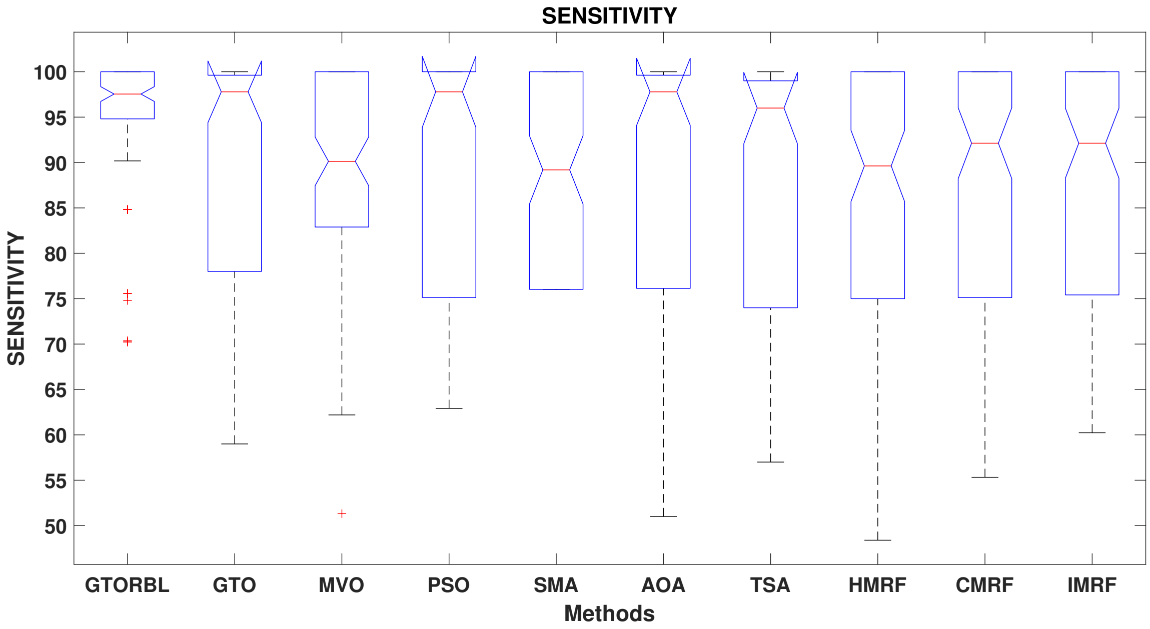

Figure 9. Approaches vs. Sensitivity

accuracy, sensitivity, specificity, precision, GM, F_1-score, FPR, and DSC, respectively.

The significance level (α) of this is 0.05. The α value is adjusted using Bonferroni correction technique[30]. The adjusted α for GTORBL and GTO are respectively $\tilde{\alpha}_1 = \alpha$ / (no. of comparisons) = 0.05 / 9 = 0.005 and $\tilde{\alpha}_2 = 0.05$ / 8 = 0.00625. For accuracy, Table 6 shows that GTORBL performs statistically better than all other methods except MVO. Between GTORBL and MVO, there is no difference in statistical significance in accuracy. It has also been discovered that GTO outperforms all other methods except MVO. Between GTO and MVO, there is no statistically significant difference. GTORBL's sensitivity is

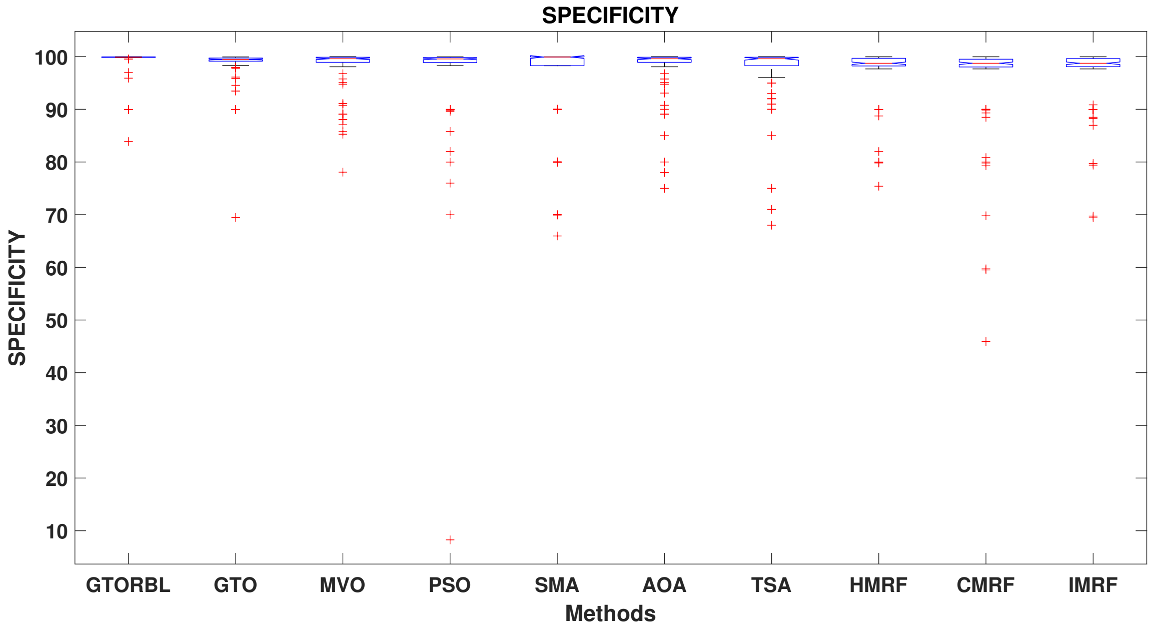

Figure 10. Approaches vs. Specificity

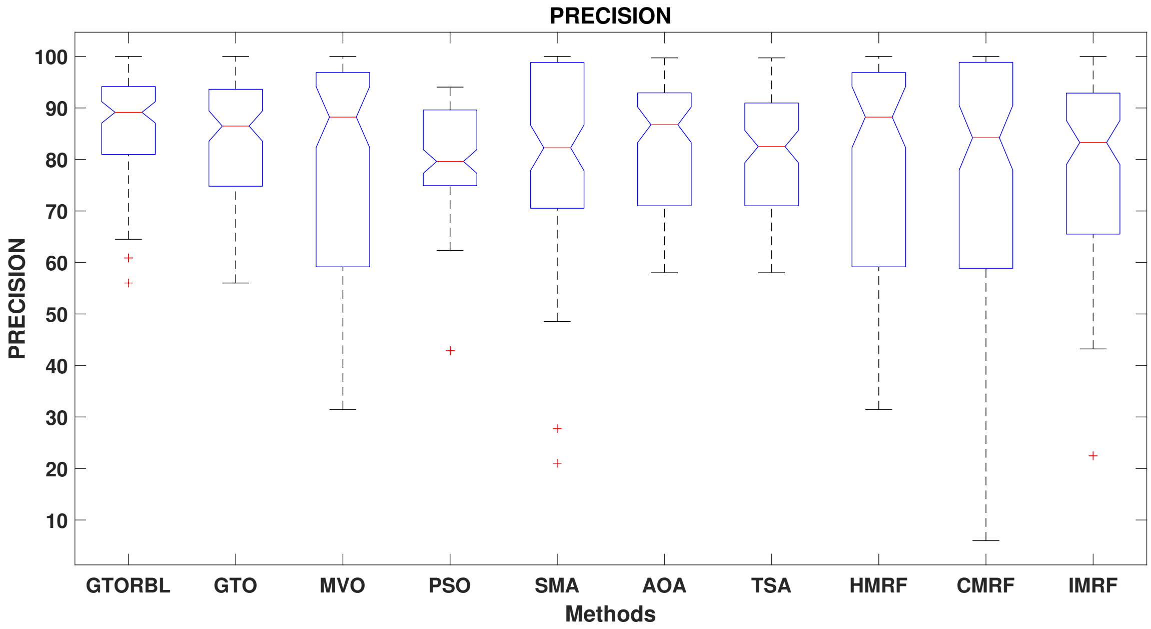

Figure 11. Approaches vs. Precision

statistically better than all compared methods according to Table 7. It has also been discovered that GTO outperforms HMRF and CMRF statistically. GTO and PSO, GTO and SMA, GTO and MVO, GTO and AOA, GTO and TSA, GTO and IMRF have no statistically significant differences. According to Table 8, GTORBL's specificity is also statistically superior to others. It has also been discovered that GTO statistically outperforms TSA, PSO, AOA, SMA, HMRF, CMRF, and IMRF. Between GTO and MVO, there is no difference in statistical significance.

From Table 9, it is observed that

the precision of GTORBL is statistically superior to that of others. It has also been discovered that GTO outperforms TSA, SMA, AOA, HMRF, CMRF, and IMRF statistically. GTO and PSO, as well as GTO and MVO, have no statistically significant

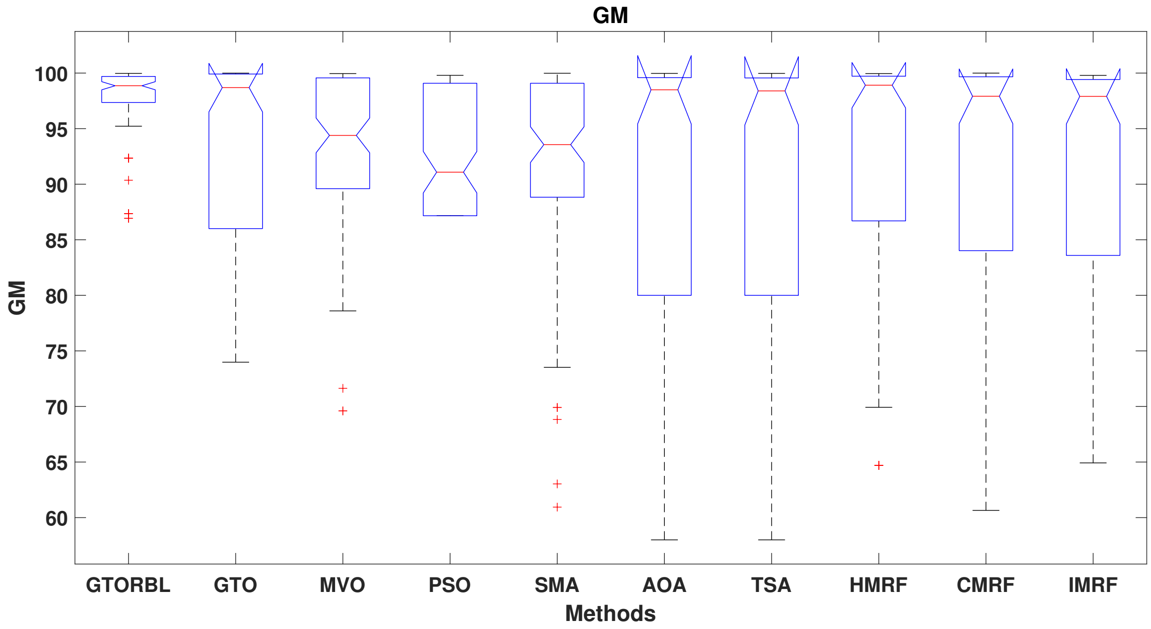

Figure 12. Approaches vs. GM

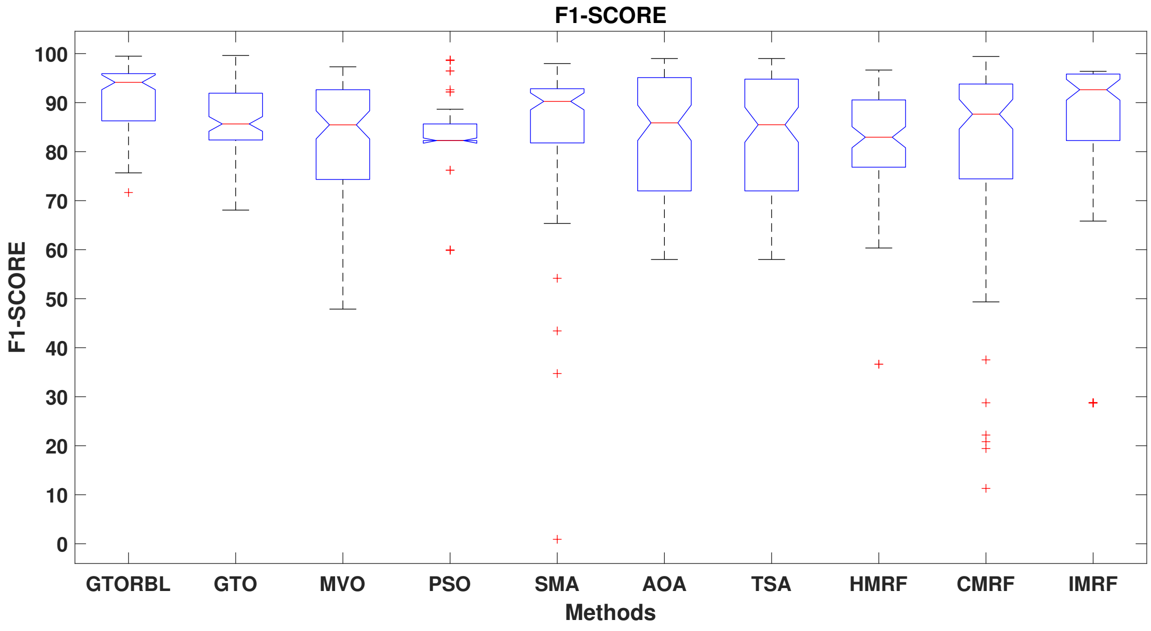

Figure 13. Approaches vs. F_1-score

differences. According to Table 10, the GM of GTORBL is statistically better than others. It has also been discovered that GTO outperforms TSA, PSO, AOA, MVO, CMRF, and HMRF statistically. GTO and SMA, as well as GTO and IMRF, have no statistically significant differences for GM. The F_1-score and DSC of GTORBL are statistically better than others according to Table 11 and 13 respectively. It has also been discovered that GTO outperforms TSA, PSO, AOA, SMA, and CMRF statistically. Between GTO and MVO, GTO and IMRF, and GTO and HMRF, there is no statistically significant difference. The FPR of GTORBL is significantly better than others, according to Table 12. It has also been discovered that GTO also outperforms others except GTORBL statistically.

Table 6. WSRT on accuracy.

Sl. No.	Pair	$p-value$	Pair	$p-value$
1	GTORBL *vs.* GTO	0.000002	GTO *vs.* MVO	0.0011553
2	GTORBL *vs.* MVO	0.0000000	GTO *vs.* PSO	0.00216579
3	GTORBL *vs.* PSO	0.000000	GTO *vs.* SMA	0.726968
4	GTORBL *vs.* SMA	0.068373	GTO *vs.* AOA	0.002369
5	GTORBL *vs.* AOA	0.068373	GTO *vs.* TSA	0.002369
6	GTORBL *vs.* TSA	0.068373	GTO *vs.* CMRF	0.002369
7	GTORBL *vs.* CMRF	0.0000000	GTO *vs.* IMRF	0.000047
8	GTORBL *vs.* IMRF	0.0000000	GTO *vs.* HMRF	0.001305
9	GTORBL *vs.* HMRF	0.0000000		

Table 7. WSRT on sensitivity.

Sl. No.	Pair	$p-value$	Pair	$p-value$
1	GTORBL *vs.* GTO	0.000002	GTO *vs.* MVO	0.0011553
2	GTORBL *vs.* MVO	0.0000000	GTO *vs.* PSO	0.00216579
3	GTORBL *vs.* PSO	0.000000	GTO *vs.* SMA	0.726968
4	GTORBL *vs.* SMA	0.068373	GTO *vs.* AOA	0.002369
5	GTORBL *vs.* AOA	0.068373	GTO *vs.* TSA	0.002369
6	GTORBL *vs.* TSA	0.068373	GTO *vs.* CMRF	0.002369
7	GTORBL *vs.* CMRF	0.0000000	GTO *vs.* IMRF	0.000047
8	GTORBL *vs.* IMRF	0.0000000	GTO *vs.* HMRF	0.001305
9	GTORBL *vs.* HMRF	0.0000000		

Table 8. WSRT on specificity.

Sl. No.	Pair	$p-value$	Pair	$p-value$
1	GTORBL *vs.* GTO	0.000002	GTO *vs.* MVO	0.0011553
2	GTORBL *vs.* MVO	0.0000000	GTO *vs.* PSO	0.00216579
3	GTORBL *vs.* PSO	0.000000	GTO *vs.* SMA	0.726968
4	GTORBL *vs.* SMA	0.068373	GTO *vs.* AOA	0.002369
5	GTORBL *vs.* AOA	0.068373	GTO *vs.* TSA	0.002369
6	GTORBL *vs.* TSA	0.068373	GTO *vs.* CMRF	0.002369
7	GTORBL *vs.* CMRF	0.0000000	GTO *vs.* IMRF	0.000047
8	GTORBL *vs.* IMRF	0.0000000	GTO *vs.* HMRF	0.001305
9	GTORBL *vs.* HMRF	0.0000000		

Table 9. WSRT on precision.

Sl. No.	Pair	$p-value$	Pair	$p-value$
1	GTORBL *vs.* GTO	0.000002	GTO *vs.* MVO	0.0011553
2	GTORBL *vs.* MVO	0.0000000	GTO *vs.* PSO	0.00216579
3	GTORBL *vs.* PSO	0.000000	GTO *vs.* SMA	0.726968
4	GTORBL *vs.* SMA	0.068373	GTO *vs.* AOA	0.002369
5	GTORBL *vs.* AOA	0.068373	GTO *vs.* TSA	0.002369
6	GTORBL *vs.* TSA	0.068373	GTO *vs.* CMRF	0.002369
7	GTORBL *vs.* CMRF	0.0000000	GTO *vs.* IMRF	0.000047
8	GTORBL *vs.* IMRF	0.0000000	GTO *vs.* HMRF	0.001305
9	GTORBL *vs.* HMRF	0.0000000		

Table 10. WSRT on GM.

Sl. No.	Pair	$p-value$	Pair	$p-value$
1	GTORBL *vs.* GTO	0.000002	GTO *vs.* MVO	0.0011553
2	GTORBL *vs.* MVO	0.0000000	GTO *vs.* PSO	0.00216579
3	GTORBL *vs.* PSO	0.000000	GTO *vs.* SMA	0.726968
4	GTORBL *vs.* SMA	0.068373	GTO *vs.* AOA	0.002369
5	GTORBL *vs.* AOA	0.068373	GTO *vs.* TSA	0.002369
6	GTORBL *vs.* TSA	0.068373	GTO *vs.* CMRF	0.002369
7	GTORBL *vs.* CMRF	0.0000000	GTO *vs.* IMRF	0.000047
8	GTORBL *vs.* IMRF	0.0000000	GTO *vs.* HMRF	0.001305
9	GTORBL *vs.* HMRF	0.0000000		

Table 11. WSRT on F_1-score.

Sl. No.	Pair	$p-value$	Pair	$p-value$
1	GTORBL *vs.* GTO	0.000002	GTO *vs.* MVO	0.0011553
2	GTORBL *vs.* MVO	0.0000000	GTO *vs.* PSO	0.00216579
3	GTORBL *vs.* PSO	0.000000	GTO *vs.* SMA	0.726968
4	GTORBL *vs.* SMA	0.068373	GTO *vs.* AOA	0.002369
5	GTORBL *vs.* AOA	0.068373	GTO *vs.* TSA	0.002369
6	GTORBL *vs.* TSA	0.068373	GTO *vs.* CMRF	0.002369
7	GTORBL *vs.* CMRF	0.0000000	GTO *vs.* IMRF	0.000047
8	GTORBL *vs.* IMRF	0.0000000	GTO *vs.* HMRF	0.001305
9	GTORBL *vs.* HMRF	0.0000000		

Table 12. WSRT on FPR.

Sl. No.	Pair	$p-value$	Pair	$p-value$
1	GTORBL *vs.* GTO	0.000002	GTO *vs.* MVO	0.0011553
2	GTORBL *vs.* MVO	0.0000000	GTO *vs.* PSO	0.00216579
3	GTORBL *vs.* PSO	0.000000	GTO *vs.* SMA	0.726968
4	GTORBL *vs.* SMA	0.068373	GTO *vs.* AOA	0.002369
5	GTORBL *vs.* AOA	0.068373	GTO *vs.* TSA	0.002369
6	GTORBL *vs.* TSA	0.068373	GTO *vs.* CMRF	0.002369
7	GTORBL *vs.* CMRF	0.0000000	GTO *vs.* IMRF	0.000047
8	GTORBL *vs.* IMRF	0.0000000	GTO *vs.* HMRF	0.001305
9	GTORBL *vs.* HMRF	0.0000000		

Table 13. WSRT on DSC.

Sl. No.	Pair	$p-value$	Pair	$p-value$
1	GTORBL *vs.* GTO	0.000002	GTO *vs.* MVO	0.0011553
2	GTORBL *vs.* MVO	0.0000000	GTO *vs.* PSO	0.00216579
3	GTORBL *vs.* PSO	0.000000	GTO *vs.* SMA	0.726968
4	GTORBL *vs.* SMA	0.068373	GTO *vs.* AOA	0.002369
5	GTORBL *vs.* AOA	0.068373	GTO *vs.* TSA	0.002369
6	GTORBL *vs.* TSA	0.068373	GTO *vs.* CMRF	0.002369
7	GTORBL *vs.* CMRF	0.0000000	GTO *vs.* IMRF	0.000047
8	GTORBL *vs.* IMRF	0.0000000	GTO *vs.* HMRF	0.001305
9	GTORBL *vs.* HMRF	0.0000000		

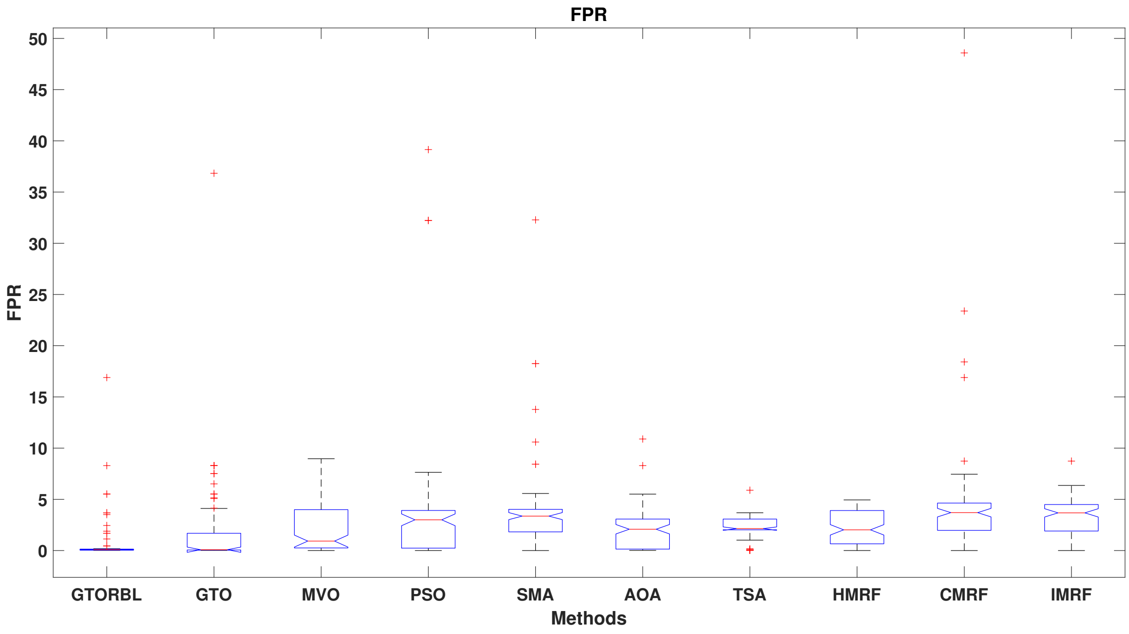

Figure 14. Approaches vs. FPR

Table 14. TOPSIS Rank

Methods	Rank
GTORBL	1
GTO	2
AOA	3
MVO	4
PSO	5
SMA	6
TSA	7
HMRF	8
IMRF	9
CMRF	10

5.1.4 Multi-Criteria Decision Analysis

Multi-Criteria Decision Analysis (MCDA)[11] is carried out using a well-known Technique for Order of Preference by Similarity to Ideal Solution (TOPSIS) method and we have adopted this philosophy from the study[20,79]. All the eight metrics are used as criteria in TOPSIS. The higher values of accuracy, sensitivity, specificity, precision, F_1-score, GM, FPR, and DSC indicates better. FPR contradicts with other metrics, i.e. criteria since lower FPR numbers indicate better. The ranks acquired using the TOPSIS approach are described in table 14. According to Table 14, the GTORBL technique gets the highest ranking. The GTORBL method is then used after the GTO. It's also worth mentioning that CMRF achieves the last rank.

5.2 Visual Results

A total of 100 MRIs of 20 patients are used to validate the proposed segmentation algorithms. Only 2 images of 2 different patients are shown due to space limits, out of 100 test findings. Figures 16 & 17 for respectively patient-1 and patient-2, demonstrate segmented lesions in breast using different methodologies. Figures 18 & 19 show images with a localised lesion, respectively.

When comparing the segmented image in Figure 16(a) obtained from GTORBL with the GT in Figure 16(b), it has been observed that GTORBL almost perfectly segments lesions. In the segmented image obtained from GTO, lesions are almost entirely segmented, as shown in Figure 16(d). GTORBL and GTO are both effective at detecting lesions in images. A few

lesions are not segmented by PSO, as shown in the segmented image in Figure 16(e) generated by PSO. The PSO method is unable to detect lesions while comparing with the GTs.

The segmented image in Figure 16(f) obtained from SMA demonstrates that a few lesions are not segmented. The SMA-based technique does not detect the lesions well when compared to the GTs. The segmented images in Figures 16(g) & 16(h) obtained by MVO and AOA respectively show that they are also unable to segment a small number of lesions. The MVO and AOA are ineffective in detection of lesions.

TSA fails to segment most of the lesions, as observed in the segmented image in Figure 16(i). In the segmented image in Figure 16(j) obtained from HMRF, it can also be shown that HMRF recognises some healthy or normal tissues as lesions and it can't do well in lesion detecting. IMRF fails to segment most of the lesions, as observed in the segmented image in Figure 16(k). In Figure 16(l), the CMRF segmented image, it is observed that CMRF fails to separate the large number of lesions.

For patient-2, when comparing the GT image in Figure 17(b) with segmented image in Figure 17(c) obtained from GTORBL, it is observed that GTORBL segmented lesions practically correctly. It can also be shown in Figure 17(d) that GTO has virtually fully segregated the lesion locations. By comparing the proposed methods to the GT, it is discovered that the developed methods in this current study perform better in the detection of lesions in the MR image. Other metaheuristics-based methods recognise certain healthy tissues as lesions and they do not perform well in lesion detection.

Figures 17(j) & 17(k) show how HMRF and IMRF segment different healthy tissues. These techniques do not perform well in lesion detection when compared to the GTs. Figure 17(l) indicates that CMRF can not segment the large amount of lesions, but rather the lesions at the legion's edge. When compared to the GTs, this technique fails to detect the lesions in the MRI.

5.3 Effects of Pre-processing and Post-processing

We conducted trials without pre-processing, without post-processing, and without both pre and post-processing to investigate the effects of pre-processing and post-processing in the suggested technique. Table 15 gives the mean results and TOPSIS ranks. The TOPSIS approach, like Section 5.1.4, has several criteria for accuracy, sensitivity, specificity, precision, GM, F_1-score, FPR, and DSC.

The GTORBL technique, according to Table 15, has the highest ranking. It is important to note that a method with only post-processing is not more effective than a method having both pre and post-processing.

These findings show that the proposed method's pre-processing and post-processing both help to improve performance of the segmentation.

Table 15. Mean results (in %) and rank of proposal without pre-processing, without post-processing, without pre and post-processing, and proposal

Method \ Metrics	Acc.	Sens.	Spec.	Prec.	GM	F_1-score	FPR	DSC	Rank
Without Pre-processing	97.81	94.23	97.86	85.86	96.34	90.08	2.14	90.08	3
Without Post-processing	98.71	95.13	98.76	86.45	97.51	93.07	1.24	93.07	2
Without Pre and Post-processing	97.21	93.03	97.26	84.15	95.34	89.13	2.74	89.13	4
Proposed Method (GTORBL)	99.10	95.83	99.21	87.05	98.24	94.89	0.79	94.89	1

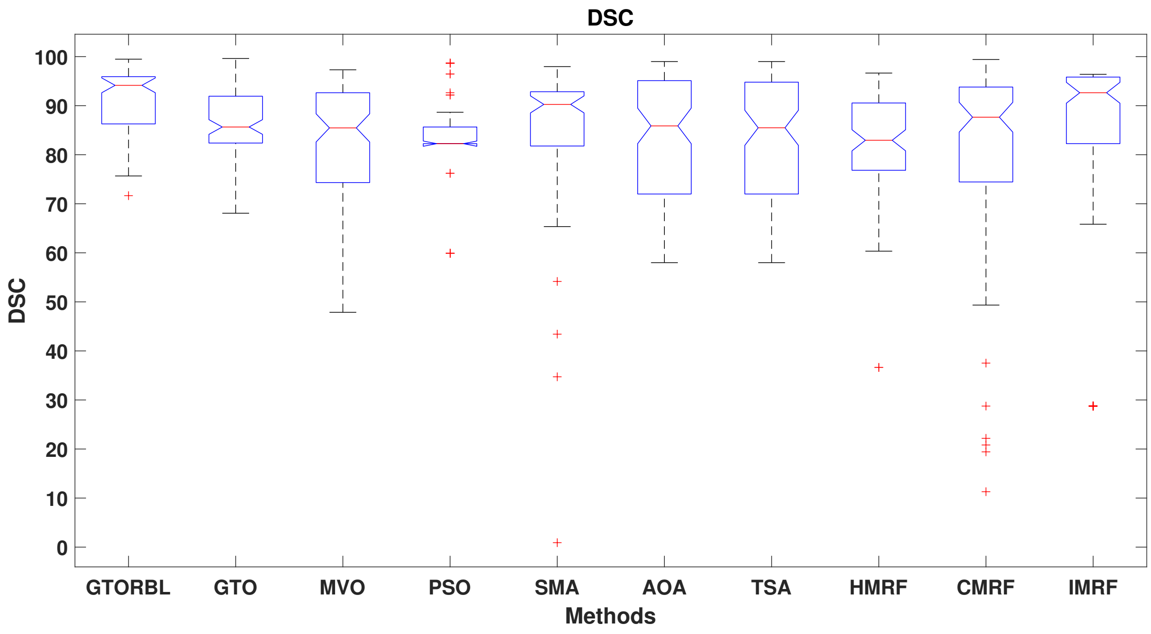

Figure 15. Approaches vs. DSC

Table 16. Mean computational cost.

Methods	GTORBL	GTO	AOA	PSO	TSA	MVO	SMA	HMRF	IMRF	CMRF
CPU time (Seconds)	**3.3465**	3.7514	3.8827	3.9854	4.1025	4.0124	3.8546	4.5503	4.7499	4.8603

5.4 Convergence Analysis

The convergence graphs are shown in Figures 20 & 21 for the image in Figures 16(a) and 17(a), respectively. In graphs, the best entropy values, i.e. the best objective function values, are plotted against the Function Evaluations (FEs). The graph shows how far the search for the best solution has advanced. Graphs show that GTORBL converges better than other metaheuristics. The graph in Figure 20 demonstrates that GTORBL exhibits convergence extremely close to the optimal output after around 100 FEs. GTORBL consistently outperforms GTO and PSO. According to the convergence graph in Figure 21, GTORBL converges very near to the best result after around 500 FEs, but GTO converges extremely close to the best result after about 1200 FEs. GTORBL converges quicker than GTO after 400 FEs. GTORBL regularly has higher values than PSO. PSO also converges prematurely in the local optima. The graphs show the GTORBL's superior searching ability in the maximization of entropy.

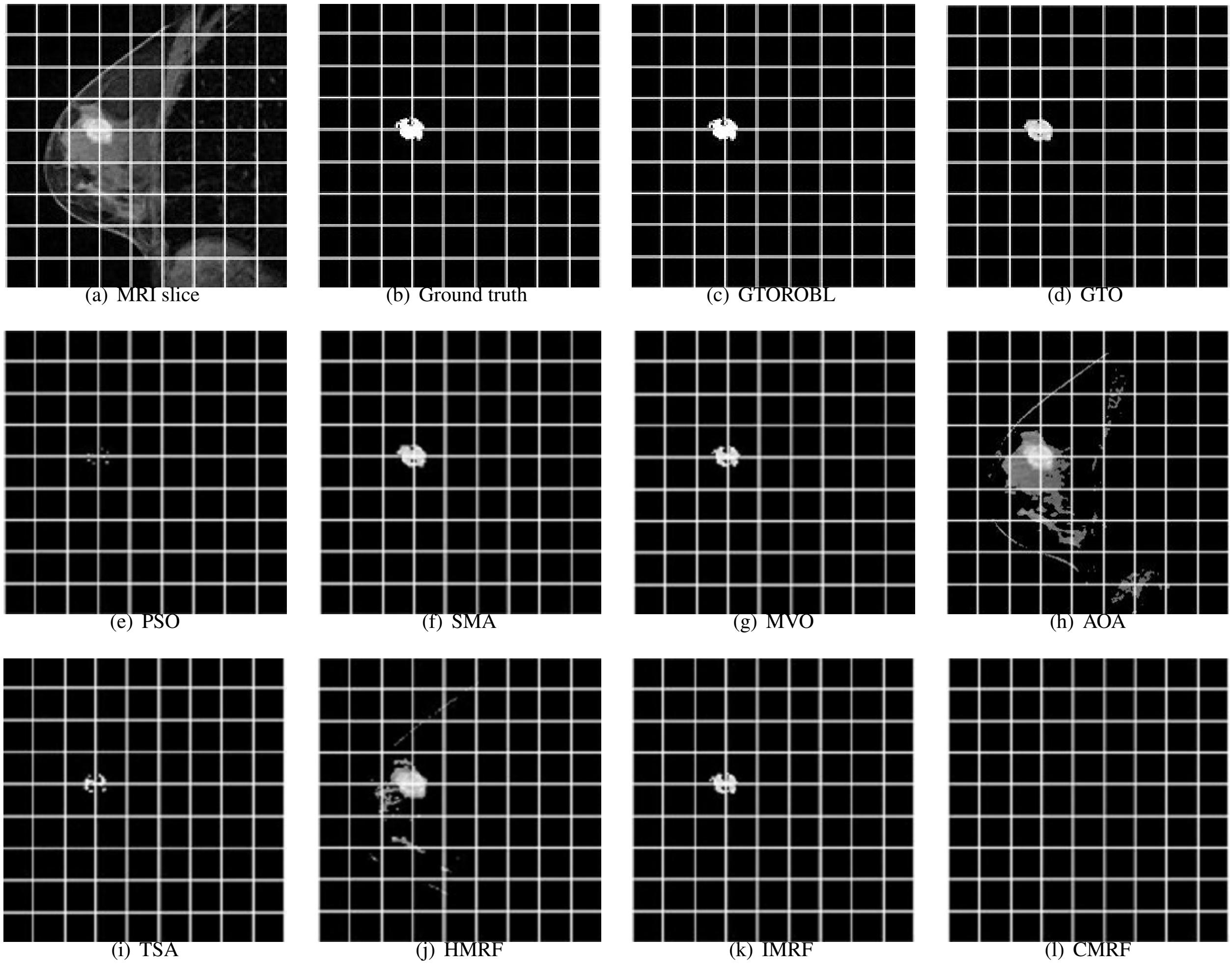

Figure 16. For Patient-1 (a) MRI slice, (b) ground truth, and (c-l) GTORBL, GTO, PSO, SMA, MVO, AOA, TSA, HMRF, IMRF, and CMRF respectively.

5.5 Computational Complexity Analysis

The average computational costs are 0.43 sec and 1.24 sec for respectively denoising and IIH correction.

GTORBL requires a mean computational time cost of 3.3465 seconds, according to Table 16. The mean computational time for GTO is 3.7514 seconds. The mean computational time for PSO is 3.9854 seconds. SMA takes 3.8546 seconds to

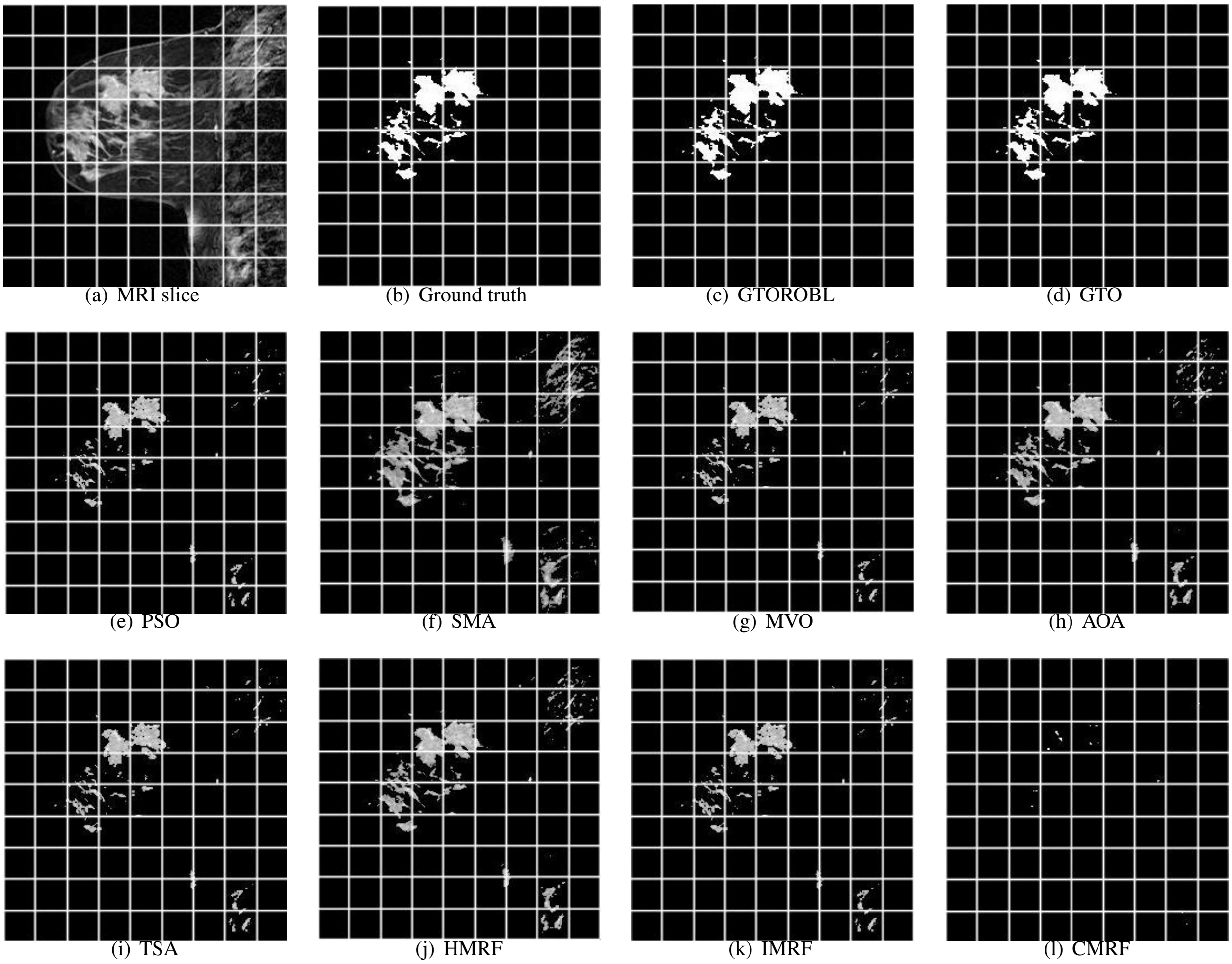

Figure 17. For Patient-2 (a) MRI slice, (b) ground truth, and (c-l) GTORBL, GTO, PSO, SMA, MVO, AOA, TSA, HMRF, IMRF, and CMRF respectively.

complete. The mean computational time for MVO is 4.0124 seconds. The mean computational time for AOA is 3.8827 seconds. TSA uses a mean of 4.1025 seconds of CPU time. The mean computational time for HMRF is 4.5503 seconds. The mean computational time for IMRF is 4.7499 seconds, whereas the mean computational time for CMRF is 4.8603 seconds. GTORBL is faster than all others, whereas GTO is slower than others.

In accordance with the aforementioned evaluation of both quantitative and qualitative (i.e., visual) results, both GTORBL and GTO perform better in the lesion segmentation of breast MRI than all the compared algorithms. GTORBL surpasses GTO in the segmentation of breast lesions. GTORBL outperforms all other algorithms in terms of segmentation consistency, but GTO also outperforms TSA, MVO, AOA, SMA, PSO, CMRF, IMRF, and HMRF. According to the results of the studies, both GTORBL and GTO are efficient and successful in breast lesion segmentation in DCE-MRI.

5.6 Discussion

In this paper, we present a breast tumor segmentation methodology. The proposed methodology makes use of Kapur's entropy maximization characteristics and sagittal T2-weighted fat-suppressed DCE-MRI data. The approach is applied to the DCE-MRI dataset, and as shown in Table 3, it outperforms state-of-the-art techniques in terms of performance. We used ADF for denoising and a max filter-based approach to generate IIHs during the preprocessing stage. In GTORBL, the generation jumping phase produces better exploration in the search space than GTO. Because the search space is not diverse enough, basic GTO suffers from early convergence in local optima. So, we must enhance the GTO to produce solutions of higher caliber. As a result, in this paper, we have developed a modified GTO called GTORBL by incorporating RBL strategy. Entropy maximisation is used to choose the appropriate threshold values for segmentation after obtaining the denoised image. Entropy is intended to be maximised by increasing the number of homogenous sites between them and the entropy value is computed using the

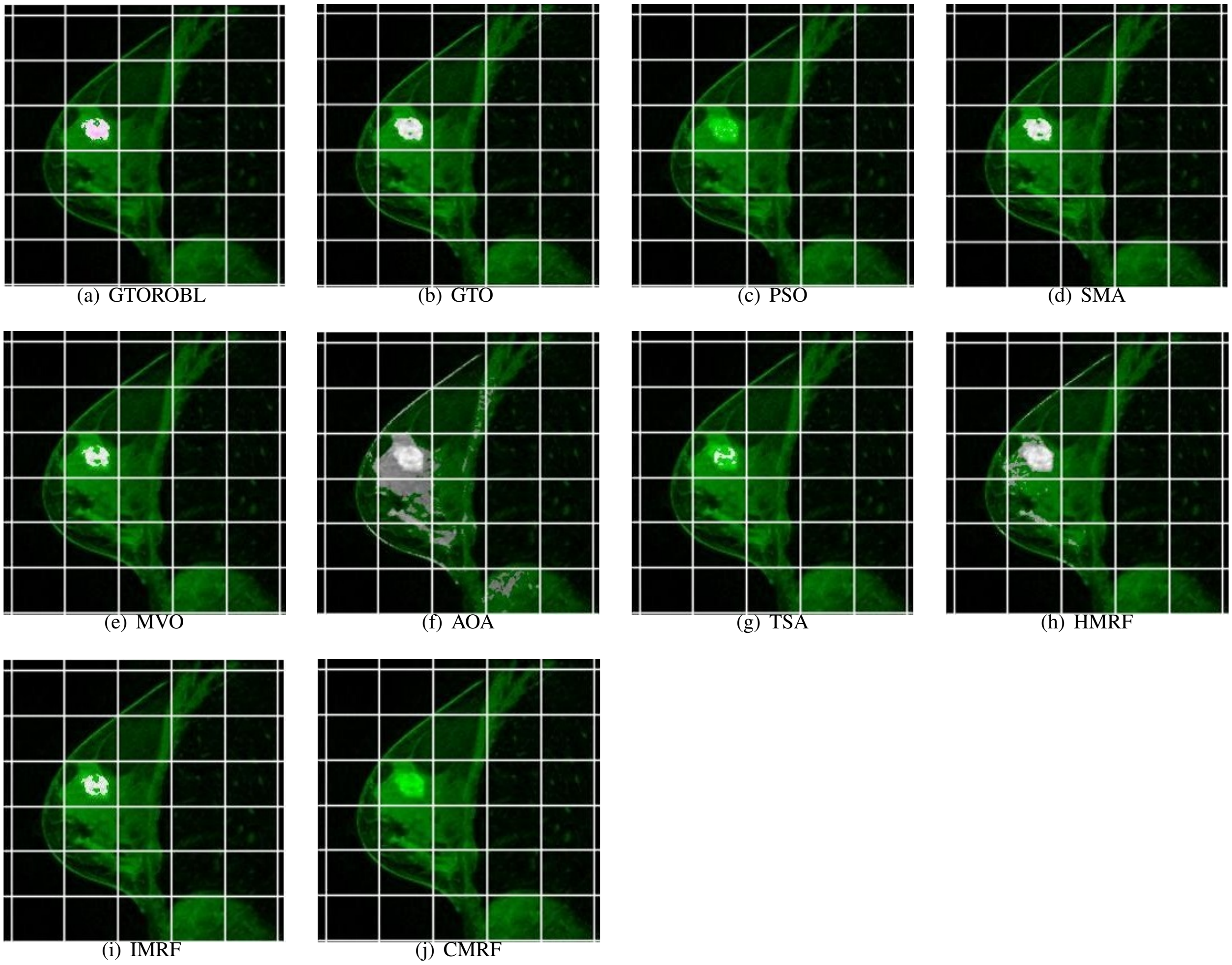

Figure 18. Localized lesions (bright colored spot) in MR images for patient-1 (a) GTORBL, (b) GTO, (c) PSO, (d)SMA, (e) MVO, (f) AOA, (g) TSA, (h) HMRF (i) IMRF, and (j) CMRF.

histograms' pixel frequencies. The GTORBL algorithm maximises the objective function, which is the entropy function. The MR images are segmented using the obtained optimal threshold values and in the postprocessing step, the lesions are extracted from the segmented MR images. For all of the methods, we have employed region-filling to enhance the segmentation outcomes. The one-way ANOVA test and the post-hoc Tukey Honestly Significant Difference (HSD) test are the statistical analysis techniques utilised to analyse the data. A boxplot is a type of graph that displays the distribution of values in the specifics. Contrasted with a density plot, boxplots, on the other hand, may seem rudimentary. Figures 8-15 demonstrate that the proposed GTORBL achieves a higher median classification result than the other nine current techniques. In addition, we employ multi-criteria decision-making to assess overall performance in accordance with the aforementioned criteria. The proposed methodology outperforms the nine examined methodologies in the experiments. The convergence graph reveals the superior entropy maximisation search performance of the GTORBL method. The suggested lesion segmentation method outperforms the compared methods, according to quantitative results using statistical analysis and multi-criteria decision analysis as well as qualitative results.

Although GTORBL surpasses other competing algorithms in this study, the performance of GTORBL can be enhanced with additional changes, such as better swarm initialization using chaos theory[101,102]. In this study, the generation jumping probability P_{gj} is set by *trial-and-error* method and this limitation can be overcome by using deterministic or adaptive rules. The random search technique[103] can also be used to solve this problem.

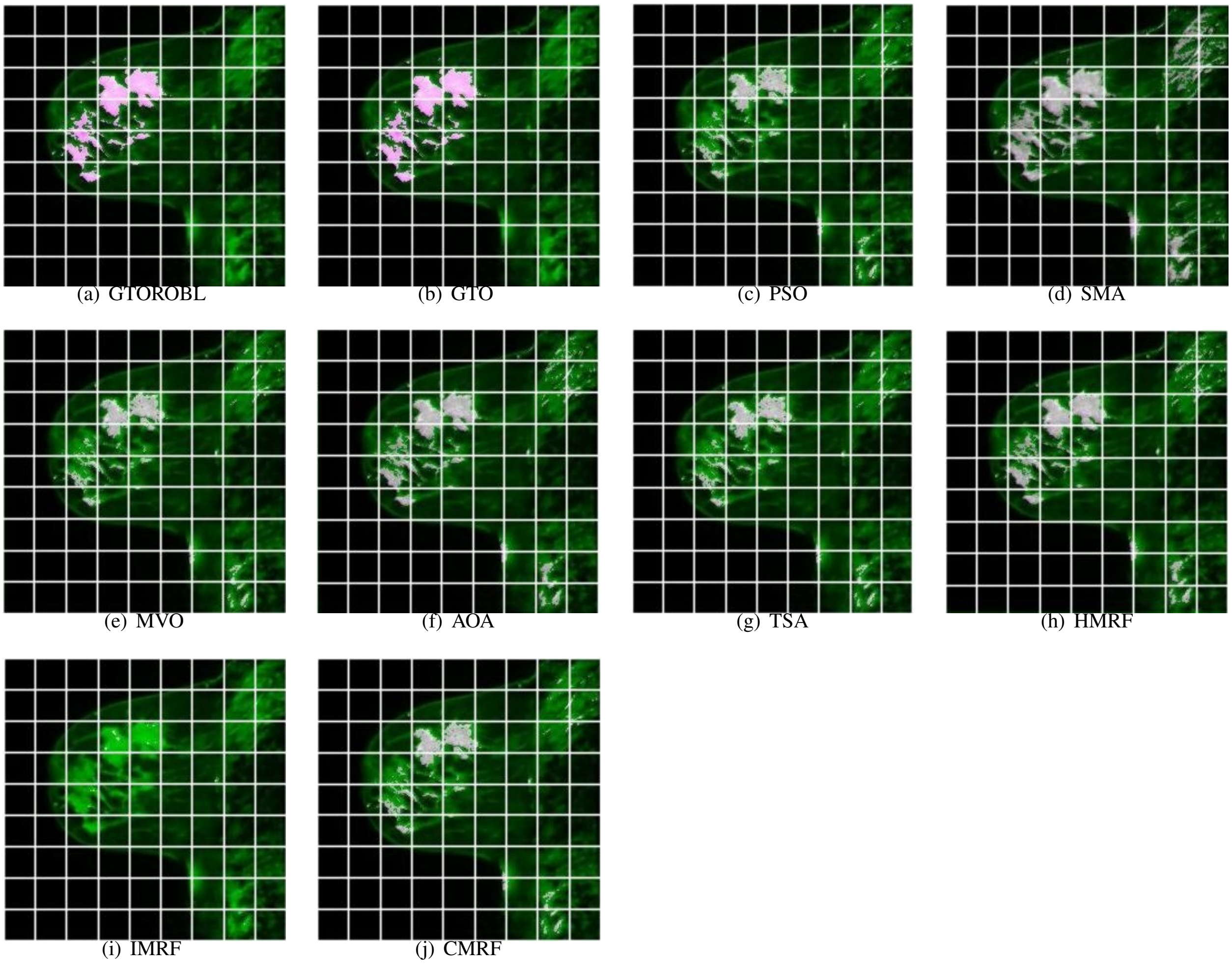

(a) GTOROBL (b) GTO (c) PSO (d) SMA
(e) MVO (f) AOA (g) TSA (h) HMRF
(i) IMRF (j) CMRF

Figure 19. Localized lesions (bright colored spot) in MR images for patient-2 (a) GTORBL, (b) GTO, (c) PSO, (d) SMA, (e) MVO, (f) AOA, (g) TSA, (h) HMRF, (i) IMRF, and (j) CMRF.

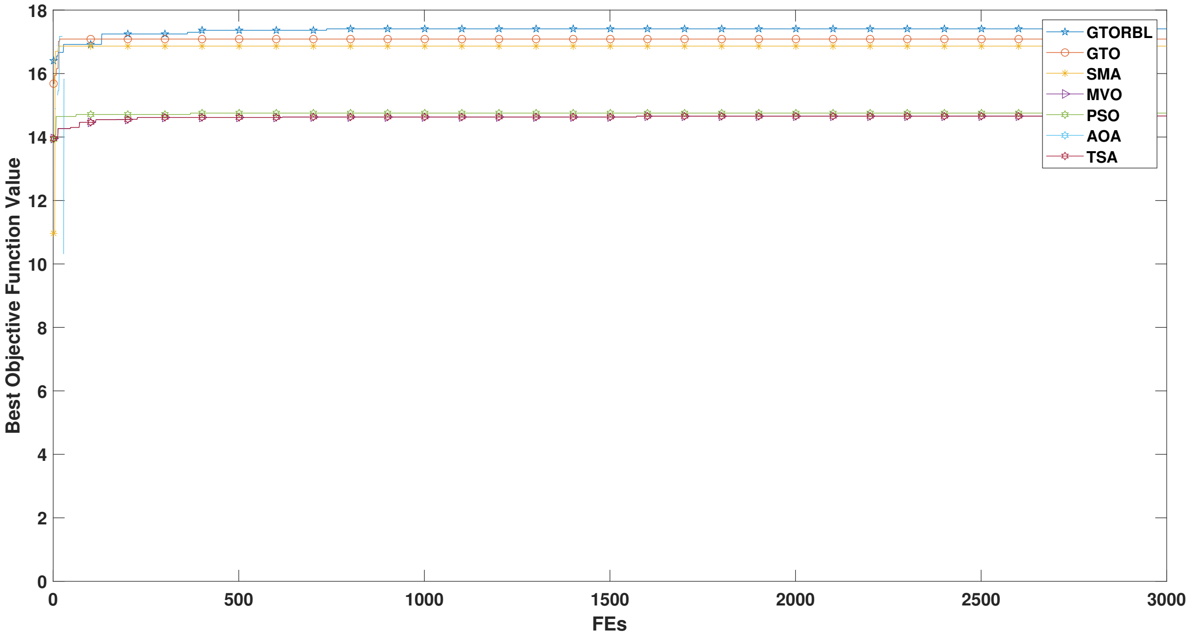

Figure 20. Convergence graph for MRI slice in Figure 16(a).

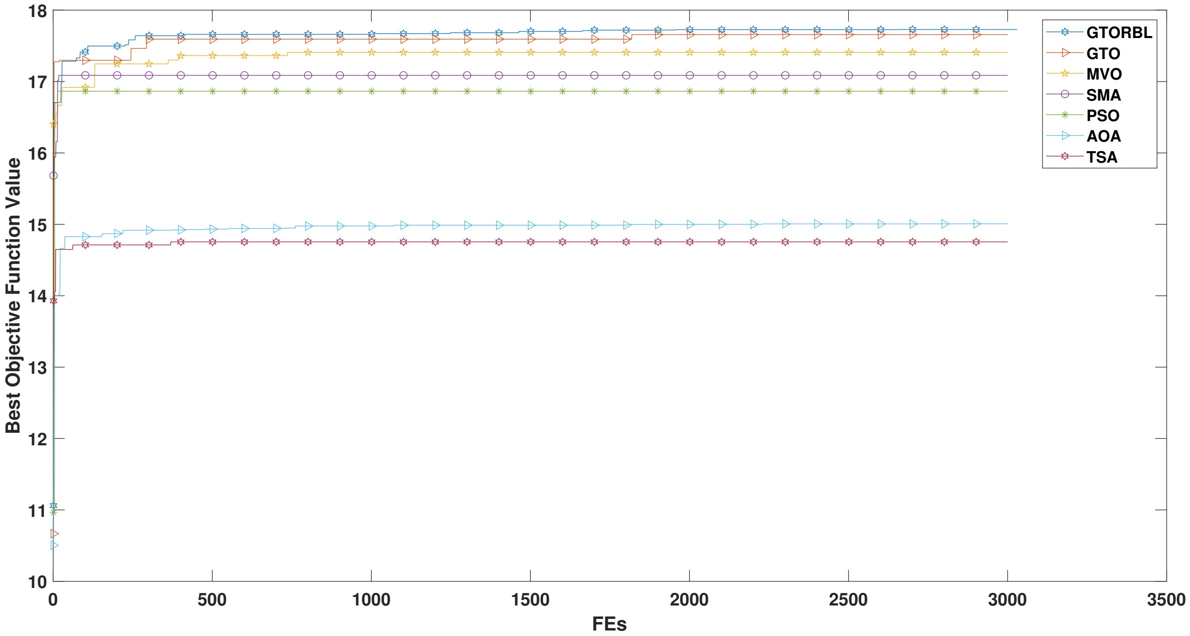

Figure 21. Convergence graph for MRI slice in Figure 17(a).

6 Conclusion with Prospective Future Works

The goals of this present study are the examination and development of a suitable approach for the lesion segmentation in fat-suppressed DCE of breast to assist radiologists and doctors not only in breast cancer diagnosis but also in treatment planning, and surgery. Two segmentation approaches are proposed in this article. GTO is employed in lesion segmentation in breast MRI in the first GTO-based approach. GTORBL is an upgraded variant of GTO that is used in place of GTO in the same framework as the first approach. These two approaches' performance is compared to that of metaheuristics such as TSA, SMA, AOA, PSO, MVO, and the existing breast MRI segmentation methods such as HMRF, CMRF, and IMRF. The results show that the proposed methods surpass these aforementioned competitive methods in terms of specificity, accuracy, precision, sensitivity, F_1-score, GM, FPR, and DSC. Both GTORBL and GTO outperform other compared approaches in the segmentation of breast lesion in MRI, according to the experimental data and analyses utilising statistical approaches and MCDM. In DCE-MRI, GTORBL outperforms GTO in the segmentation of breast lesions. The methods given are effective and successful in detecting lesions in DCE-MRI of the breast.

This current study is limited to T2-W sagittal fat-suppressed DCE-MRI of breast. Therefore, the future investigation leads to apply the proposed method to other Breast MRI sequences such as T1-W, Ultrafast, and diffusion-weighted imaging (DWI). One limitation of proposed GTORBL is setting the value of generation jumping probability which is done empirically in this study and this can be overcome by means of some deterministic or adaptive rules in the future. We also intend to modify GTO in the future by including different OBL techniques. For multi-level thresholding, we employed Kapur entropy in this study. In the proposed methodologies, we want to study different entropies such as Rényi entropy, and Tsallis entropy. The developed approaches will also be used to segment the MRI of brain, kidney, liver, prostate, etc.[104–109].

7 Acknowledgements

We thank the US NSF award 1761839 and a catalyst award from the US National Academy of Medicine.

Availability of Data and Materials

The datasets used and/or analysed during the current study are available in The Cancer Imaging Archive repository, https://wiki.cancerimagingarchive.net/pages/viewpage.action?pageId=3539225

References

1. Abdollahzadeh, B., Gharehchopogh, FS. & Mirjalili, S. Artificial gorilla troops optimizer: A new nature-inspired metaheuristic algorithm for global optimization problems. Int J Intell. Syst. 1-72. DOI: 10.1002/int.22535 (2021).
2. Abualigah, L., Diabat, A., Mirjalili, S., Elaziz, M. A. & Gandomi, A. H. The Arithmetic Optimization Algorithm. Comput. Methods Appl. Mech. Engrg. 376 (113609) (2021).
3. Agner, SC., Soman, S., Libfeld, E., Mcdonald, M., Thomas, K., Englander, S., Rosen, MA., Chin, D., Nosher, J. & Madabhushi, A. Textural Kinetics: A Novel Dynamic Contrast-Enhanced (DCE)-MRI Feature for Breast Lesion Classification. Journal of Digital Imaging 24, 446-463 (2011).
4. AlQoud, A. & Jaffar, MA. Hybrid gabor based local binary patterns texture features for classification of breast mammograms. International Journal of Computer Science and Network Security 16(4), 16-21 (2016).
5. Anscombe, F. The Validity of comparative experiments. Journal of the Royal Statistical Society 111(3), 181–211 (1948).
6. Azmi, R. & Norozi, N. A new markov random field segmentation method for breast lesion segmentation in mr images. Journal of Medical Signals Sensors 1, 156–164 (2011).
7. Balafar, MA., Ramli, AR. & Mashohor, S. A new method for MR grayscale inhomogeneity correction. Artif. Intell. Rev 34, 195-204 (2010).
8. Benjelloun, M., Adoui, ME., Larhmam, MA. & Mahmoudi, SA. Automated Breast Tumor Segmentation in DCE-MRI Using Deep Learning. 2018 4th International Conference on Cloud Computing Technologies and Applications (Cloudtech). IEEE. doi=doi.10.1109/CloudTech.2018.8713352 (2018).
9. Bora, VB., Kothari, AG. & Keskar, AG. Robustautomatic pectoral muscle segmentation from mammograms using texture gradient and Euclidean distance regression.Journal of Digital Imaging. 29(1), 115-125 (2016).
10. Bohare, MD., Cheeran, AN. & Sarode, VG. Analysis of breast mri images using wavelets for detection of cancer. IJCA Special Issue on Electronics, Information and Communication Engineering 4, 1–3 (2011).
11. Brown, S., Tauler, R. & Walczak, B. Comprehensive Chemometrics-Chemical and Biochemical Data Analysis, 2nd Eds. Elsevier (2020).
12. Boukerroui, D., Basset, O., Guerin, N. & Baskurt, A. Multiresolution texture based adaptive clustering algorithm for breast lesion segmentation. European Journal of Ultrasound 8, 135–144 (1998).
13. Bray, F. et al. Global estimates of cancer prevalence for 27 sites in the adult population in 2008. International journal of cancer 132, 1133-1145 (2013).
14. Chen, X. & Wong, S. Cancer Theranostics. doi=https://doi.org/10.1016/C2012-0-02279-X (2014).
15. Cardoso, JS., Marques, N., Dhungel, N., Carneiro, G. & Bradley, P. Mass segmentation in mammograms: acrosssensor comparison of deep and tailored features. IEEE International Conference on Image Processing (ICIP).1737-1741 (2017).
16. Chatzis, SP. & Tsechpenakis, G. The infinite hidden markov random field model. IEEE Transactions On Neural Networks 21(6), 1004–1014 (2010).
17. Chen, W., Giger, ML. & Bick, U. A Fuzzy C-Means (FCM)-Based Approach for Computerized Segmentation of Breast Lesions in Dynamic Contrast-Enhanced MR Images. European Journal of Ultrasound 1, 63-72 (2006).
18. Clark, K., Vendt, B., Smith, K., Freymann, J., Kirby, J., Koppel, P., Moore, S., Phillips, S., Maffitt, D., Pringle, M., Tarbox, L. & Prior, F. The Cancer Imaging Archive (TCIA): Maintaining and Operating a Public Information Repository. Journal of Digital Imaging 26, 1045-1057 (2013).
19. Cross, GR. & Jain, AK. Markov random field texture models. IEEE Transactions On Pattern Analysis And Machine Intelligence 5(1), 25–39 (1983).
20. Patra, DK., Si, T., Mondal, S. & Mukherjee, P. Breast DCE-MRI segmentation for lesion detection by multi-level thresholding using student psychological based optimization. Biomedical Signal Processing and Control. 69, 102925. doi.org/10.1016/j.bspc.2021.102925 (2021).

21. Derrac, J., Garcia, S., Molina, D. & Herrera, F. A practical tutorial on the use of nonparametric statistical tests as a methodology for comparing evolutionary and swarm intelligence algorithms. Swarm and Evolutionary Springer Nature 2021 LATEX template 40 Breast DCE-MRI segmentation using SMAQOBL Computation 1, 3–18 (2001).

22. Desai, SD., Megha, G., Avinash, B., Sudhanva, K., Rasiya, S. & Linganagouda, K. Detection of Microcalcification in Digital Mammograms by Improved-MMGW Segmentation Algorithm.International Conference on Cloud & Ubiquitous Computing and Emerging Technologies. 214-218 (2013).

23. Doi, K. Computer-aided diagnosis in medical imaging: historical review, current status and future potential. Computerized medical imaging and graphics 31, 198-211 (2007).

24. Doi, K. Diagnostic imaging over the last 50 years: research and development in medical imaging science and technology. Physics in medicine and biology 51 (2006).

25. Dong, M., Lu, X., Ma, Y., Guo, Y., Ma, Y. & Wang, K. An efficient approach for automated mass segmentation and classification in mammograms. Journal of Digital Imaging.28(5), 613-625 (2015).

26. Giger, ML., Chan, HP. & Boone, J. Anniversary paper: History and status of CAD and quantitative image analysis: the role of Medical Physics and AAPM. Medical physics 35, 5799-5820 (2008).

27. Gihuijs, KGA., Giger, ML. & Bick, UA. Method for Computerized Assessment of Tumor Extent in Contrast-Enhanced MR Images of the Breast. Computer-Aided Diagnosis in Medical Imaging 27(5), 305-310 (1999) .

28. Hossain, MS. Microcalcification segmentation using modified u-net segmentation network from mammogram images. Journal of King Saud University-Computer and Information Sciences (2019).

29. Ha, W. & Vahedi, Z. Automatic Breast Tumor Diagnosis in MRI Based on a Hybrid CNN and Feature-Based Method Using Improved Deer Hunting Optimization Algorithm. Computational Intelligence and Neuroscience. doi.org/10.1155/2021/5396327 (2021).

30. Hommel, G. A stagewise rejective multiple test procedure based on a modified Bonferroni test. Biometroka 75(2), 383-386. Great Britain (1988).

31. Huynh-Thu, Q. & Ghanbari, M. Scope of validity of PSNR in image video quality assessment. Electronics Letters 44, 800-801. doi =10.1049/el:20080522 (2008).

32. Horsch, A., Hapfelmeier, A. Elter, M. Needs assessment for next generation computer-aided mammography reference image databases and evaluation studies. International journal of computer assisted radiology and surgery 6, 749 (2011).

33. Ibrahim, A., Mohammed, S., Ali, HA. & Hussein, SE. Breast cancer segmentation from thermal images based on chaotic salp swarm algorithm. IEEE Access 8: 122121–122134 (2020).

34. Isa, NAM. & Siong, TS. Automatic segmentation and detection of mass in digital mammograms. Recent researches in communications, signals and information technology 143-146 (2012).

35. Kuhl, CKK., Mielcareck, P., Klaschik, S., Leutner, C., Wardelmann, E. & Gieseke, J. Dynamic breast MR imaging: are signal intensity time data useful for differential diagnosis of enhancing lesions. Radiology 1999 211 (2), 101-110 (1999).

36. Kaur, S., Awasthi, LK., Sangal, AL. & Dhiman, G. Tunicate Swarm Algorithm: A new bio-inspired based metaheuristic paradigm for global optimization. Engineering Applications of Artificial Intelligence. 90,103541 (2020).

37. Kadry, S., Damasevicius, R., Taniar, D., Rajinikanth, V. & Lawal, IA. Extraction of Tumour in Breast MRI using Joint Thresholding and Segmentation – A Study. Seventh International conference on Bio Signals, Images, and Instrumentation (ICBSII). doi.org/10.1109/ICBSII51839.2021.9445152 (2021).

38. Kar, B. & Si, T. Breast DCE-MRI Segmentation for Lesion Detection Using Clustering with Multi-verse Optimization Algorithm. Soft Computing: Theories and Applications. Springer, Singapore. Sharma, T. K., Ahn, C. W., Verma, O. P. & Panigrahi, B. K. Advances in Intelligent Systems and Computing 1381, 265-278. doi.org/10.1007/978-981-16-1696-9$_{2}$5 (2021).

39. Kapur, JN., Sahoo, PK. & Wong, AKC. A new method for gray-level picture thresholding using the entropy of the histogram. Comput. Vis. Graph. Image Process 29(3), 273-285 (1985).

40. Keyvanfard, F., Shoorehdeli, MA., Teshnehlab, M., Nie, K. & Su, M-Y. Specificity enhancement in classification of breast mri lesion based on multi-classifier. Neural Computing and Applications 22(S1), 35–45 (2013).

41. Khalvati, F., Ortiz, CG., Balasingham, S. & Martel, AL. Automated Segmentation of Breast in 3D MR Images Using a Robust Atlas. IEEE Transactions on Medical Imaging 34, 116-125 (2015).

42. Kiani, H., Safabakhsh, R. & Khadangi, E. Fast recursive segmentation algorithm based on Kapur's entropy. 2009 2nd International Conference on Computer, Control and Communication. DOI: 10.1109/IC4.2009.4909269 (2009).

43. Krishnaveni, A., Shankar, R. & Duraisamy, S. Versatile duck traveler optimization algorithm using triple segmentation methods for Springer Nature 2021 LATEX template Breast DCE-MRI segmentation using SMAQOBL mammogram image segmentation to improving accuracy. doi.org/10.2139/ssrn.3803814 (2021).

44. Li, GDS., Dong, M. & Xiaomin, M. Attention dense-u net for automatic breast mass segmentation in digital mammogram.IEEE Access 7, 59037–59047 (2019).

45. Liberman, L., Morris, EA., Benton, CL., Abramson, AF. & Dershaw, DD. MRI of occult breast carcinoma in a high-risk population. Cancer 98(2), 377-388 (2003).

46. Li, S., Chen, H., Wang, M., Heidari, AA. & Mirjalili, S. Slime mould algorithm: A new method for stochastic optimization. Future Generation Computer Systems 111, 300-323 (2020).

47. Lingle, W., Erickson, BJ., Zuley, ML., Jarosz, R., Bonaccio, E., Filippini, J. & Gruszauskas, N. Radiology Data from the Cancer Genome Atlas Breast Invasive Carcinoma Collection [TCGA-BRCA] (2007).

48. Liang, X., Ramamohanara, K., Frazer, H. & Yang, Q. Lesion Segmentation in Dynamic Contrast-Enhanced MRI of Breast. International Conference on Digital Image Computing Techniques and Applications (DICTA), 1-8. doi=10.1109/DICTA.2012.6411734 (2012).

49. Li, H. et al. Computerized radiographic mass detection. II. Decision support by featured database visualization and modular neural networks. IEEE transactions on medical imaging 20, 302-313 (2001).

50. Leichter, I. et al. Optimizing parameters for computer-aided diagnosis of microcalcifications at mammography. Academic radiology 7, 406-412 (2000).

51. Liu, F., Zhang, F., Gong, Z., Chen, Y. & Chai, W. A fully automated scheme for mass detection and segmentation in mammograms. 5th International Conference on Bio Medical Engineering and Informatics. 140-144 (2012).

52. ME, GM. & Subashini, MM. Medical imaging with intelligent systems: A review Sangaiah, A.K. (ed.) Deep Learning and Parallel Computing Environment for Bioengineering Systems, 53–73. Academic Press. Chap. 4. doi.org/10.1016/B978-0-12-816718-2.00011-7 (2019).

53. Mahdavi, S., Rahnamayana, S. & Deb, K. Opposition based learning: A literature review. Swarm and Evolutionary Computation 39, 1-23 (2018).

54. Mohan, J., Krishnavenib, V. & Guo, Y. A survey on the magnetic resonance image denoising methods. Biomedical Signal Processing and Control 9, 56-69 (2014).

55. Merida, AG., Kallenberg, M., Mann, RM., Marti, R. & Karssemeijer, N. Breast Segmentation and Density Estimation in Breast MRI:A fully Automatic Framework .IEEE Journal of biomedical and health informatics 19, 349-357 (2015).

56. Mirjalili, S., Mirjalili, SM. & Hatamlou, A. Multi-verse optimizer: A nature-inspired algorithm for global optimization. Natural Computing and Applications 27, 495-513 (2016).

57. Morris, EA., Liberman, L., Balloon, DJ., Robson, M., Abramson, AF. & Heerdt, A. MRI of occult breast carcinoma in a high-risk population. Am J Roentgenol 3(181), 619–626 (2003).

58. Morris, EA. MRI of occult breast carcinoma in a high-risk population. Semin Ultrasound CT MRI 2003 24(1), 45-54 (2003).

59. Mussurakis, S., Buckley, DL., Coady, AM., Turnbull, LW. & Horsman, A. Observer variability in the interpretation of contrast enhanced MRI of the breast. Br J Radiol 69 (827), 1009-1016 (1996).

60. Mohanty, AK., Senapati, MR. & Lenka, SK. RETRACTED ARTICLE: An improved data mining technique for classification and detection of breast cancer from mammograms. Neural Computing and Applications 22, 303-310 (2013).

61. Nanayakkara, RR., Yapa, YPRD., Hevawithana, PB. & Wijekoon, P. Automatic breast boundary segmentation of mammograms. International Journal of Soft Computing and Engineering.5 (1), 97-101 (2015).

62. Patra, DK., Mondal, S. & Mukherjee, P. Grammatical fireworks algorithm method for breast lesion segmentation in DCE-MRI. International Journal of Innovative Technology and Exploring Engineering 10(7), 170–182. doi.org/10.35940/ijitee.G9054.0510721 (2021).

63. Patra, DK., Si, T., Mondal, S. & Mukherjee, P. Breast DCE-MRI segmentation for lesion detection by multi-level thresholding using student psychological based optimization. Biomedical Signal Processing and Control 69(102925). doi.org/10.1016/j.bspc.2021.102925 (2021).

64. Perona, P. & Malik, J. Scale-space and edge detection using Anisotropic Diffussion. IEEE Trans. Pattern Anal. Mach. Intell 12(7), 629–639 (1990).

65. Pizer, SM., Johnston, RE., Ericksen, JP., Yankaskas, BC. & Muller, KE. Contrast-limited adaptive histogram equalization: speed and effectiveness. Proceedings of the First Conference on Visualization in Biomedical Computing. DOI: 10.1109/VBC.1990.109340 (2002).

66. Rahnamayan, S., Tizhoosh, HR. & Salama, MM. Opposition-based differential evolution. IEEE Trans. Evol. Comput. 12(1), 64–79 (2008).

67. Rahnamayan, S., Wang, GG. & Ventresca, M. An intuitive distance-based explanation of opposition-based sampling. Applied Soft Computing 12(9), 2828–2839 (2012).

68. Rojas-Morales, N., Rojas, M-CR. & Ureta, EM. A survey and classification of Opposition-Based Metaheuristics. Computers and Industrial Engineering 110, 424–435 (2017).

69. Liu, H., Wu, Z., Li, H., Wang, H., Rahnamayan, S., & Deng, C. Rotation-Based Learning: A Novel Extension of Opposition-Based Learning. In: Pham, DN., Park, SB. (eds) PRICAI 2014: Trends in Artificial Intelligence. PRICAI 2014. Lecture Notes in Computer Science(), vol 8862. Springer, Cham.

70. Rampun, A., Morrow, PJ., Scotney, BW. & Winder, J. Fully automated breastboundary andpectoralmuscle segmentation in mammograms. Artificial Intelligence in Medicine 79, 28-41 (2017).

71. Punitha, S., Amuthan, A. & Suresh Joseph, K. Benign and malignant breast cancer segmentation using optimized region growing technique. Future Computing and Informatics Journal3(2).348-358 (2018).

72. Shrivastava, A., Chaudhary, A., Kulshreshtha, D., Singh, VP. & Srivastava, R. Automated digital mammogram segmentation using dispersed region growing and sliding window algorithm.2nd International Conference on Image, Vision and Computing (ICIVC). 366-370 (2017).

73. Saleck, MM., El-Moutaouakkil, A. & Mouçouf, M. Tumor detection in mammography images using fuzzy c-means and glcm texture features.14th International Conference on Computer Graphics, Imaging & Visualization 121–125 (2017).

74. Sadaf, A et al. Performance of computer-aided detection applied to full-field digital mammography in detection of breast cancers. European journal of radiology 77, 457-461 (2011).

75. Samantaray, L., Hembram, S. & Panda, R. A new Harris Hawks-Cuckoo search optimizer for multilevel thresholding of thermogram images. International Information and Engineering Technology Association 34, 541–551. https://doi.org/10.18280/ria.340503 (2020).

76. Shen, R., Yan, K., Xiao, F., Chang, J., Jiang, C. & Zhou, K. Automatic pectoral muscle region segmentation in mammograms using genetic algorithm and morphological selection..Journal of Digital Imaging.31 (5), 680–691 (2018).

77. Si, T., Patra, DK., Mondal, S. & Mukherjee, P. Segmentation of Breast Lesion in DCE-MRI by Multi-level Thresholding using Sine Cosine Algorithm with Quasi Opposition-based Learning. Pattern Analysis and Applications. doi.org/10.1007/s10044-022-01099-8 (2022).

78. , Si, T., Patra, DK., Mondal, S. & Mukherjee, P. Breast DCE-MRI segmentation for lesion detection using Chimp Optimization Algorithm. Pattern Analysis and Applications. 204, 117481. doi.org/10.1016/j.eswa.2022.117481 (2022).

79. Si, T., Miranda, P., Galdino, JV. & Nascimento, A. Grammar-based automatic programming for medical data classification: an experimental study. Artif Intell Rev. 54(3). doi.org/10.1007/s10462-020-09949-9 (2021).

80. Si, T., De, A. & Bhattacharjee, AK. Brain MRI segmentation for tumor detection via entropy maximization using Grammatical Swarm. International Journal of Wavelets, Multiresolution and Information Processing 13: 1550039. doi = https://doi.org/10.1142/S0219691315500393 (2015).

81. Si, T. & Mukhopadhyay, A. Breast DCE-MRI Segmentation for Lesion Detection Using Clustering with Fireworks Algorithm. Applications of Artificial Intelligence in Engineering. Springer, Singapore. Gao, XZ. and Kumar, R. and Srivastava, S. and Soni, B.P. Algorithms for Intelligent Systems 1381: 17-35. doi.org/10.1007/978-981-33-4604-8_2 (2021).

82. Sayed, GI., Soliman, M. & Hassanien, AE. Bio-inspired swarm techniques for thermogram breast cancer detection. In Medical imaging in clinical applications. 487–506 (2016).

83. Soille, P. Morphological Image Analysis: Principles and Applications. Proceedings of the First Conference on Visualization in Biomedical Computing, 173-174 (1999).

84. Schneider, M. & Yaffe, M. Better detection: improving our chances in Digital Mammography: 5th International Workshop on Digital Mammography (2000).

85. Sun, L., He, J., Yin, X., Zhang, Y., Chen, J-H., Kron, T. & Su, M-Y. An image segmentation framework for extracting tumors from breast magnetic resonance images. Journal of Innovative Optical Health Sciences 11(04), 1850014 (2018).

86. Tizhoosh, HR. Opposition-Based Learning: A New Scheme for Machine Intelligence. Proc. Int. Conf. Comput. Intell. Modeling Control and Autom I: 695-701 (2005).

87. Trelea, IC. The particle swarm optimization algorithm: Convergence analysis and parameter selection. Information Processing Letters 85, 317–325 (2002).

88. Tukey, JW. Comparing individual means in the analysis of variance. Biometrics 5(2), 99–114 (1949).

89. Triantaphyllou, E. Multi-Criteria Decision Making Methods: A Comparative Study 44, 3rd edn. Springer. doi.org/10.1007/978-1-4757-3157-6 (2000).

90. Tharwat, A. Classification Assessment Methods. Applied Computing and Informatics 17(1), 168-192. doi.org/10.1016/j.aci.2018.08.003 (2018).

91. Tang, J. et al. Computer-aided detection and diagnosis of breast cancer with mammography: recent advances. IEEE Transactions on Information Technology in Biomedicine 13, 236-251 (2009).

92. Upadhyay, P. & Chhabra, JK. Kapur's entropy based optimal multilevel image segmentation using Crow Search Algorithm. Applied Soft Computing 97, 105522. doi.org/10.1016/j.asoc.2019 (2020)..

93. van Ginneken, B. et al. Comparing and combining algorithms for computer-aided detection of pulmonary nodules in computed tomography scans: the ANODE09 study. Medical image analysis 14, 707-722 (2010).

94. Wang, H., Rahnamayan, S. & Wu, Z. Parallel differential evolution with selfadapting control parameters and generalized opposition-based learning for solving high-dimensional optimization problems. Journal of Parallel and Distributed Computing 73(1), 62–73 (2013).

95. World Health Organization (WHO). Breast cancer. https://www.who.int/news-room/fact-sheets/detail/breast-cancer. Accessed: 02-08-2021 (2021).

96. Wu, Q., Salganicoff, M., Krishnan, A., Fussell, DS. & Markey, MK. Interactive Lesion segmentation on dynamic contrast enhanced breast MR using a Markov Model. Proc. SPIE 6144, Medical Imaging 2006: Image Processing, 61444M (15 March 2006). doi=https://doi.org/10.1117/12.654308 (2006).

97. Xu, Q., Wang, L., Wang, N., Hei, X. & Zhao, L. A review of opposition-based learning from 2005 to 2012. Eng. Appl. Artif. Intell. 29(1), 1–12 (2014) .

98. Zhang, H., Foo, SW., Krishnan, SM. & Hua Thng, C. Computer aided detection of breast masses from digitized mammograms. IEEE International Workshop on Biomedical Circuits and Systems. 1-4 (2004).

99. Zeiser, FA., Da Costa, CA., Zonta, T. et al. Segmentation of masses on mammograms using data augmentation and deep learning. Journal of Digital Imaging.33(4), 858-868 (2020).

100. Westra, C., Dialani, V., Mehta, T.S. & Eisenberg, R. L., Using T2-Weighted Sequences to More Accurately Characterize Breast Masses Seen on MRI. American Journal of Roentgenology. 202(3), W183-W190 (2014)

101. Gao W.-F., Liu S.-Y. & Huang L.-L. Particle swarm optimization with chaotic opposition-based population initialization and stochastic search technique. Commun Nonlinear Sci Numer Simulat 17:4316–4327 (2012)

102. Zhao X, Yang F, Han Y, & Cui Y. An opposition-based chaotic salp swarm algorithm for global optimization. IEEE Access 8:36485–36501 (2020)

103. Bergstra, J. & Bengio, Y. Random Search for Hyper-Parameter Optimization. Journal of Machine Learning Research. 13, 281-305 (2012)

104. Pati, S.K, Gupta, M.K., Banerjee, A., Mallik, S. & Zhao, Z. PPIGCF: A Protein-Protein Interaction-based Gene Correlation Filter for Optimal Gene Selection. Genes. 14(5), 1063 (2023).

105. Pham, N.T., Bunruangses, M., Youplao, P., Garhwal, A., Ray, K., Roy, A., Boonkirdram, S., Yupapin, P., Jalil, M.A.J., Ali, J., Kaiser, S., Mahmud, M., Mallik, S. & Zhao, Z. An Exploratory Simulation Study and Prediction Model on Human Brain Behaviour and Activity using An Integration of Deep Neural Network and Biosensor Rabi Antenna. Heliyon, CellPress. 9(5), e15749 (2023).

106. Bhattacharjee, B., Debnath, B., Das, J.C., Kar, S., Banerjee, N., Mallik & De, D. Predicting the future appearances of lost children for Information forensics with Adaptive discriminator based FLM GAN. Mathematics. 11(6), 1345 (2023).

107. Mallick, K., Chakraborty, S., Mallik, S. & Bandyopadhyay, S. A scalable unsupervised learning of scRNAseq data detects rare cells through integration of structure-preserving embedding, clustering, and outlier detection. Briefings in Bioinformatics. bbad125, https://doi.org/10.1093/bib/bbad125

108. Sahoo, K.K., Ghosh, R., Mallik, S., Roy, A., Singh, P.K. & Zhao, Z. Wrapper-Based Deep Feature Optimization for Activity Recognition in the Wearable Sensor Networks of Healthcare Systems. Nature Scientific Reports. 13, 965, 2023, https://doi.org/10.1038/s41598-022-27192-w

109. Borah, K., Bora, K., Mallik, S. & Zhao, Z. Potential Therapeutic Agents on Alzheimer‘s Disease through Molecular Docking and Molecular Dynamics Simulation study of Plant-based Compounds. Chemistry and Biodiversity (Wiley), 20(1):e202200684 (2023) https://doi.org/10.1002/cbdv.202200684
