# Supplementary figures and images for: Identification of breast lesion through integrated study of gorilla troops optimization and rotation-based learning from MRI images
Source: Sci Rep. 2023 Jul 18;13:11577. doi: 10.1038/s41598-023-36300-3 (PMC10354050; doi:10.1038/s41598-023-36300-3)

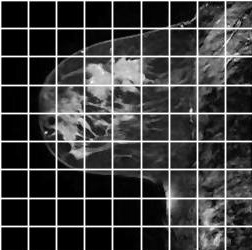

Supplement: Supplementary file 1 — Supplementary Information 1. [file 41598_2023_36300_MOESM1_ESM.zip › Gorilla Troops Optimization MRI Images/17_adf.jpg]

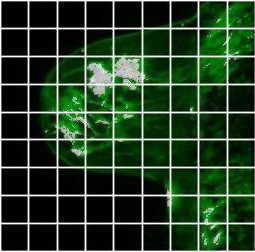

Supplement: Supplementary file 1 — Supplementary Information 1. [file 41598_2023_36300_MOESM1_ESM.zip › Gorilla Troops Optimization MRI Images/17_cmrflocmod.jpg]

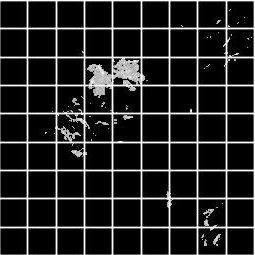

Supplement: Supplementary file 1 — Supplementary Information 1. [file 41598_2023_36300_MOESM1_ESM.zip › Gorilla Troops Optimization MRI Images/17_cmrfmod.jpg]

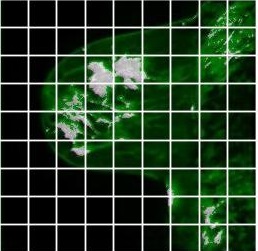

Supplement: Supplementary file 1 — Supplementary Information 1. [file 41598_2023_36300_MOESM1_ESM.zip › Gorilla Troops Optimization MRI Images/17_dalocmod.jpg]

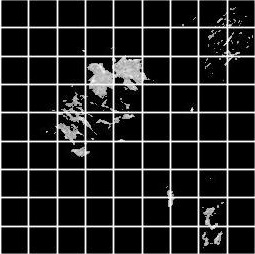

Supplement: Supplementary file 1 — Supplementary Information 1. [file 41598_2023_36300_MOESM1_ESM.zip › Gorilla Troops Optimization MRI Images/17_damod.jpg]

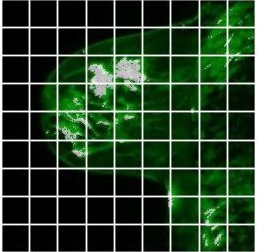

Supplement: Supplementary file 1 — Supplementary Information 1. [file 41598_2023_36300_MOESM1_ESM.zip › Gorilla Troops Optimization MRI Images/17_goalocmod.jpg]

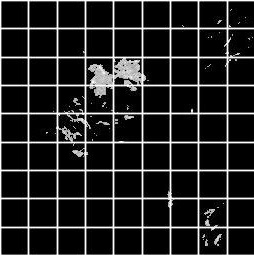

Supplement: Supplementary file 1 — Supplementary Information 1. [file 41598_2023_36300_MOESM1_ESM.zip › Gorilla Troops Optimization MRI Images/17_goamod.jpg]

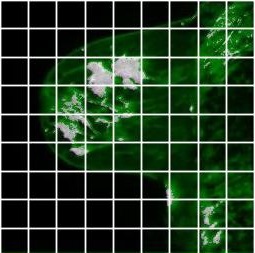

Supplement: Supplementary file 1 — Supplementary Information 1. [file 41598_2023_36300_MOESM1_ESM.zip › Gorilla Troops Optimization MRI Images/17_hmrflocmod.jpg]

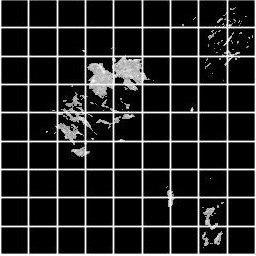

Supplement: Supplementary file 1 — Supplementary Information 1. [file 41598_2023_36300_MOESM1_ESM.zip › Gorilla Troops Optimization MRI Images/17_hmrfmod.jpg]

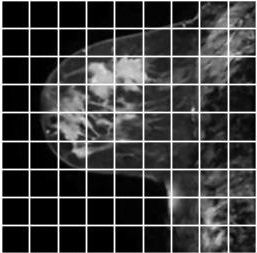

Supplement: Supplementary file 1 — Supplementary Information 1. [file 41598_2023_36300_MOESM1_ESM.zip › Gorilla Troops Optimization MRI Images/17_iih.jpg]

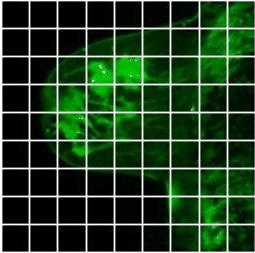

Supplement: Supplementary file 1 — Supplementary Information 1. [file 41598_2023_36300_MOESM1_ESM.zip › Gorilla Troops Optimization MRI Images/17_imrflocmod.jpg]

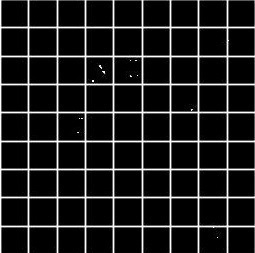

Supplement: Supplementary file 1 — Supplementary Information 1. [file 41598_2023_36300_MOESM1_ESM.zip › Gorilla Troops Optimization MRI Images/17_imrfmod.jpg]

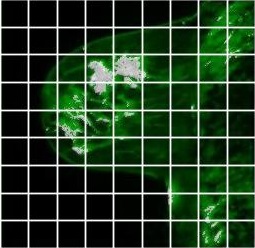

Supplement: Supplementary file 1 — Supplementary Information 1. [file 41598_2023_36300_MOESM1_ESM.zip › Gorilla Troops Optimization MRI Images/17_mvolocmod.jpg]

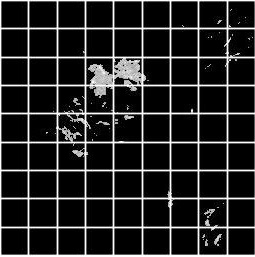

Supplement: Supplementary file 1 — Supplementary Information 1. [file 41598_2023_36300_MOESM1_ESM.zip › Gorilla Troops Optimization MRI Images/17_mvomod.jpg]

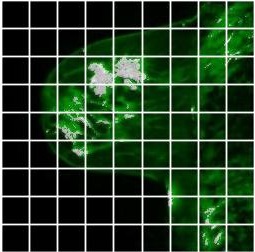

Supplement: Supplementary file 1 — Supplementary Information 1. [file 41598_2023_36300_MOESM1_ESM.zip › Gorilla Troops Optimization MRI Images/17_psolocmod.jpg]

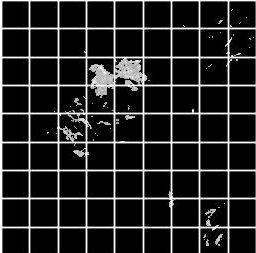

Supplement: Supplementary file 1 — Supplementary Information 1. [file 41598_2023_36300_MOESM1_ESM.zip › Gorilla Troops Optimization MRI Images/17_psomod.jpg]

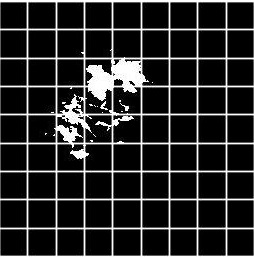

Supplement: Supplementary file 1 — Supplementary Information 1. [file 41598_2023_36300_MOESM1_ESM.zip › Gorilla Troops Optimization MRI Images/17_segmod.jpg]

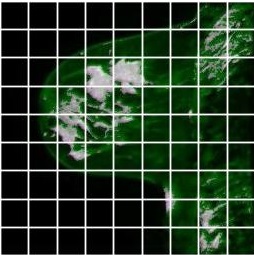

Supplement: Supplementary file 1 — Supplementary Information 1. [file 41598_2023_36300_MOESM1_ESM.zip › Gorilla Troops Optimization MRI Images/17_smalocmod.jpg]

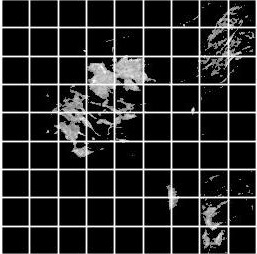

Supplement: Supplementary file 1 — Supplementary Information 1. [file 41598_2023_36300_MOESM1_ESM.zip › Gorilla Troops Optimization MRI Images/17_smamod.jpg]

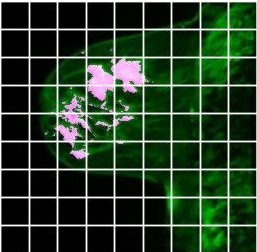

Supplement: Supplementary file 1 — Supplementary Information 1. [file 41598_2023_36300_MOESM1_ESM.zip › Gorilla Troops Optimization MRI Images/17_smaqobllocmod.jpg]

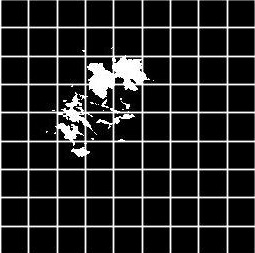

Supplement: Supplementary file 1 — Supplementary Information 1. [file 41598_2023_36300_MOESM1_ESM.zip › Gorilla Troops Optimization MRI Images/17_smaqoblmod.jpg]

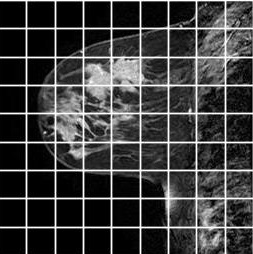

Supplement: Supplementary file 1 — Supplementary Information 1. [file 41598_2023_36300_MOESM1_ESM.zip › Gorilla Troops Optimization MRI Images/17mod.jpg]

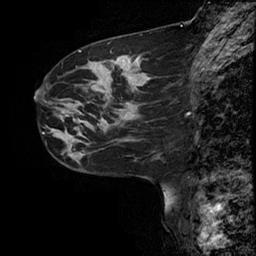

Supplement: Supplementary file 1 — Supplementary Information 1. [file 41598_2023_36300_MOESM1_ESM.zip › Gorilla Troops Optimization MRI Images/19.jpg]

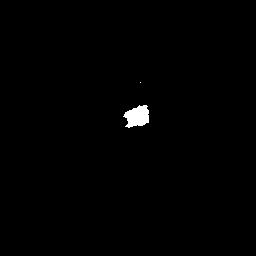

Supplement: Supplementary file 1 — Supplementary Information 1. [file 41598_2023_36300_MOESM1_ESM.zip › Gorilla Troops Optimization MRI Images/4__CHOAqobl.jpg]

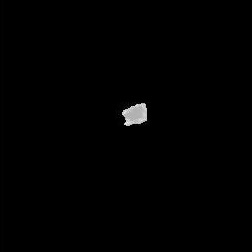

Supplement: Supplementary file 1 — Supplementary Information 1. [file 41598_2023_36300_MOESM1_ESM.zip › Gorilla Troops Optimization MRI Images/4__cmrf.jpg]

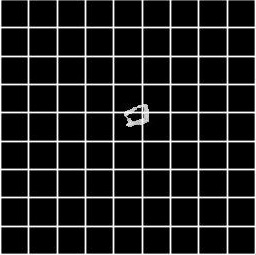

Supplement: Supplementary file 1 — Supplementary Information 1. [file 41598_2023_36300_MOESM1_ESM.zip › Gorilla Troops Optimization MRI Images/4__cmrfmod.jpg]

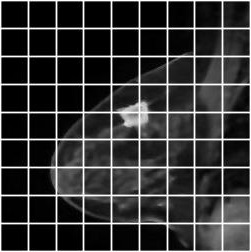

Supplement: Supplementary file 1 — Supplementary Information 1. [file 41598_2023_36300_MOESM1_ESM.zip › Gorilla Troops Optimization MRI Images/4_adf.jpg]

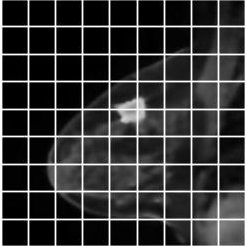

Supplement: Supplementary file 1 — Supplementary Information 1. [file 41598_2023_36300_MOESM1_ESM.zip › Gorilla Troops Optimization MRI Images/4_iih.jpg]

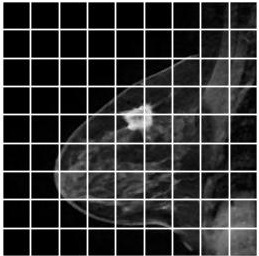

Supplement: Supplementary file 1 — Supplementary Information 1. [file 41598_2023_36300_MOESM1_ESM.zip › Gorilla Troops Optimization MRI Images/4mod.jpg]

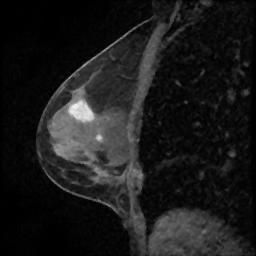

Supplement: Supplementary file 1 — Supplementary Information 1. [file 41598_2023_36300_MOESM1_ESM.zip › Gorilla Troops Optimization MRI Images/7.jpg]

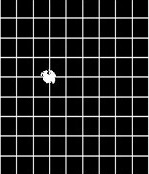

Supplement: Supplementary file 1 — Supplementary Information 1. [file 41598_2023_36300_MOESM1_ESM.zip › Gorilla Troops Optimization MRI Images/9_seggrid.jpg]

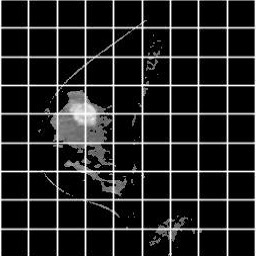

Supplement: Supplementary file 1 — Supplementary Information 1. [file 41598_2023_36300_MOESM1_ESM.zip › Gorilla Troops Optimization MRI Images/9aoagrid.jpg]

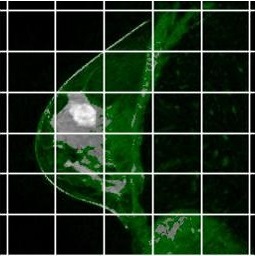

Supplement: Supplementary file 1 — Supplementary Information 1. [file 41598_2023_36300_MOESM1_ESM.zip › Gorilla Troops Optimization MRI Images/9aoaloc.jpg]

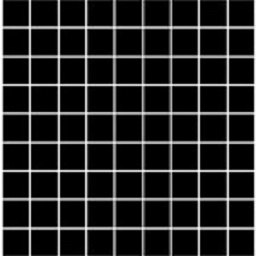

Supplement: Supplementary file 1 — Supplementary Information 1. [file 41598_2023_36300_MOESM1_ESM.zip › Gorilla Troops Optimization MRI Images/9cmrf.jpg]

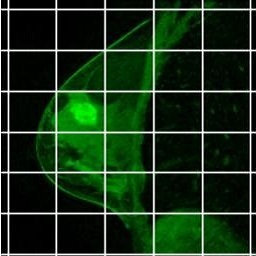

Supplement: Supplementary file 1 — Supplementary Information 1. [file 41598_2023_36300_MOESM1_ESM.zip › Gorilla Troops Optimization MRI Images/9cmrfloc.jpg]

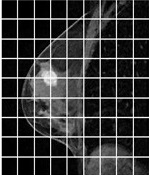

Supplement: Supplementary file 1 — Supplementary Information 1. [file 41598_2023_36300_MOESM1_ESM.zip › Gorilla Troops Optimization MRI Images/9grid.jpg]

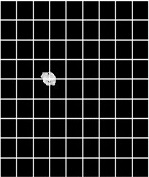

Supplement: Supplementary file 1 — Supplementary Information 1. [file 41598_2023_36300_MOESM1_ESM.zip › Gorilla Troops Optimization MRI Images/9gtogrid.jpg]

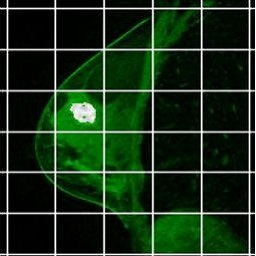

Supplement: Supplementary file 1 — Supplementary Information 1. [file 41598_2023_36300_MOESM1_ESM.zip › Gorilla Troops Optimization MRI Images/9gtoloc.jpg]

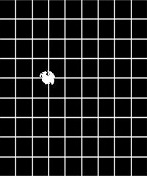

Supplement: Supplementary file 1 — Supplementary Information 1. [file 41598_2023_36300_MOESM1_ESM.zip › Gorilla Troops Optimization MRI Images/9gtorblgrid.jpg]

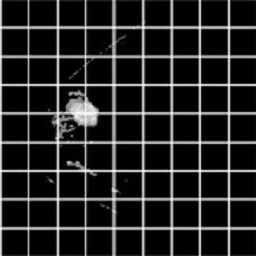

Supplement: Supplementary file 1 — Supplementary Information 1. [file 41598_2023_36300_MOESM1_ESM.zip › Gorilla Troops Optimization MRI Images/9hmrfgrid.jpg]

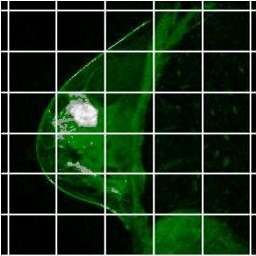

Supplement: Supplementary file 1 — Supplementary Information 1. [file 41598_2023_36300_MOESM1_ESM.zip › Gorilla Troops Optimization MRI Images/9hmrfloc.jpg]

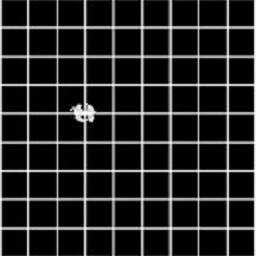

Supplement: Supplementary file 1 — Supplementary Information 1. [file 41598_2023_36300_MOESM1_ESM.zip › Gorilla Troops Optimization MRI Images/9imrfgrid.jpg]

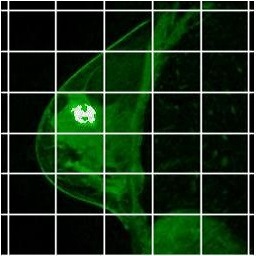

Supplement: Supplementary file 1 — Supplementary Information 1. [file 41598_2023_36300_MOESM1_ESM.zip › Gorilla Troops Optimization MRI Images/9imrfloc.jpg]

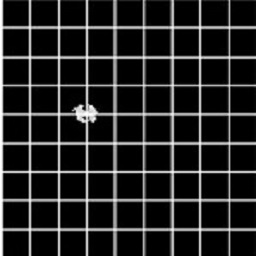

Supplement: Supplementary file 1 — Supplementary Information 1. [file 41598_2023_36300_MOESM1_ESM.zip › Gorilla Troops Optimization MRI Images/9mvogrid.jpg]

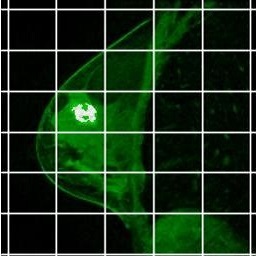

Supplement: Supplementary file 1 — Supplementary Information 1. [file 41598_2023_36300_MOESM1_ESM.zip › Gorilla Troops Optimization MRI Images/9mvoloc.jpg]

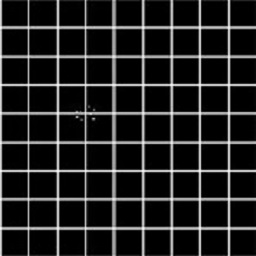

Supplement: Supplementary file 1 — Supplementary Information 1. [file 41598_2023_36300_MOESM1_ESM.zip › Gorilla Troops Optimization MRI Images/9psogrid.jpg]

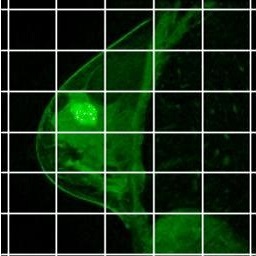

Supplement: Supplementary file 1 — Supplementary Information 1. [file 41598_2023_36300_MOESM1_ESM.zip › Gorilla Troops Optimization MRI Images/9psoloc.jpg]

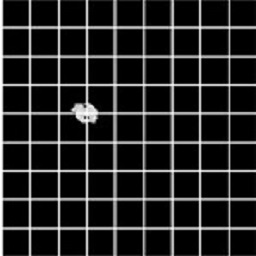

Supplement: Supplementary file 1 — Supplementary Information 1. [file 41598_2023_36300_MOESM1_ESM.zip › Gorilla Troops Optimization MRI Images/9smagrid.jpg]

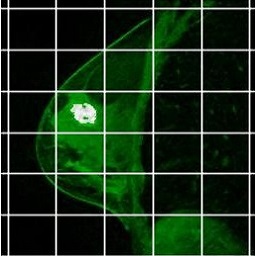

Supplement: Supplementary file 1 — Supplementary Information 1. [file 41598_2023_36300_MOESM1_ESM.zip › Gorilla Troops Optimization MRI Images/9smaloc.jpg]

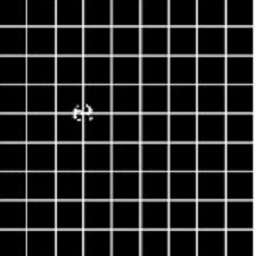

Supplement: Supplementary file 1 — Supplementary Information 1. [file 41598_2023_36300_MOESM1_ESM.zip › Gorilla Troops Optimization MRI Images/9tsagrid.jpg]

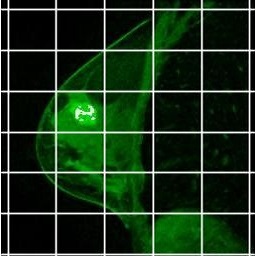

Supplement: Supplementary file 1 — Supplementary Information 1. [file 41598_2023_36300_MOESM1_ESM.zip › Gorilla Troops Optimization MRI Images/9tsaloc.jpg]

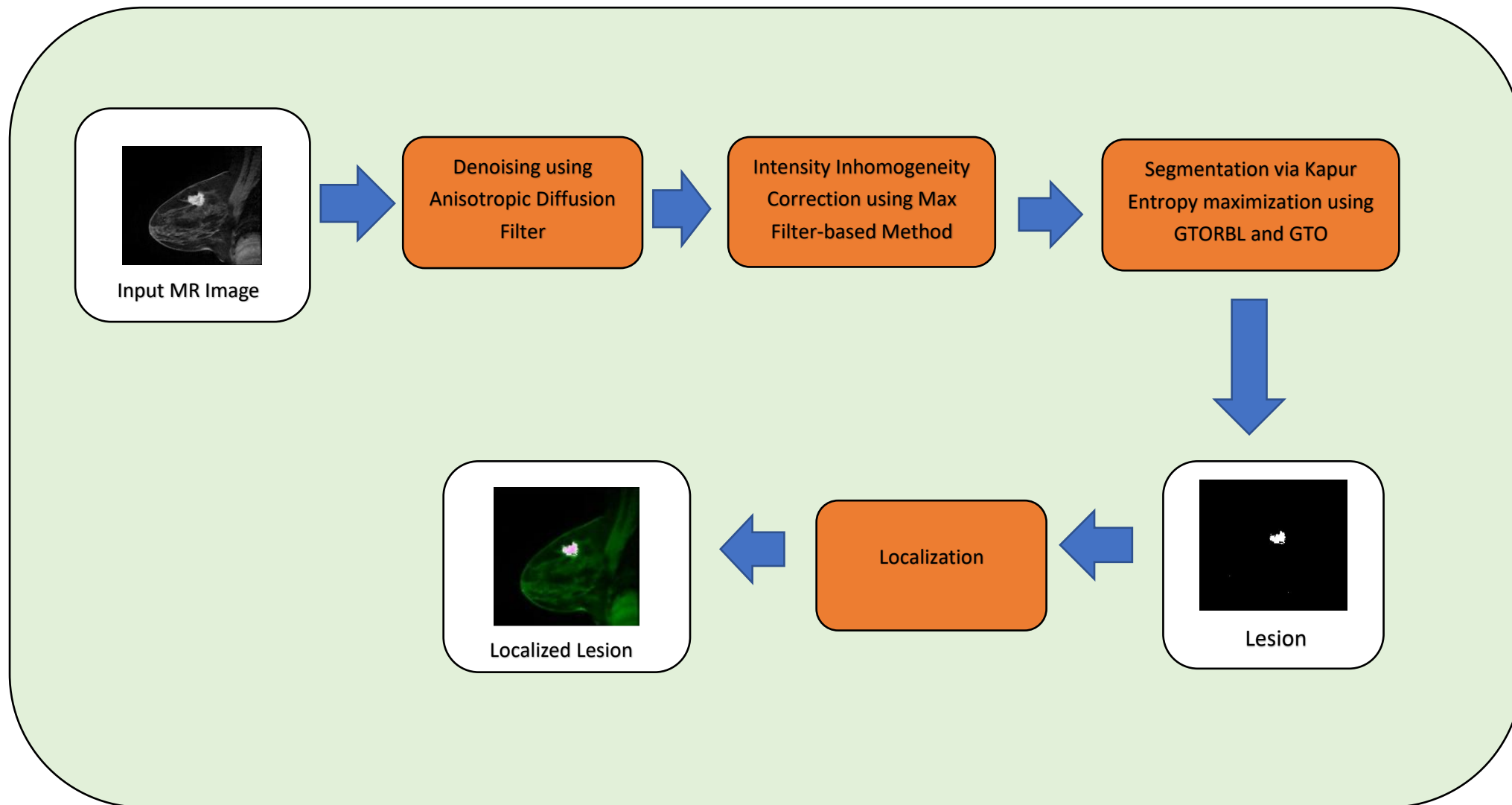

Supplement: Supplementary file 1 — Supplementary Information 1. [file 41598_2023_36300_MOESM1_ESM.zip › Gorilla Troops Optimization MRI Images/Docgtorbl.pdf]

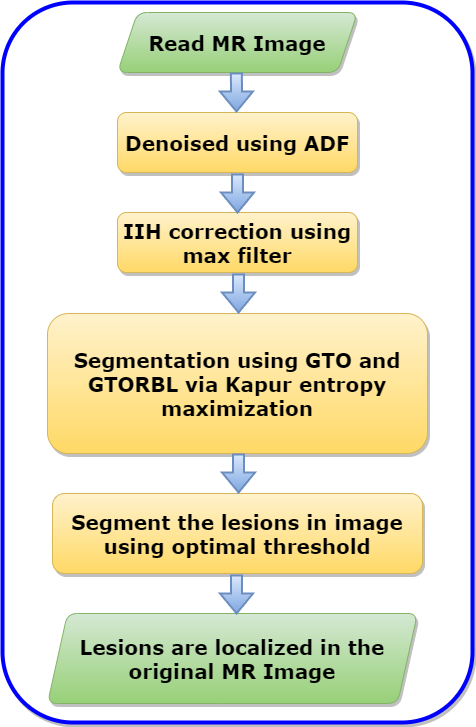

Supplement: Supplementary file 1 — Supplementary Information 1. [file 41598_2023_36300_MOESM1_ESM.zip › Gorilla Troops Optimization MRI Images/flowchart_proposal.png]

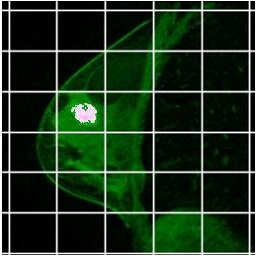

Supplement: Supplementary file 1 — Supplementary Information 1. [file 41598_2023_36300_MOESM1_ESM.zip › Gorilla Troops Optimization MRI Images/gtorblloc.jpg]

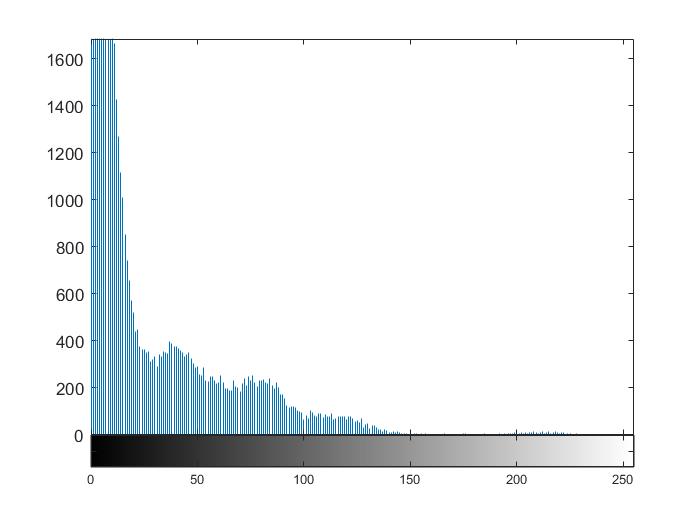

Supplement: Supplementary file 1 — Supplementary Information 1. [file 41598_2023_36300_MOESM1_ESM.zip › Gorilla Troops Optimization MRI Images/hist3.jpg]

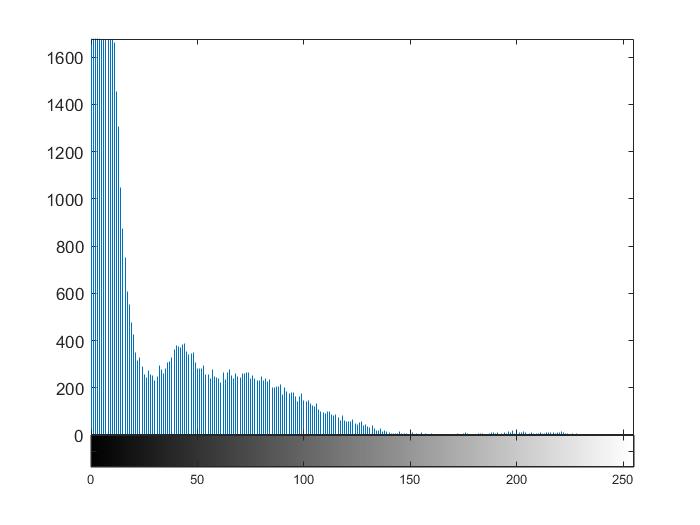

Supplement: Supplementary file 1 — Supplementary Information 1. [file 41598_2023_36300_MOESM1_ESM.zip › Gorilla Troops Optimization MRI Images/hist4.jpg]

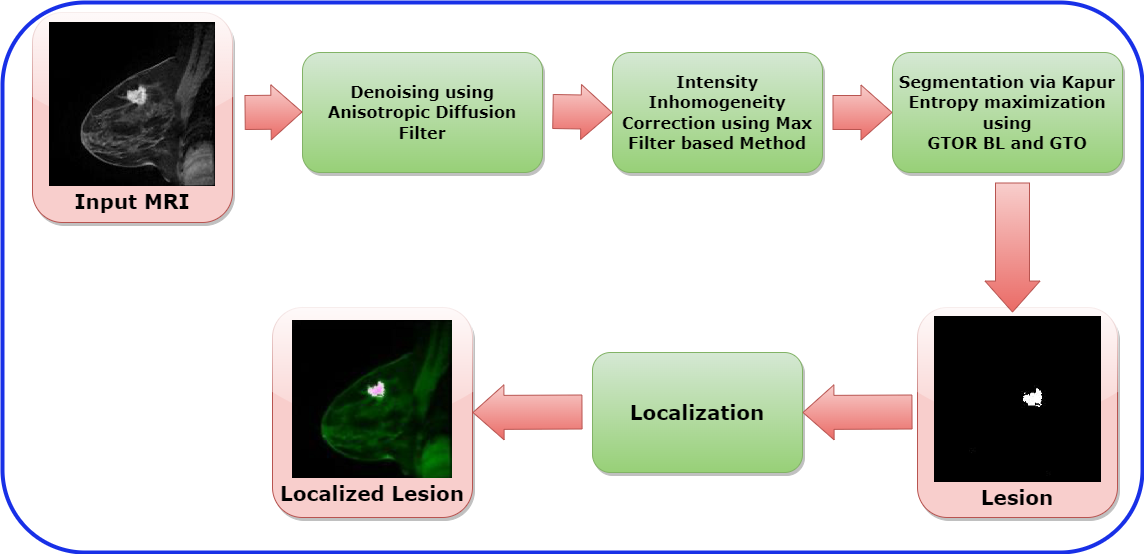

Supplement: Supplementary file 1 — Supplementary Information 1. [file 41598_2023_36300_MOESM1_ESM.zip › Gorilla Troops Optimization MRI Images/outline.png]

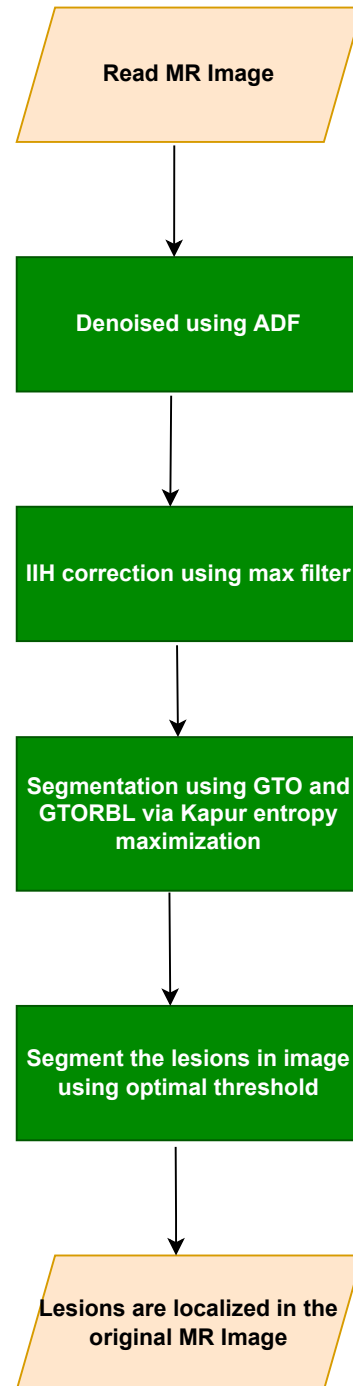

Supplement: Supplementary file 1 — Supplementary Information 1. [file 41598_2023_36300_MOESM1_ESM.zip › Gorilla Troops Optimization MRI Images/overallflow (1).pdf]

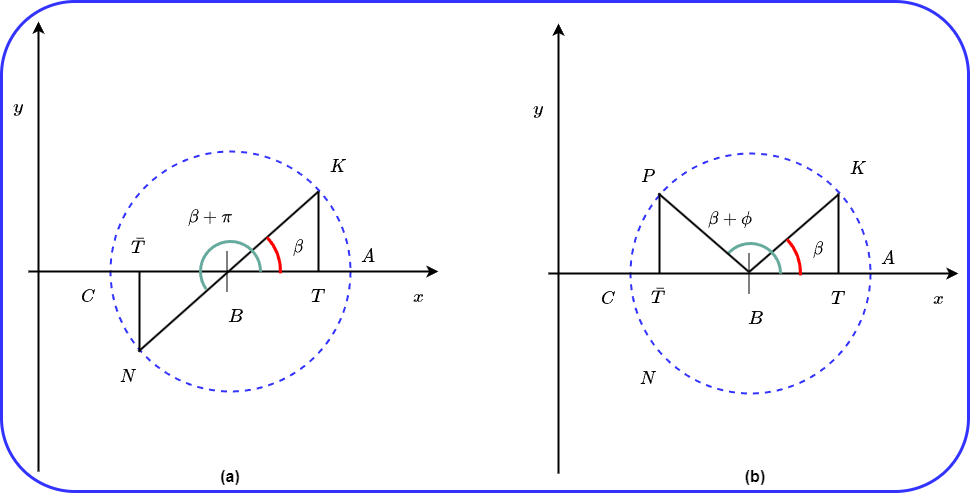

Supplement: Supplementary file 1 — Supplementary Information 1. [file 41598_2023_36300_MOESM1_ESM.zip › Gorilla Troops Optimization MRI Images/rbl.png]

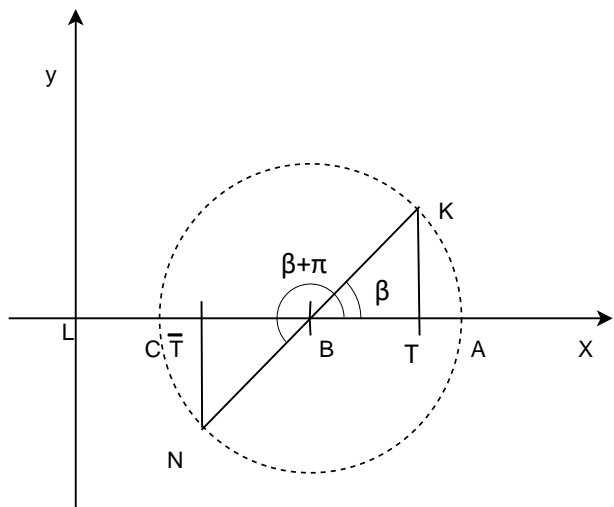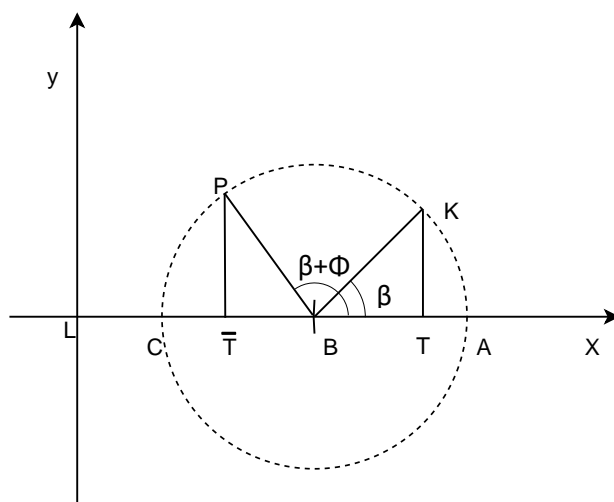

Supplement: Supplementary file 1 — Supplementary Information 1. [file 41598_2023_36300_MOESM1_ESM.zip › Gorilla Troops Optimization MRI Images/rbl2d.pdf]

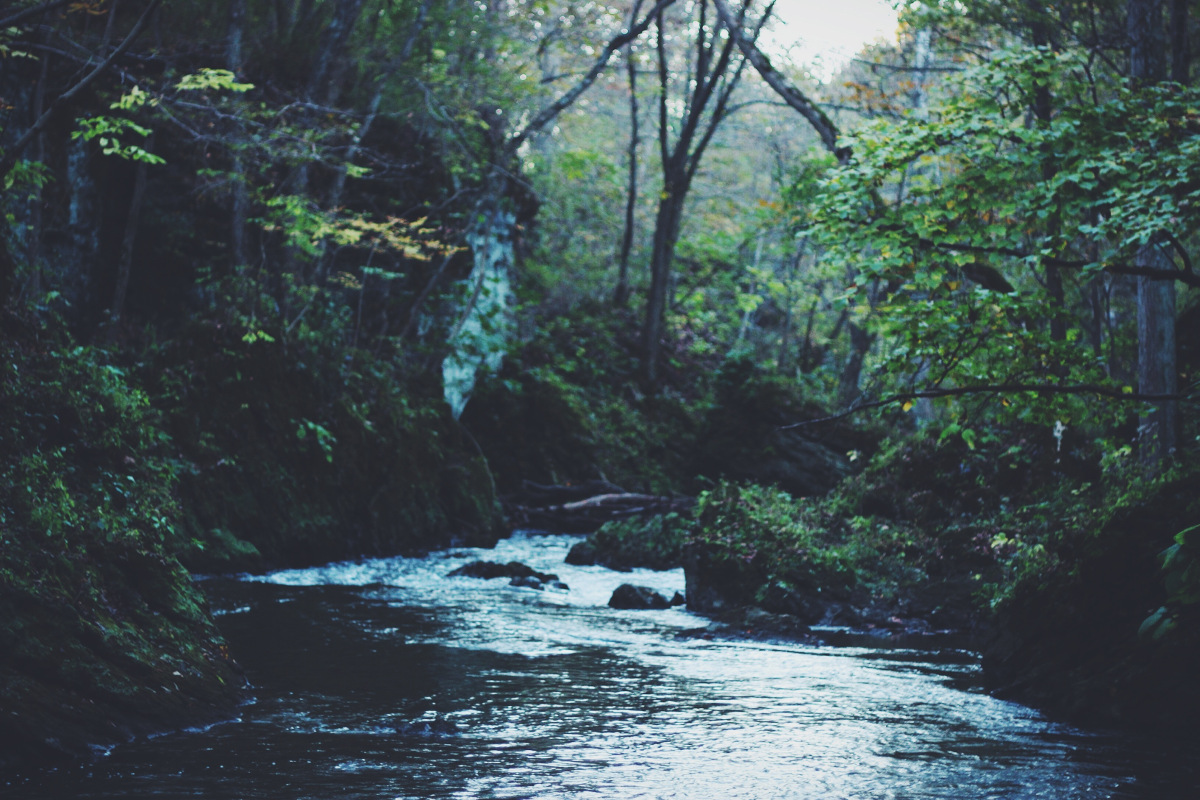

Supplement: Supplementary file 1 — Supplementary Information 1. [file 41598_2023_36300_MOESM1_ESM.zip › Gorilla Troops Optimization MRI Images/stream.jpg]
